# Presolar silicon carbide grains from a CO3 meteorite

**DOI:** 10.1140/epja/s10050-026-01959-5

**Published:** 2026-10-04

**Authors:** Emilie T. Dunham, Michael R. Savina, Ming-Chang Liu, Gregory A. Brennecka, Peter K. Weber, Julie M. Korsmeyer, Andrew M. Davis, Philipp R. Heck, Thomas Stephan, Maximilian Ehrenfels, Autumn G. Roberts

**Affiliations:** 1 Physical and Life Sciences Division, Lawrence Livermore National Laboratory, Livermore, CA 94550 USA; 2 Department of Earth, Planetary, and Space Sciences, University of California, Los Angeles, Los Angeles, CA 90095 USA; 3 Department of the Geophysical Sciences, The University of Chicago, Chicago, IL 60637 USA; 4 Chicago Center for Cosmochemistry, Chicago, IL USA; 5 Robert A. Pritzker Center for Meteoritics and Polar Studies, Negaunee Integrative Research Center, Field Museum of Natural History, Chicago, IL 60605 USA; 6 Department of Chemistry, The University of Chicago, Chicago, IL 60637 USA; 7 Division of Solar System Science and Exploration, Southwest Research Institute, Boulder, CO 80302 USA; 8 Enrico Fermi Institute, The University of Chicago, Chicago, IL 60637 USA; 9 Jackson School of Geosciences, The University of Texas at Austin, Austin, TX 78712 USA; 10 Department of Geological Sciences, University of Colorado Boulder, Boulder, CO 80309 USA

## Abstract

Presolar silicon carbide (SiC) grains found in primitive meteorites can help constrain circumstellar condensation processes and nucleosynthesis in C-rich low-mass asymptotic giant branch stars and core collapse supernovae. We performed the first presolar grain chemical separation procedure on primitive CO3 meteorite Northwest Africa 7892 and identified 45 presolar SiC grains. These grains fit into the previously defined presolar SiC groups in similar proportions. We used a new secondary ion mass spectrometer (NanoSIMS XR prototype) to obtain high spatial resolution images of the grains. Three of the grains have isotopically distinct hotspots: regions 150–500 nm in diameter whose $^{12}$C/$^{13}$C ratios differ from that of the host grain by >5σ. Though the origin of the hotspots is unclear, these nanoscale features demonstrate the potential for new discoveries with the ultrabright $\mathrm{Cs^+}$ make it superscript source in the NanoSIMS XR prototype.

**Supplementary Information:**

The online version contains supplementary material available at https://doi.org/10.1140/epja/s10050-026-01959-5.

## Introduction

Stardust grains are condensates that formed in the cooling outflows of stars during the late stages of stellar evolution. A fraction of this circumstellar dust was incorporated into the solar nebula and escaped the high temperatures of planetary formation. These surviving presolar grains are preserved in primitive meteorites, where they retain elemental, isotopic, and structural signatures of their stellar birth environments. As such, presolar grains provide direct constraints on dust condensation in stellar outflows, stellar nucleosynthesis, and the astrophysical environment of the Solar System.

Presolar grains are identified by isotopic compositions that are highly anomalous relative to Solar System materials, reflecting the nucleosynthetic conditions in their parent stars [1, 2]. Presolar SiC, graphite, oxide, and silicate grains form in a variety of stellar sources, including low-mass asymptotic giant branch (AGB) stars, core-collapse supernovae, novae, and other stellar environments. Although presolar SiC is not the most abundant presolar grain phase, it is among the most extensively studied because it is comparatively easy to extract and identify [3]. Presolar SiC grains have been divided into nine isotopic groups [4], of which the five most common are Mainstream, AB, Y, Z, and X. Mainstream (88%), Y (4%), and Z (1%) grains formed in 1.5–4 $\textit{M}_\odot$ C-rich AGB stars with different metallicities, X grains (1%) originated in Type II supernovae, and AB grains are thought to derive from J-type carbon stars, born-again AGB stars, and/or Type II supernovae [5–10].

Presolar grains are valuable time capsules because their isotopic compositions record details of stellar nucleosynthesis, leading to constraints on the type of parent star that sourced the grain. Light element isotopes, such as C, N, and Si, are fingerprints of stellar fusion, whereas isotopic compositions of heavy elements such as Mo, Ru, Sr, Zr, and Ba, reflect the *s*-, *r*-, and *p*-processes. A big challenge in uncovering the isotopic information from presolar SiC grains is that they are analytically difficult to study because they are rare (typically ≤30 ppm of the host meteorite) and small (with average diameters of only ∼200 nm) [3]. These characteristics place strong demands on sample preparation and analytical instrumentation.

The first presolar grain extractions and analyses were performed in the 1980s and 1990s by Lewis et al. [11], Bernatowicz et al. [12], and Amari et al. [13]. The development of nanoscale secondary ion mass spectrometry (nanoSIMS) revolutionized the study of presolar grains beginning in the early 2000s [14]. Its high spatial resolution (∼100 nm beam size at a few pA) and high sensitivity capabilities enabled efficient measurement of light-element isotopic compositions in large numbers of grains. As a result, approximately 20,000 presolar SiC grains, primarily from CM meteorites, have been measured for their C and Si isotopic compositions [4].

In addition to analysis of whole grains, the internal microstructures of presolar grains can provide insight into their formation histories. Stardust condensation in cooling AGB outflows may proceed by direct condensation or by vapor deposition onto seed grains [15]. Transmission electron microscopy (TEM) studies of presolar graphite [16–21] and SiC [22] have revealed embedded subgrains, including refractory carbides such as TiC and Fe–Ni metal phases. These subgrains are typically tens of nm in diameter or smaller, therefore, are difficult to characterize with nanoSIMS isotopically, yet such measurements are important for testing whether they served as nucleation sites for the host grains. Some spatial isotopic heterogeneities correlated to known subgrains have been found via nanoSIMS in graphite grains [18, 21]. Some isotopic variation in SiC grains has been observed as well, however, it may be due to contamination and not subgrains [23].

Two main approaches have been used to identify presolar grains from meteorites. Historically, grains were isolated by chemical dissolution techniques [24], followed by isotopic analysis using SIMS. While this approach enabled the discovery and characterization of many presolar grains, it is inherently selective because it is dependent on the chemical resistance of specific minerals. For example, presolar silicates are destroyed early during the chemical separation process. In addition, such separations require relatively large sample masses, commonly more than 5 g, which can be prohibitive for limited meteorite material.

The alternative approach for identifying presolar grains is through nanoSIMS isotope mapping of meteorite sections. This method avoids chemical treatment, consumes minimal material, and, in principle, allows all isotopically anomalous presolar grain types to be identified. However, surveying large areas of meteorite matrix can require days to weeks of instrument time [25, 26]. This time- and labor-intensive approach, then, makes it practically challenging to efficiently find presolar grains, especially the rarer types (e.g., large grains, X grains). Moreover, once a presolar grain is recognized in situ, it remains embedded in the surrounding matrix, which can complicate subsequent SIMS or resonance ionization mass spectrometry (RIMS) measurements because of interference from adjacent material.

For the present study, we focus on chemical separation in order to isolate large presolar SiC grains for coordinated scanning electron microscopy (SEM) and nanoSIMS analysis. This strategy is motivated by planned measurements of trace-element isotopic compositions by RIMS [27–30], as well as noble gas isotopic analyses to investigate cosmic-ray exposure histories [31]. RIMS benefits from analyses on relatively larger grains, while noble gas analyses requires large grains in order to have sufficient material. Chemical separation methods have been applied to meteorites spanning a range of petrologic types, but only a few primitive meteorites, including Murchison (Mighei-type: CM2), Murray (CM2), Aguas Zarcas (CM2), Orgueil (Ivuna-type: CI1), Qingzhen (EH3), and Tieschitz (H/L3.6), have undergone extensive processing designed to isolate SiC, graphite, and nanodiamond presolar grains [24, 32–38]. Of all meteorites studied, Murchison has been the dominant source of presolar SiC grains because of its primitiveness, high presolar grain abundance, and large available mass; indeed, 92% of presolar SiC grains studied to date come from Murchison [4].

The dominance of Murchison in the existing SiC database highlights the need to expand presolar grain studies to other primitive meteorite groups to determine whether differences among grain populations exist. Selecting a meteorite that is likely to have many presolar grains is important. Studies that measured presolar SiC abundances by noble gas stepped pyrolysis indicate that the highest matrix-normalized SiC abundances occur at roughly 20–30 ppm in CM2.5, CO3.0 (Ornans-type), and LL3.0 meteorites [3, 39, 40]. Meteorites of higher petrologic type contain substantially lower presolar SiC grain abundances; for example, the single CO3.2 meteorite, Kainsaz (reclassified from CO3.1 to CO3.2 in 2006), measured has an inferred abundance of only 0.1 ppm, about 200 times lower than CO3.0 meteorites [3, 39]. Primitive CO chondrites therefore represent promising but underexplored targets for presolar SiC studies.

In this work, we present a new sample set of presolar SiC grains chemically isolated from the primitive CO3 meteorite, Northwest Africa (NWA) 7892. Focusing on the large grain fraction (>3 µm), we identify candidate SiC grains by SEM and isotopically characterize them by nanoSIMS. This study expands the inventory of large presolar SiC grains from non-CM meteorites and establishes a sample set suitable for future nm-scale, trace-element, and noble gas isotopic investigations. In addition to a 2002-era CAMECA NanoSIMS 50, we make use of a new CAMECA NanoSIMS XR prototype with a beam measured to be approximately 25 nm in diameter by SEM (Supplemental Document). The LoTIS Cs$^{+}$ ion source [41] in the NanoSIMS XR prototype is nominally capable of 10 nm lateral resolution at 2 pA, substantially smaller diameter and more intense than the Cs$^{+}$ primary beams of previous nanoSIMS instruments [42]. This improved spatial resolution offers new opportunities to resolve subgrain-scale isotopic variations in presolar materials.

## Samples and methods

### Presolar grain separation

#### Sample

We selected the CO3 meteorite NWA 7892 for this study, as it has a low petrologic type, indicating presolar grain preservation. In addition, a large mass was readily available. This sample’s sub-type is the subject of recent discussion. NWA 7892 was originally classified as a CO3.0 in the Meteoritical Bulletin [43], using olivine σCr$_{2}$O$_{3}$ vs. wt% Cr$_{2}$O$_{3},$ though the 0.39±0.19 wt% result lies above the trend of most primitive COs [44]. A recent study [44] measured the organic matter in the matrix of NWA 7892 via Raman spectroscopy because organic matter is sensitive to relatively low peak temperatures, and the results can more accurately determine the metamorphic grade of the least altered chondrites (<3.2). This work suggests that NWA 7892 is a CO3.1, though the sample lies off the trend through other primitive COs [44]. Therefore, it is unclear whether NWA 7892 is a CO3.0 or CO3.1; this distinction may be important, because the CO3.2 meteorite Kainsaz has a much lower abundance of presolar SiC grains (0.1 ppm) compared to CO3.0s (20 ppm) [40].

The meteorite was cut with a diamond wire saw and roughly polished using SiC sanding paper (this was not known prior to separation). An uncut or non-SiC polished starting material would have been preferable. A mass of 24.66 g from two slices of NWA 7892 was used. This is the first extensive presolar grain separation using the method by Amari et al. [24] performed on a CO3 meteorite, allowing the estimation of presolar grain populations sourced from a very mildly thermally-altered parent body.

**Fig. 1:**
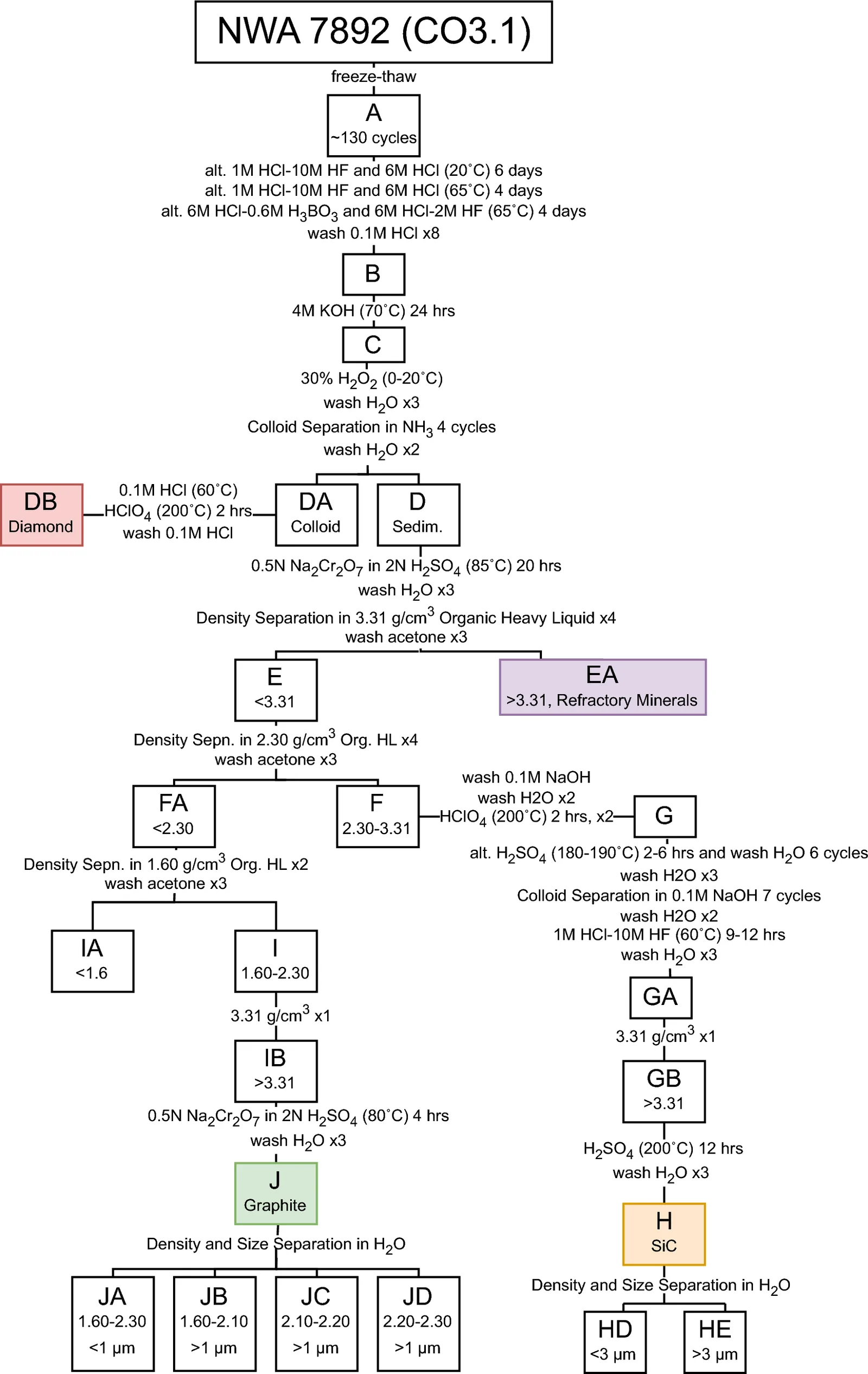
Flow chart describing the presolar grain separation method, modified from [24, 34]. The goal of the procedure is to produce fractions of nanodiamonds, oxide minerals (spinel, hibonite, and corundum), graphite, and SiC. In the flow chart, the fractions are labeled with one or two-letter identifiers, with the numbers below indicating the density range (g/cm$^{3}$) or size limit (µm)

#### Chemistry

We utilized insights and methodology developed at the University of Chicago [24, 34] to set up a separation procedure at Lawrence Livermore National Laboratory (LLNL). The method we used is from Korsmeyer [34], the “Modified Chicago Method,” adapted for our CO3 meteorite sample. The main difference between the procedures described by Korsmeyer [34] and Amari et al. [24] is the use of organic heavy liquids (Cargille Laboratories; brominated aliphatics [45]) instead of sodium polytungstate to separate the particles by density. The procedure aims to maximize the recovery of many grain types: SiC, graphite, diamond, and oxides (spinel, hibonite, corundum), while minimizing the exposure of the grains to possible heavy element contaminating solvents. The detailed flow chart of the separation is given in Fig. 1. The main steps of the method are as follows:

1. Disaggregate the meteorite gently using 130 freeze–thaw cycles. No remaining large particles were found or removed.
2. Destroy silicates, sulfides, metal, phosphates, sulfates, carbonates, and other material with alternating HF and HCl cycles. Any remaining fluorides are then removed with $\mathrm{H_{3}BO_{3}}.$
3. Destroy kerogen, otherwise known as insoluble organic matter (IOM), and sulfur with KOH followed by H_2_O_2_.
4. Separate nanodiamond colloid with NH_4_OH and then acidify with HCl and let settle.
5. Clean remaining particles with heated Na_2_Cr_2_O_7_ in H_2_SO_4_ (chromic acid).
6. Use organic heavy liquid with density of 3.31 g/cm$^{3}$ to separate minerals that have a higher density than SiC (∼3.2 g/cm$^{3}$), such as spinel (∼3.6 g/cm$^{3}$), hibonite (∼3.8 g/cm$^{3}$), and corundum (∼4.0 g/cm$^{3}$).
7. On the lower density fraction, use organic heavy liquid with density 2.30 g/cm$^{3}$ to separate the SiC (∼3.2 g/cm$^{3}$) fraction and graphite fraction (∼1.6–2.2 g/cm$^{3}$).
8. On the graphite fraction, use organic heavy liquid with density 1.60 g/cm$^{3}$ to remove low density organics.
9. Remove organics from SiC and nanodiamond fractions with heated HClO_4_.
10. Clean SiC fraction with heated H_2_SO_4_, NaOH, HF, and HCl.
11. On the graphite and SiC fractions, perform a 3.31 g/cm$^{3}$ density separation to remove more oxides. Treat SiC fraction with heated H_2_SO_4_ to dissolve remaining oxides.
12. Divide the graphite fraction by density and size using centrifugation.
13. Perform a size separation on the SiC fraction using centrifugation.

All chemicals used were high purity (Seastar Chemical and Optima Grade Fisher Scientific). After the final processing steps, the separates were then suspended in a solution of Milli-Q water and isopropanol and pipetted onto ultrapure gold foil pressed onto 1/2 inch diameter aluminum pin mounts [34]. Grains were not pressed into the gold, so that they could be moved for future measurements.

### Scanning electron microscopy

Secondary electron (SE) imaging and energy-dispersive X-ray spectroscopy (EDS) measurements were performed with a Tescan Mira Field Emission Gun (FEG) SEM equipped with an Oxford Instruments Ultim Max Infinity EDS detector system. EDS maps of the particle mounts were obtained at 10 keV with the following settings: 5–10 nA, 2048 × 2048 pixels, 10 μs dwell time per pixel, 4 frames per image. SE and back-scattered electron (BSE) maps were also acquired. The field of view was approximately 300 µm × 300 µm per frame. Similar settings were used for EDS spot elemental analysis of Si, C, O, Mg, and Al of individual SiC grains. We layered these maps with QGIS software [46] and used the Si elemental map to identify SiC grain candidates. Spot EDS analyses for C, O, and Si were used to distinguish SiC grains from other Si-rich grains. SE and BSE images of each candidate grain are included in the Supplementary Document.

For a few of the mounts, we tried a more efficient, automated way to locate Si-rich grains from EDS maps. We developed a Python script that loads the Si, C, and O maps, allows the user to define concentration thresholds for each element, and identifies particles that exceed the Si and C thresholds while falling below the O threshold. This automated filtering greatly speeds up the search for SiC grains. After this, we still verified grain SiC composition with individual EDS spots.

ImageJ software [47] was used to determine the size of each grain. After setting the scale, we used the freehand selection tool to encircle the edge of the grain (thresholding was usually not possible due to other particles in the image). We calculated the apparent diameter (Table 1) from the area assuming that the grain is spherical:

1 $$\begin{aligned} \text {diameter} = 2\times \sqrt{\text {area}/\pi }. \end{aligned}$$

**Fig. 2:**
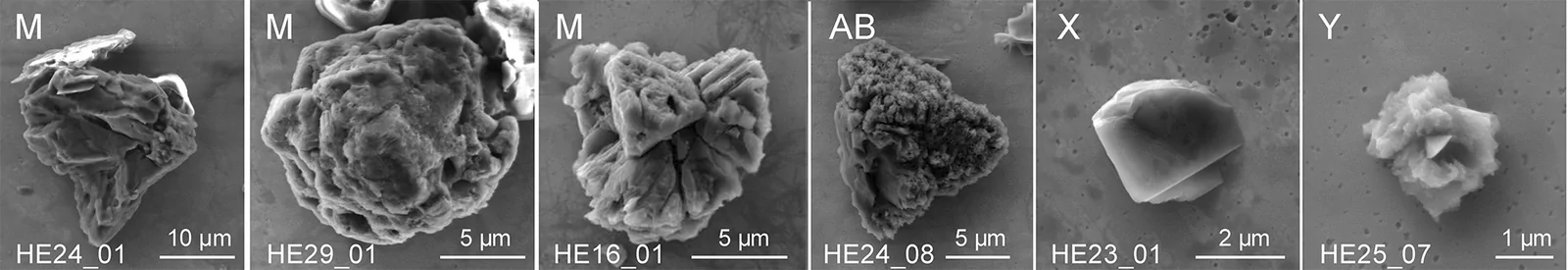
Secondary electron images of selected presolar SiC grains, in order of decreasing size from left to right. The grain name and type are shown on each image. Oxide grains adjacent to the SiC appear brighter in the image. Images of all 45 presolar grains are available in the Supplemental Document

### Isotopic measurements: Si, C, N

SiC candidates found by EDS were then measured for their light element isotopic composition by nanoSIMS to determine whether they are presolar and, if so, what presolar grain type they are. Measurements were carried out in two sessions. During both sessions, SiC and Si$_{3}$N$_{4}$ standards were measured before, in between, and after measurements of presolar samples. In the first session, C and Si isotopes were analyzed with the CAMECA NanoSIMS 50 at LLNL. A 16 pA Cs$^{+}$ primary beam was used to raster a 3–20 µm field over individual grains, generating $^{12}\text{ C}^{-}$, $^{13}\text{ C}^{-}$, $^{28}\text{ Si}^{-}$, $^{29}\text{ Si}^{-}$, and $^{30}\text{ Si}^{-}$ secondary ions, which were collected simultaneously with five electron multipliers. A mass resolving power (MRP; M/Δ M at 10% peak height) of 4500 was obtained by introducing entrance slit (ES) 2 and aperture slit (AS) 1 to allow for the separation of hydrides from the peaks of interest (e.g., $^{12}\text{ CH}^{-}$ from $^{13}\text{ C}^{-}$, and $^{28}\text{ SiH}^{-}$ from $^{29}\text{ Si}^{-}$). A minimum of 3 min of pre-analysis sputtering with a higher current beam was applied to locate SiC grains and reach sputter equilibrium before data acquisition. The NanoSIMS 50 analyses were done in isotope mode, in which the beam is scanned over the sample 100 times with 32×32 pixel raster and the data are summed for each scan. The NanoSIMS 50 beam was ∼150 nm; however, its exact diameter was not determined.

In the second session, C, N, and Si isotopic compositions were measured in imaging mode using NanoSIMS XR prototype at LLNL, based on the latest-generation nanoSIMS instrument manufactured by CAMECA. The instrument has NanoSIMS-HR functionality [48] as well as a LoTIS (Low Temperature Ion Source) Cs$^{+}$ source [41]. The LoTIS source is brighter compared to a conventional CAMECA thermal Cs$^{+}$ source, allowing for a more tightly focused primary beam at any given current, which enables higher spatial resolution at comparable count rates. Prior to analysis and to locate the grains more efficiently, we used a higher current beam (∼1 nA) and larger raster for about 10 s of initial pre-analysis sputtering to implant Cs$^{+}$ so that sputter equilibrium could be reached more quickly during analyses. The current was then reduced to 2 pA Cs$^{+}$ and the raster was reduced to the size of the grain. The image resolution was estimated to be approximately 25 nm based on SEM analysis of individual fixed-beam craters (Supplemental Document).

After reaching sputtering equilibrium and optimizing secondary beam centering, each particle was analyzed for 5-cycles (65.5 s/cycle) of isotope data: $^{12}\text{ C}^{-}$, $^{13}\text{ C}^{-}$, $^{12}\text{ C}^{14}\text{ N}^{-}$, $^{12}\text{ C}^{15}\text{ N}^{-}$, $^{28}\text{ Si}^{-}$, $^{29}\text{ Si}^{-}$, $^{30}\text{ Si}^{-}$. All secondary ions were counted simultaneously by seven electron multipliers at a mass resolving power of ∼7500 (at 10% height; ES4, AS3). This MRP is higher than that during the NanoSIMS 50 session, in order to measure N isotopes.

The images taken with the NanoSIMS XR prototype were taken at a resolution of 256 × 256 pixels. Because grains have a range of sizes, the analysis raster size was varied from ∼3 to 20 µm to encompass the grain. This means that while the spot size was constant, the pixel spacing varied from about 12 nm/pixel to 78 nm/pixel, respectively. With the largest raster size, there is some space between beam spots, which is not ideal as the lack of beam overlap can negatively influence the sputter equilibrium. However, our data indicate that there was not any significant unaccounted fractionation with the larger pixel spacing. At 12 nm/pixel, the ideal fifty percent overlap of the beam spots during the raster occurs. As we describe in the results, some of the SiC grains found in the meteorite residue have terrestrial composition and are likely to be contamination from sample processing (surface polishing), which used carborundum (SiC). These grains have a large range of sizes and are generally platy with flat surfaces. Since they are recognizable by morphology, they can function adventitiously as an extra set of standards. While this is not a perfect way to rule out isotope fractionation, we find that the platy-looking SiC grains measured with nanoSIMS raster sizes from 5–20 µm (similar to presolar SiC grains) have isotope compositions within uncertainty of terrestrial isotopic composition, indicating negligible instrumental fractionation caused by non-overlapping raster spots.

#### Standardization and data corrections

We used SiC and Si$_{3}$N$_{4}$ grains manufactured by Ted Pella as standards. The standard grains have an average diameter of 10 µm, though some are as small as a few µm, comparable to our presolar grains. The industrially produced SiC grains are platy and angular in shape with nearly flat surfaces, making them poor morphological proxies for presolar SiC grains, which are typically rounded with textured surfaces. This issue also exists for the $>20{,}000$ previously measured presolar SiC grains in the Presolar Grain Database [4]. Some of the standard grains were pressed into the gold substrate, while others were sitting on the surface; both scenarios yielded the same isotopic ratios within uncertainty.

The data corrections were calculated in the same way for the NanoSIMS 50 spot-analyses and the images taken with the NanoSIMS XR prototype. The image data of the standards and presolar grains was extracted using the L’Image package (Larry Nittler’s Image Program: limagesoftware.net). Isotope ratios were derived from images using the total counts of each isotope measured in a grain. The standard data were used to determine the instrumental mass fractionation factor as well as data reproducibility over a given day. The day-to-day reproducibility of the standards varied between 0.5–1.0% RSD for C, 0.2–0.8% for Si and 3–5% for N. The Si isotope values are given as delta values, defined:

2 $$\begin{aligned} \delta ^{i,j}\text {Si} = \Big [\frac{(^{i}\text {Si}/^{j}\text {Si})_{\text {grain}}}{(^{i}\text {Si}/^{j}\text {Si})_{\text {standard}}} -1 \Big ]\times 1000\permil , \end{aligned}$$

with NBS28 used as the standard, i = 29 or 30 and j = 28 (normalizing isotope).

Uncertainties for the C, Si, and N isotopes were calculated by adding in quadrature the Poisson counting statistical error of each grain and the standard deviations obtained from standard analyses. The uncertainties are comparable to those of other SIMS studies [4].

**Fig. 3:**
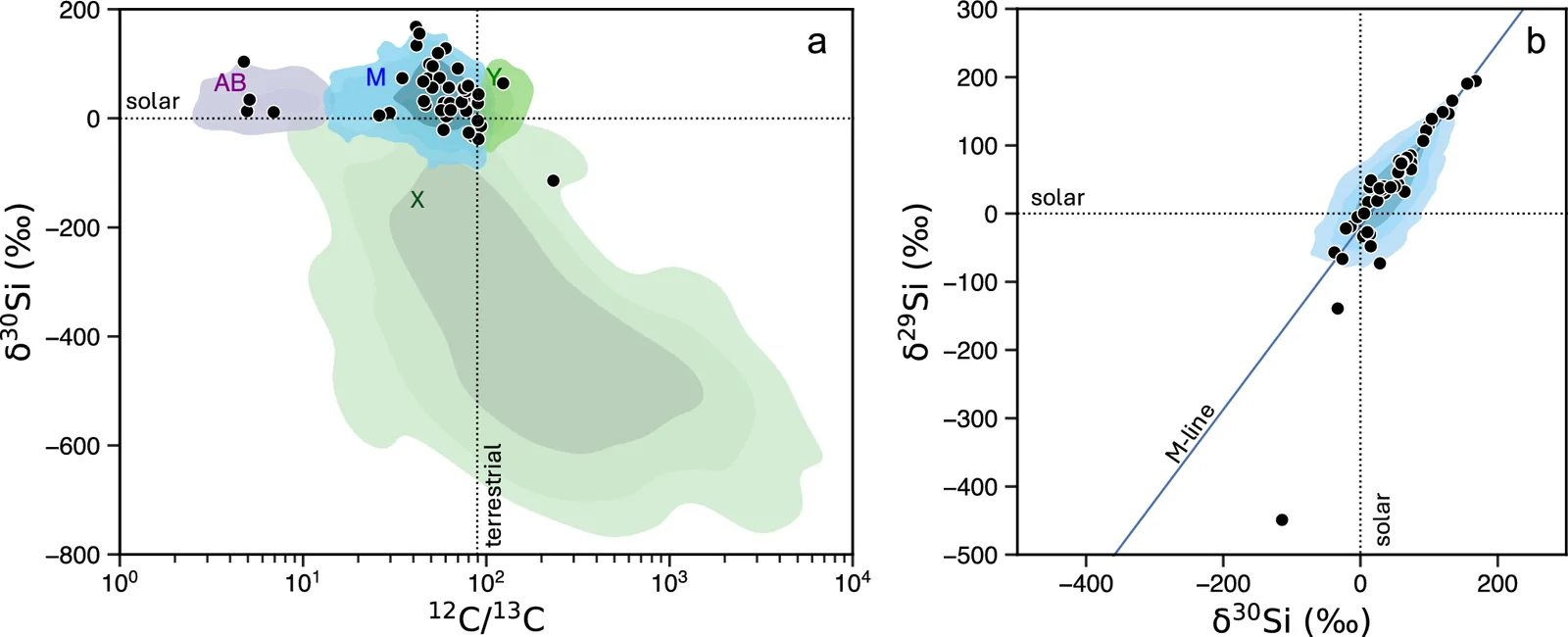
NWA 7892 presolar SiC grain $^{12}$C/$^{13}$C versus $\delta ^{30}$Si isotope composition (**a**) and Si 3-isotope plots (**b**). Uncertainties are 1σ. The areas show literature data [4] for mainstream (M), AB, Y, and X grains, where each shade represents 1, 2, and 3σ data distribution envelopes. Data from this study is represented as black circles. Blue line in **b** is the mainstream (M) line [4]. Black dotted lines indicate solar composition. Uncertainties are 1σ

## Results

### Presolar grain separation

We selectively dissolved two slices of meteorite NWA 7892 with the goal of obtaining presolar nanodiamonds, oxide minerals, graphite, and SiC. After cleaning IOM from the nanodiamond fraction, the suspension appears tan-colored in water. A preliminary nanodiamond abundance of >100 ppm was determined from the weight of the nanodiamond fraction, and is reported as a lower limit because not all portions of the nanodiamond fraction have been cleaned and weighed.

Residues from the SiC fraction at step GA (Fig. 1) contained SiC candidates alongside oxide grains like spinel. Despite an additional density separation at 3.31 g/cm$^{3}$ and dissolution step with sulfuric acid, many of the oxide grains persist. This results in difficulty identifying SiC candidates, because there is an overwhelmingly higher proportion of oxides. For example, the highest number of SiC grains identified on a mount prepared from the HE fraction (> 3 µm) was 15, and oxide grains are often near or touching the SiC grains. Amari et al. [24] reported that the coarsest fractions (>∼ 3 µm) of their Murchison K series have large amounts of oxide grains (corundum, hibonite and other phases), while smaller size fractions do not.

Other than the oxides, our SiC fractions are relatively clean of contaminant phases like IOM. In comparison, the CM2 residues of Aguas Zarcas, prepared by Korsmeyer et al. [34] with the same procedure as this work, have IOM contamination, which can interfere with grain identification and subsequent elemental and isotopic measurements. One factor may be that CO chondrites have about a third as much IOM as CM chondrites [49].

**Fig. 4:**
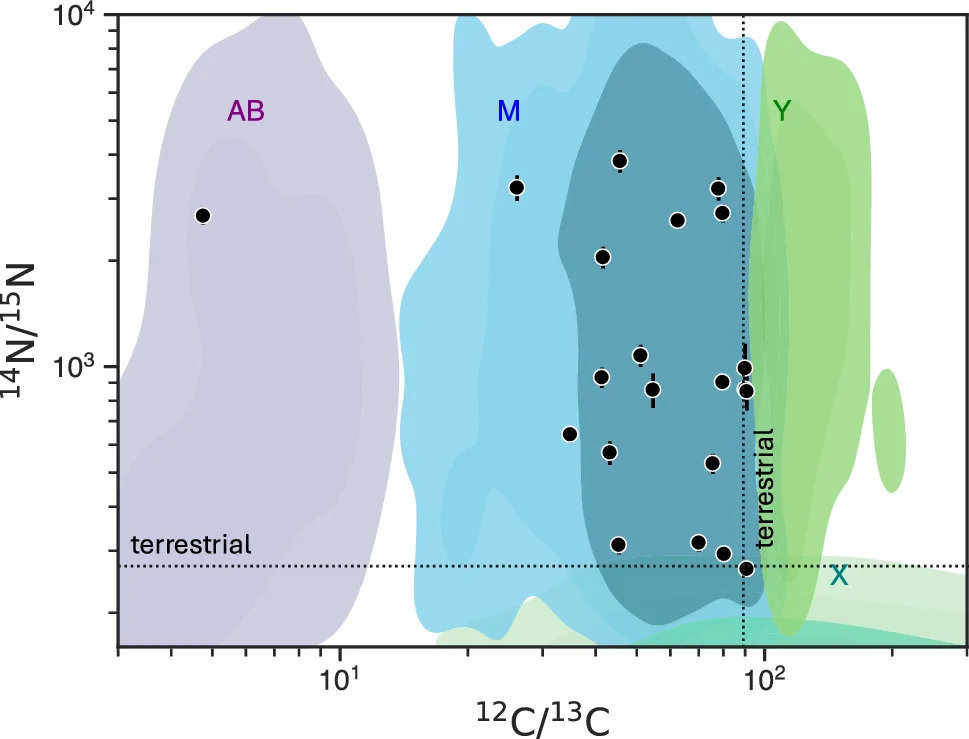
NWA 7892 presolar SiC grain $^{12}$C/$^{13}$C versus $^{14}$N/$^{15}$N isotope ratios measured with LLNL’s NanoSIMS XR prototype. The colored areas are literature data distribution envelopes with shades representing 1, 2, and 3σ [4] for mainstream (M), AB, Y, and X grains. Data from this study is represented as black circles, with 1σ uncertainties. Black dotted lines indicate terrestrial C and N isotope ratios

The highest density fraction (>3.2 g/cm$^{3}$) residue contains spinel, corundum, hibonite, and other oxide minerals of varying sizes. From the graphite fraction (1.6–2.3 g/cm$^{3}$), we have identified two graphite candidates using SEM-EDS so far. Hereafter, this paper will focus on the SiC grain population and its implications.

### NWA 7892 presolar SiC grain types

We identified 100 SiC grains across 17 mounts using SEM (example grains shown in Fig. 2). We conducted two nanoSIMS sessions to analyse 65 of these candidates, 35 on the NanoSIMS 50 (C and Si isotopes) and 30 on the NanoSIMS XR prototype (C, Si, and N isotopes).

Since our sample was exposed to terrestrial SiC during rough polishing prior to our obtaining it, we are cautious in classifying our SiC candidates as presolar. As previously mentioned, two distinct morphologic SiC groups are present in the analyzed residues; more than half of the SiC grains look like previously characterized presolar grains (i.e., rounded shape, aggregated structure, and surface pitting) [50], while the remaining grains appear more morphologically similar to terrestrial SiC grains (i.e., platy, blocky, and angular shapes). When analyzed, the grains from this second group typically have C, Si, and N isotopic compositions within 3σ of terrestrial (Table 1). Hereafter we label such grains “terrestrial.” Grains are classified as presolar when their C, Si, and/or N isotope ratios are outside 3σ of solar composition (Figs. 3, 4). Of the 65 SiC grains measured, we confidently classify 45 as presolar based on anomalous C, Si, and N isotopes as well as morphology (Fig. 2).

We used the presolar SiC grain classification according to Stephan et al. [4] to determine the grain types for this dataset. Different grain types are typically defined by ranges or lower/upper limits in isotopic ratios. For example, mainstream grains have $^{12}\text {C}/^{13}\text {C}$ between 13.5 to 100, $^{14}\text {N}/^{15}\text {N}$ >200, with Si isotopes falling within a band along the mainstream line (represented by “M-line” in Fig. 3). From these definitions, we calculate the most probable grain type for each measured SiC grain. We identified 39 mainstream grains (87%), four AB grains (9%), one Y grain (2%), and one X grain (2%). Of the AB grains, one is of subtype AB2, and the X grain is of subtype X2. The grain types are shown in Figs. 3 and 4 and listed in Table 1.

**Table 1:** Presolar and terrestrial SiC grain C, Si, and N isotopic data

Name	Type$^{\textrm{a}}$	NS$^{\textrm{b}}$	Diam (μm)	$^{12}\text {C}/^{13}\text {C}$	1σ	$\delta ^{29}\text {Si} \ (\permil )$	1σ	$\delta ^{30}\text {Si} \ (\permil )$	1σ	$^{14}\text {N}/^{15}\text {N}$	1σ
CO_HE08_07	AB	50	2.0	5.10	0.03	39.6	1.9	34.2	2.2	2685	150
CO_HE08_11	AB	50	2.7	4.95	0.03	38.6	5.8	13.3	8.8	NA	NA
CO_HE24_08	AB	50	13.0	6.92	0.04	16.7	5.8	11.3	8.6	NA	NA
CO_HE29_05	AB2	XR	2.4	4.75	0.03	138.8	3.6	103.9	2.6	2685	150
CO_HE29_05_1	hotspot	XR	0.2	6.41	0.08	87.1	20.7	85.0	24.8	1752	233
CO_HE29_05_2	hotspot	XR	0.2	6.08	0.05	141.7	11.3	133.0	13.4	2087	303
CO_GA07_01	M	XR	2.7	29.65	0.19	− 27.2	5.6	10.0	7.3	8002	562
CO_GA07_09	M	XR	3.4	34.79	0.22	65.1	5.5	73.7	7.9	643	34
CO_GA07_12	M	XR	7.3	90.67	0.66	− 57.0	5.8	− 38.0	7.5	267	13
CO_HE08_04	M	50	2.8	50.77	0.36	77.5	5.9	56.3	8.8	NA	NA
CO_HE08_05	M	50	1.9	58.44	0.32	− 21.9	1.2	− 21.3	1.4	NA	NA
CO_HE08_06	M	50	1.6	63.87	0.44	48.7	3.2	15.3	3.7	NA	NA
CO_HE09_05	M	50	2.5	56.74	0.83	− 48.0	4.0	14.9	6.2	NA	NA
CO_HE09_06	M	50	7.0	84.71	1.21	− 139.2	3.9	− 33.2	6.3	NA	NA
CO_HE14_04	M	XR	3.9	79.54	0.41	73.5	5.1	59.6	4.9	905	39
CO_HE16_01	M	50	10.6	47.78	0.30	85.2	5.8	73.0	8.7	NA	NA
CO_HE18_03	M	XR	11.3	80.16	0.45	− 66.5	5.4	− 26.4	5.4	294	11
CO_HE18_04	M	XR	5.1	43.15	0.21	190.4	5.3	155.5	6.9	571	44
CO_HE19_02	M	XR	3.9	26.08	0.16	0.2	4.9	5.3	5.2	3227	269
CO_HE20_01	M	XR	4.4	69.92	0.46	106.6	4.9	91.4	3.9	317	18
CO_HE23_03	M	50	3.4	55.57	0.79	74.5	4.0	74.0	6.3	NA	NA
CO_HE23_04	M	50	4.8	60.01	0.86	146.5	4.0	128.4	6.4	NA	NA
CO_HE23_06	M	50	2.4	58.69	0.87	− 73.0	4.2	28.3	6.3	NA	NA
CO_HE23_08	M	50	2.3	93.53	1.34	− 19.1	4.1	− 14.2	6.4	NA	NA
CO_HE23_09	M	50	1.7	75.62	1.08	30.1	4.0	35.4	6.4	NA	NA
CO_HE24_01	M	50	21.9	60.50	0.85	− 33.2	3.7	3.9	6.2	NA	NA
CO_HE24_10	M	50	2.1	77.73	0.51	− 30.6	5.9	13.8	8.7	NA	NA
CO_HE25_02	M	50	2.4	46.58	0.30	18.7	5.9	24.5	8.7	NA	NA
CO_HE25_03	M	50	2.1	63.19	0.40	39.4	5.8	27.9	8.6	NA	NA
CO_HE25_04	M	50	2.7	74.93	0.47	43.4	5.7	54.9	8.6	NA	NA
CO_HE25_08	M	50	1.9	73.32	0.55	36.6	6.1	30.0	8.8	NA	NA
CO_HE25_09	M	50	2.4	48.85	0.30	129.0	5.8	99.5	8.6	NA	NA
CO_HE28_01	M	XR	7.4	62.40	0.41	60.5	5.6	56.4	7.2	2603	135
CO_HE28_02	M	XR	2.8	41.58	0.29	165.5	3.1	133.7	3.0	2048	142
CO_HE28_02_1	hotspot	XR	0.3	55.72	1.01	122.5	13.0	95.6	15.4	692	156
CO_HE28_03	M	XR	3.2	41.31	0.28	194.4	2.7	167.9	2.5	933	62
CO_HE28_04	M	XR	3.5	50.98	0.35	121.7	4.5	95.7	4.2	1077	77
CO_HE28_06	M	XR	2.6	45.60	0.32	35.2	4.5	31.5	3.3	3841	281
CO_HE28_07	M	XR	2.4	77.76	0.62	39.9	4.7	50.1	3.9	3207	244
CO_HE28_08	M	XR	4.6	75.43	0.55	60.5	3.0	54.9	3.4	532	35
CO_HE29_01	M	XR	14.4	54.49	0.38	148.8	3.1	119.8	3.1	861	96
CO_HE29_02	M	XR	5.3	89.99	0.64	36.9	2.9	27.8	2.5	863	67
CO_HE29_03	M	XR	6.1	89.80	0.67	− 5.1	3.1	− 4.3	3.2	991	172
CO_HE29_06	M	XR	4.4	45.27	0.31	81.8	2.9	67.5	2.9	312	18
CO_HE29_06_1	hotspot	XR	0.5	56.38	1.42	52.7	17.6	48.0	21.0	264	27
CO_HE29_06_2	hotspot	XR	0.3	58.64	1.22	84.4	14.2	59.1	16.7	301	30
CO_HE29_07	M	XR	1.9	90.68	0.72	38.5	3.7	44.0	3.2	852	99
CO_HE29_08	M	XR	2.0	79.54	0.57	74.9	3.7	60.9	3.1	2733	160
CO_HE23_01	X	50	3.0	232.22	3.96	− 449.1	9.1	− 114.3	6.8	NA	NA
CO_HE25_07	Y	50	2.2	123.56	0.81	32.0	5.8	64.3	8.6	NA	NA
CO_GA07_32	terr	XR	5.8	88.81	0.59	2.3	5.5	0.1	7.3	279	18
CO_GA08_01	terr	50	11.4	92.88	2.85	17.2	15.3	1.7	17.5	NA	NA
CO_HE14_01	terr	XR	11.2	88.45	0.40	− 11.5	4.9	− 16.3	4.7	263	12
CO_HE14_01	terr	XR	3.6	88.64	0.35	− 9.9	5.0	− 11.2	5.1	267	11
CO_HE14_03	terr	XR	4.3	90.24	0.48	− 10.7	5.2	− 6.3	5.5	277	12
CO_HE18_01	terr	XR	4.0	87.79	0.43	11.2	5.0	2.8	4.8	235	28
CO_HE19_01	terr	XR	18.8	90.68	0.56	1.3	4.6	6.0	3.4	275	15
CO_HE19_03	terr	XR	10.7	88.54	0.62	16.3	4.9	4.9	4.1	286	22
CO_HE20_02	terr	XR	22.4	94.32	0.58	2.3	4.6	3.8	3.5	270	13
CO_HE23_05	terr	50	15.0	89.17	1.27	0.2	4.0	0.6	6.3	NA	NA
CO_HE24_02	terr	50	2.3	90.79	1.07	7.4	5.8	21.1	8.7	NA	NA
CO_HE24_03	terr	50	3.1	90.48	1.28	− 12.0	3.9	− 4.9	6.3	NA	NA
CO_HE24_05	terr	50	2.0	94.43	1.35	− 8.6	3.9	13.8	6.3	NA	NA
CO_HE24_06	terr	50	2.0	87.88	1.27	8.8	4.0	28.8	6.3	NA	NA
CO_HE24_07	terr	50	3.5	91.20	1.29	− 11.7	3.9	4.5	6.3	NA	NA
CO_HE24_09	terr	50	5.7	89.62	0.57	− 15.3	5.7	3.7	8.6	NA	NA
CO_HE24_12	terr	50	2.2	88.50	0.55	− 0.8	5.7	1.4	8.6	NA	NA
CO_HE25_05	terr	50	6.0	90.49	0.57	− 12.7	5.7	− 10.5	8.6	NA	NA
CO_HE25_06	terr	50	3.6	88.32	0.55	− 7.8	5.7	6.1	8.6	NA	NA
CO_HE25_10	terr	50	3.0	89.86	0.64	− 12.3	5.9	− 15.5	8.8	NA	NA

$^{\textrm{a}}$Type: AB, AB2, mainstream (M), X, Y, spot = isotopically distinct spot, terr = terrestrial

$^{\textrm{b}}$NS: nanoSIMS type; XR = NanoSIMS XR prototype; 50 = NanoSIMS 50

### NWA 7892 presolar SiC grain concentration

Previous presolar SiC abundances from meteorite separations were determined by either noble gas measurements or in situ analyses, and not by individual particle counting from SEM data. Therefore, any abundances for NWA 7892 presolar SiC calculated at this point would not be directly comparable to literature values from past separations. Additionally, it is difficult to determine an accurate concentration of grains with only 45 identified, all of which were from the large size fraction. To get a broad sense of NWA 7892’s presolar SiC concentration, though, we compare the number of large grains identified. We found 5 large, >10 µm, presolar SiC grains in a quarter of the SiC HE fraction; extrapolating this, we estimate a total of 20 large presolar grains from the 24.66 g NWA 7892 sample. This equates to 0.8 large grains per gram in the bulk meteorite.

### NWA 7892 SiC presolar grain characteristics

The apparent diameters of the 45 presolar SiC grains from the HE large size fraction (>3 µm) range from 1.6 to 21.9 µm, with an average of 4.5 µm and median of 2.8 µm (Fig. 2). The sizes were determined using high-resolution SEM images. Some grains smaller than the desired cutoff were found, as various factors can affect the precision of size separations by centrifugation [34].

The presolar SiC grains are commonly rounded in shape, although some have a more euhedral texture. Most of the presolar SiC grains have a fine-scale, rough texture with some crystal faces, possibly due to rapid initial growth followed by quenching and some surface pitting from the chemical separation process [50]. The X grain, though, has a smooth texture. Of the SiC grains studied by TEM, mainstream grains are primarily single crystals (most 3C polytype [51]), whereas X-grains are typically polycrystalline [20, 22].

**Fig. 5:**
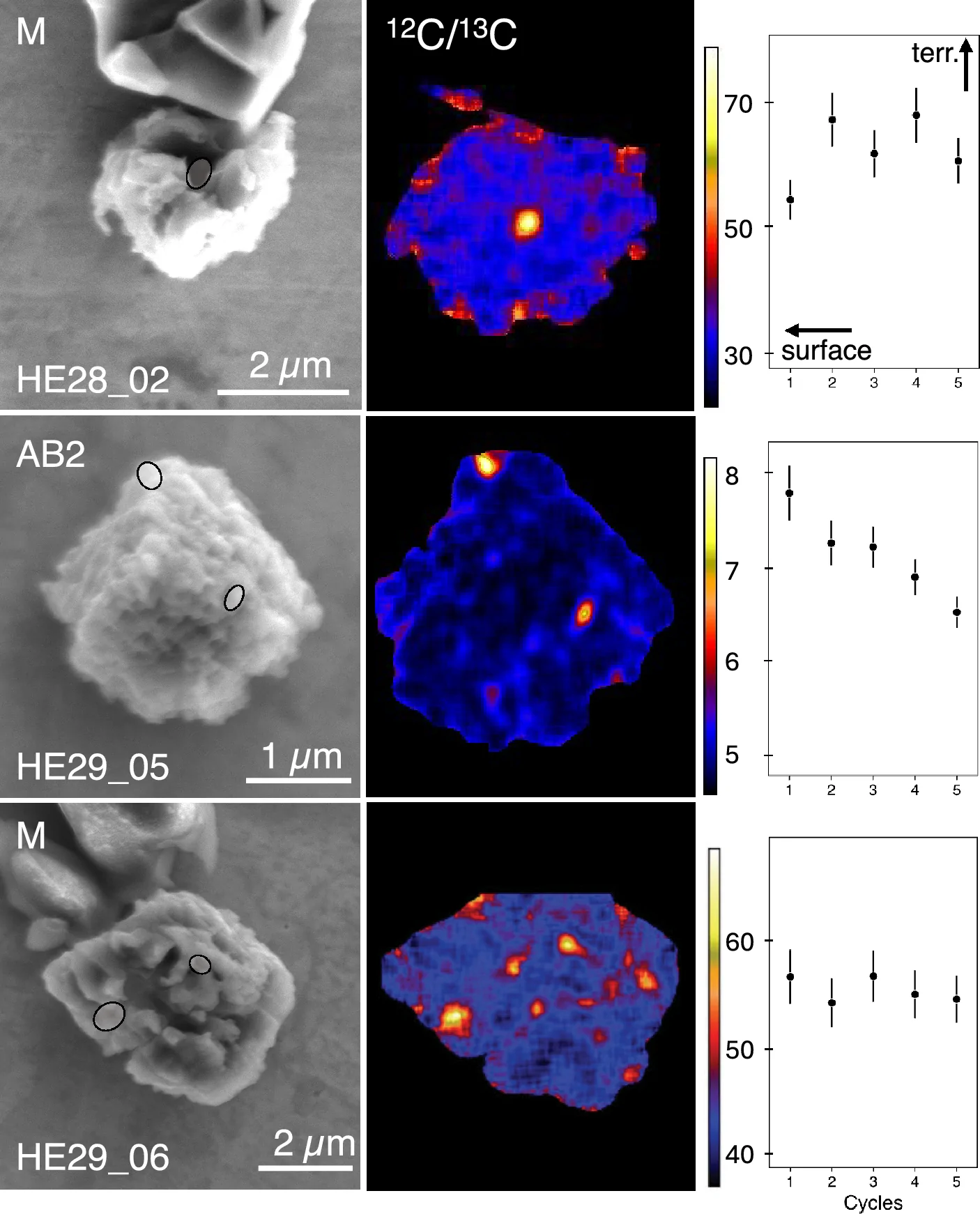
Three presolar SiC grains imaged with SE (left) with respective $^{12}\text {C}/^{13}\text {C}$ images (middle) and $^{12}\text {C}/^{13}\text {C}$ of each of the five cycles (right). The bright yellow areas within the blue host grains are hotspots that are isotopically distinct from the host grains. Approximate hotspot locations are highlighted on the SE images. The 1 sigma cycle data show that the hotspots remain isotopically distinct from terrestrial (terr.) compositions after removal of 30–50 nm of material. The $^{12}\text {C}/^{13}\text {C}$ here is uncorrected; see Fig. 6 for corrected data

In a previous study, presolar SiC grains were isolated without acid etching [50] and are thus considered pristine. In comparison, our grains separated and etched with harsh acids have a higher prevalence of surface pits. However, the crystal faces observed are likely a primary growth feature, because these are also common features in the pristine presolar SiCs [50]. Of the 45 presolar SiC grains, at least 8 show porous features indicating surface pitting that was attributed to acid etching (Fig. 5—samples HE28_02 and HE29_06). The majority of the presolar grains, about 35 of 45, have crystal faces (Fig. 2—sample HE24_01, HE29_01, HE24_08, HE16_01, and HE25_07; Supplemental Document).

The solar composition SiC grains that we call “terrestrial” are morphologically distinct, usually larger with flat faces, conchoidal fractures, and sharper edges. They have a variety of shapes from platy squares to triangular blocks (Supplemental Document).

### Isotopically distinct hotspots

**Fig. 6:**
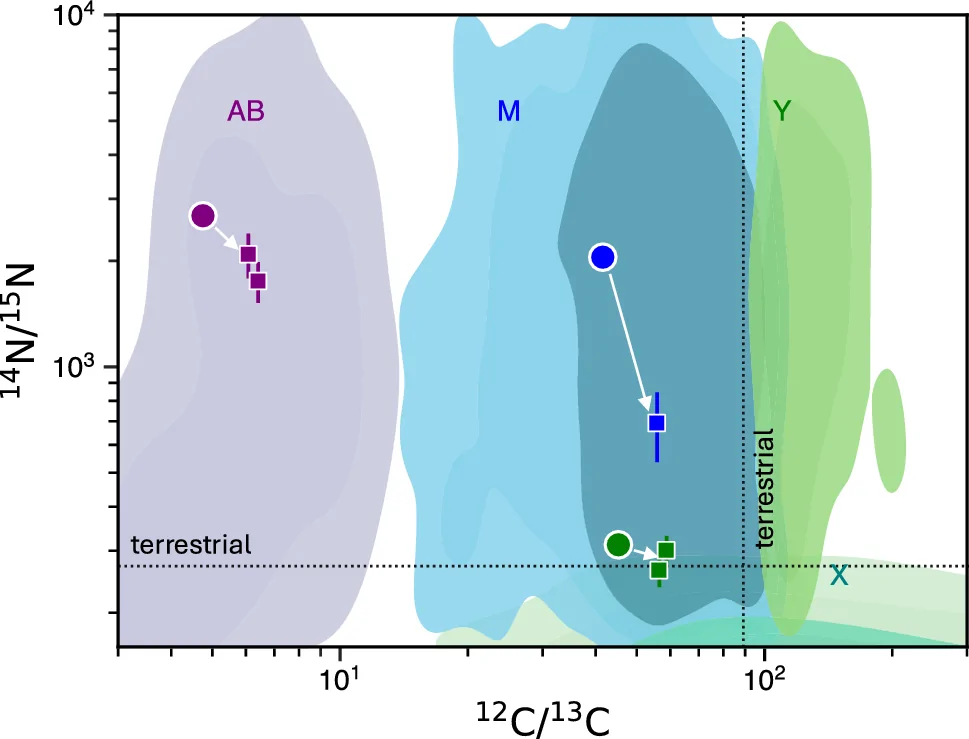
C and N isotope composition of the hotspots, with 1 sigma uncertainties. The colored contour areas, with contours representing 1, 2, and 3σ, show literature data [4] for mainstream (M), AB, Y, and X grains. The host grain compositions are shown as circle symbols with white arrows pointing to the hotspots represented by square symbols. HE29_05 is in purple, HE28_02 is in blue, and HE29_06 is in green

We obtained C, N, and Si isotope ratio images of 17 presolar SiC grains with the NanoSIMS XR prototype. Three presolar grain images show isotopically distinct hotspots with diameters ranging from 150–500 nm, whose $^{12}\text {C}/^{13}\text {C}$ isotopic compositions differ from their respective host grains by 8 to 21 sigma (Fig. 5, Table 1). The hotspots have higher $^{12}\text {C}/^{13}\text {C}$ ratios compared to the host grain, trending towards the $^{12}\text {C}/^{13}\text {C}$ terrestrial value (Table 1, Fig. 6). Most of the hotspots are associated with C/Si ratios higher than that of the host grain; however, it is unclear how much of such elevated C/Si ratios can be attributed to surface topography effects during nanoSIMS measurements. Therefore, the true C/Si ratio of the spots cannot be determined and should not be used for identifying their mineralogy.

Mainstream grain HE28_02 (2.8 µm) has one hotspot, approximately 330 nm × 310 nm, which appears to be located in a cavity of the grain. AB2 grain HE29_05 (2.4 µm) has at least two hotspots, sized at 215 nm × 160 nm and 184 nm × 125 nm, apparently located on the surface, not within cavities. M grain HE29_06 (4.4 µm) also has at least two hotspots, the largest with sizes 356 nm × 237 nm and 570 nm × 485 nm, with at least one located in a cavity. As the 20–25 nm analytical beam is significantly smaller than these hotspots, we expect the sizes reported here are close to the intrinsic size of the hotspots. There are potentially other hotspots, but their isotopic compositions do not significantly deviate from that of the host by >5 sigma. While they may be significant, we choose to focus on only large and highly anomalous hotspots for the purposes of the following discussion.

While some of the hotspots have $^{14}\text {N}/^{15}\text {N}$ ratios closer to terrestrial compared to the composition of their host grain, these were not considered during the interpretation as the N isotope data have larger uncertainties and N isotopic measurements are prone to contamination [52]. The Si isotope ratios of the hotspots are within error (3σ) of those for host grain (Table 1) and, thus, are not considered during interpretation either.

The isotope images in Fig. 5 represent the total counts summed over 5 cycles; however, the data for individual cycles can show whether the isotopic composition of the hotspots varied with depth. The HE28_02 and HE29_06 hotspots did not significantly change in shape or isotope composition across the sequence of 5 cycles (Fig. 5). The HE29_05 hotspots, however, appear to shift away from terrestrial and towards the host grain as a function of depth, though the small number of measurements (n = 5) and relatively large uncertainties preclude drawing a firm conclusion on this point.

## Discussion

### Presolar SiC grains from NWA 7892 in comparison to other meteorites

NWA 7892 is the first CO3 meteorite from which individual presolar grains have been chemically separated. The NWA 7892 presolar SiC grain population found thus far is similar to that of other primitive meteorites. In the following sections, we will discuss how the NWA 7892 presolar SiC grain sizes, isotopic composition, and types compare to those from other meteorite separations.

#### SiC grain sizes and abundance

CO carbonaceous chondrites show chemical and mineralogical similarities to CMs; they are grouped together into the CO-CM clan and are considered to originate from similar nebular materials [53–55]. After recalculation of concentrations using the variable Ne-E(H) approach, the presolar SiC abundance of primitive CMs and CO3.0s is similar, 30 ppm and 10–20 ppm, respectively [3]. In comparison, the singular CO3.2 measured, Kainsaz, had a much lower SiC signature of 0.1 ppm [40]. Alternatively, graphite and silicate presolar grain concentrations are distinct between CMs and CO3.0s: CO3.0s contain much less graphite but can contain presolar silicates, which are typically destroyed by the aqueous alteration experienced by CMs [56].

In a Murchison separation, the most common presolar SiC grain sizes are ∼0.4 µm, with 70% of separated grains ranging from 0.3–0.7 µm [24]. On the other hand, Murchison separated presolar SiC grains >3 µm represent <0.01% of the SiC population [24]. Presolar SiC grains larger than 10 µm have only been identified from one Murchison separation, called the L series separation; 58 grains >10 µm in diameter (ranging up to 66 µm) were extracted from an 88 g piece of Murchison [4, 24]). We do not know if this is the total number of large grains found in the Murchison piece, so we state a lower limit of 0.7 large (>10 µm) presolar SiC grains per gram of Murchison. The L series total SiC concentration was measured to be 2.4 ppm via noble gas analysis.

Although NWA 7892 (CO3) is less aqueously altered and potentially more thermally altered than Murchison (CM2), it contains a significant number of large SiC grains. Of the 45 presolar SiC grains identified in this study, 21 grains are larger than 3 µm, and 5 grains are larger than 10 µm. Additionally, the 13 µm AB grain identified is the second largest AB grain found to date [4, 57]. In total, we estimate that NWA 7892 contains 0.8 large grains per gram (Sect. 3.3). This is a similar abundance of large grains compared to the Murchison L series. In summary, the large grains in NWA 7892 are evidence that this meteorite and the chemical separation procedure allowed preservation of presolar SiC grains, and that NWA 7892 has a similar abundance of presolar SiC grains to Murchison.

#### Isotopic composition

Presolar SiC grains are classified by their C, Si, and N isotopic compositions. Figures 3 and 4 show our isotopic data superimposed on distribution envelopes that define the regions in which several SiC grain types plot. The envelopes define the confidence regions for the various grain classifications, as defined by Stephan et al. [4] based on a survey of literature data. For example, 68% of identified mainstream grains in the literature fall within the darkest blue contour (1σ) on Fig. 3a. Due to our small sample size, we are unable to compare all the type proportions directly; however, our mainstream grains (n = 39) comprise 87% of all grains found so far, which is similar to what has been found in other meteorites.

It has been shown that grains can be contaminated with terrestrial N if they are not sufficiently sputtered, resulting in skewed $^{14}\text {N}/^{15}\text {N}$ results [52]. While approximately half of our grains show slight N contamination, this does not affect our conclusions because partially contaminated values are still many σ away from the terrestrial value (Fig. 4). Overall, NWA 7892 SiC grains are isotopically similar in C, N, and Si to presolar SiC grains from other meteorites.

### Isotopic hotspots in presolar SiC grains

With the high spatial resolution and beam intensity of the NanoSIMS XR prototype, we identified localized, isotopically distinct features in three presolar SiC grains (Fig. 5). Such small-scale isotopic features have not previously been observed. In this section, we investigate the possible origins of these features in detail because if they are instrument-related artifacts, they could compromise future measurements made with this new NanoSIMS.

The features appear as localized hotspots in the carbon isotope images, with diameters ranging from 150 to 500 nm. Their $^{12}\text {C}/^{13}\text {C}$ ratios are higher than those of the host grains, with significance levels exceeding 8σ (Table 1). These elevated ratios indicate that the hotspots have compositions intermediate between those of the host grains and terrestrial composition material (Fig. 6). By terrestrial composition, we mean that the $^{12}\text {C}/^{13}\text {C}$ ratio is around 89; therefore, material with terrestrial composition can be meteoritic. Although the stoichiometry of the hotspots is unclear, the isotopic images indicate that they contain both C and Si. The hotspot origins we discuss include analytical artifacts, contamination, small presolar grains, and subgrains.

It is preferable to conduct nanoSIMS measurements on flat surfaces because topography can produce isotopic offsets, especially near edges or cavities, as analytical artifacts. To evaluate this effect for non-flat presolar SiC grains, we compared $^{12}\text {C}/^{13}\text {C}$ measured on flat regions and edges of SiC standards, where the most extreme geometry-dependent fractionation would be expected. The largest effect was about 5%, similar to what has been found in other studies [58, 59]. The isotopic differences observed between the hotspots and their host grains is much larger, approximately 25 to 35%.

Re-deposition of material during sputtering is another possible artifact. In this scenario, neutral atoms sputtered from the host grain matrix mix with terrestrial material during the analysis to produce an isotopic composition lying along a mixing line between the two. However, reproducing the isotopic composition of the hotspots would require at least 55% (estimated by a simple mixing model) redeposited host-grain material to contribute to a terrestrial-composition contamination (i.e., layer formed while the sample was in the SEM chamber). This is larger than expected from previous work on a flat sample in which only a few percent of redeposited material was observed [60]). However, if the terrestrial material is within a cavity, the effects of redeposition may be greater because sputtering from the (non-flat) cavity walls would have a greater probability of depositing on the material within the cavity. This could continually contribute to a mixed isotopic signal throughout the analysis cycles. One way to test whether this occurred is to measure the hotspot in spot mode rather than imaging mode, so that the cavity walls are not struck by the primary ion beam.

Much work [50, 52, 61–64] has been done to understand and limit the different types of contamination introduced to presolar grains. Contamination is especially difficult to avoid during the chemical separation procedure, even when using high purity chemicals, because grains come in contact with everything that is in the meteorite. One type of contaminant is a thin physical overlay of C-bearing terrestrial composition material (a soil, in the surface chemical context), typically less than a few nm in depth. In fact it is this material that is removed during pre-analysis sputtering. Even though this is much thinner than the 30–50 nm sputter depth achieved during our analysis (estimated from ion dose and sputtering yields), the nanoSIMS primary ion beam would physically mix it with the underlying SiC and could produce an isotopic composition between presolar and terrestrial. Our TRIM [65] calculation results show the maximum mixing depth to be of order 15–20 nm. We observe that the hotspots remain present down to at least 30–50 nm in depth based on depth profiles (5 cycles shown in right panel of Fig. 5). The hotspots on HE28_02 and HE29_06 show no evidence of a change in isotopic composition or size with depth over the 5 analysis cycles. The hotspots on HE29_05 appear to become smaller with depth, which may explain why the isotopic composition appears to trend towards the host grain; however, more cycles are necessary to observe whether the hotspots continue to shrink. Therefore, a surface layer contaminant of terrestrial isotopic composition is not a likely source of the hotspots.

Primary ion beam overlap between the host grain and a terrestrial contaminant is another possible mechanism to explain the hotspots. However, because the NanoSIMS XR prototype resolution is substantially smaller (∼25 nm) than the hotspots (150–500 nm), little beam overlap between the hotspot and host material occurred. This suggests that the hotspots are not purely a terrestrial contaminant. Because IOM can have non-terrestrial isotopic compositions ($^{12}\text {C}/^{13}\text {C}$ is typically 86–88, though some samples range down to 78 [49, 66, 67]), the hotspots are possibly this material, though it would require IOM with larger isotopic anomalies than any that have been previously identified.

With a textured surface like that of a SiC presolar grain, it is possible that the angle of the ion sputtering beam would not clean the grain surface evenly [68] and some contamination could remain. If the analysis beam samples such an area and the cleaned surface at the same time it would produce an isotope ratio intermediate between the clean and uncleaned regions via the redeposition mechanism described above. Given the large diameters of the hotspots compared to the ion beam and the apparent uniformity of the $^{12}\text {C}/^{13}\text {C}$ ratio across the larger spots, such sampling seems unlikely. Furthermore, the isotopic composition of the spots would change over time due to the relative contribution of the contaminant decreasing as it continues to be sputtered away. As discussed above, hotspots in one presolar SiC grain may show evidence of reducing in size over depth; therefore, more work is needed to investigate the possibility of uneven sputtering.

The hotspots could be explained as surface reaction products, created during the chemical processing of SiC grains or while the SiC grains were within the meteorite parent body. Silicon carbide reactions have been observed under higher temperature conditions in the presence of water and/or organic acids. Hydrolyzing and/or hydrothermal reactions on SiC producing silica and glassy carbon have been observed at $>600~^\circ$C and >100 MPa [69] as well as down to 250 $^\circ$C and 10 MPa [70]. In addition to temperature and pressure, other factors that can influence these reactions include pH, composition, and concentration [69]. A C-rich product with the isotopic composition of the observed hotspots would only form if C-rich material with terrestrial isotopic composition was involved in the reaction, whether that be IOM or another phase within the meteorite. The required temperature and pressure conditions for this kind of reaction were not reached during the separation procedure. While primitive CO3 parent bodies primarily experienced only low metamorphic temperatures ($\sim 200~^\circ$C and up to 1.5 MPa [71]), temperature signatures of up to 550 $^\circ$C are present within a portion of some meteorites [40]).

Other than analytical artifacts or contamination, an alternative source for the hotspot features is presolar in origin. During chemical separation, a small SiC grain could be attached to the surface of a larger grain or lodged within a cavity. However, because the AB2 grain HE29_05 would be hosting a small AB2-composition SiC grain, this scenario is statistically unlikely given the low abundance of AB grains (4.8% [4]). The hotspots could also represent subgrains related to the formation of the host grain in the same parent star. In this case, it would need to be proven whether a smaller grain could become attached to a larger one in the dusty region surrounding an AGB star after grain condensation as material from successive pulses mixes.

These observations demonstrate that the NanoSIMS XR prototype can resolve isotopic heterogeneity within presolar SiC grains at length scales of less than 100 nm, which is much smaller than previous nanoSIMS versions. Despite extensive consideration of their possible origins, the nature of the hotspots remains unclear. The hotspots may be artifacts generated by sputter-induced mixing of the host grain and a contaminant material. However, chemical reactions, either during laboratory separation or on the host body, and the agglomeration of small grains onto larger ones in the circumstellar envelope remain viable possibilities as well. The continued study of these and other presolar grains with the NanoSIMS XR prototype, along with the identification of additional hotspots or other isotopically distinct features, the determination of their mineralogy, and the establishment of whether they are confined to the surface or located within the grain interiors, will be important next steps.

## Summary and outlook

We performed a presolar grain separation following Amari et al. [24] and Korsmeyer [34], to isolate nanodiamond, SiC, graphite, and oxide presolar grains from a 24.66 g aliquot of the primitive CO3 meteorite, NWA 7892. This is the first time individual presolar grains have been chemically isolated from a CO3 meteorite. In this study, we have focused on characterizing SiC grains from the HE size fraction (>3 µm).

We measured 65 SiC grains for their major element isotopic composition with the NanoSIMS 50 (C, Si, n = 35) and the NanoSIMS XR prototype (C, Si, N, n = 30) at LLNL. We have identified 45 presolar grains: 39 Mainstream grains, four AB grains, one Y grain, and one X grain. The remaining measured grains have distinct platy morphologies with close-to-terrestrial C isotopic compositions; therefore, we infer that they are contamination introduced during meteorite processing. The isotope compositions of the 45 presolar SiC CO3-sourced grains match those of previously studied grains, which were found primarily in the CM2.5 meteorite Murchison [4]. Several large (>10 µm) SiC grains are found from the NWA 7892 separation, similar to the number found from Murchison separations, suggesting that NWA 7892 is primitive enough to allow the survival of large presolar SiC grains.

Three presolar SiC grains (2–3 µm in diameter) measured with the high spatial resolution NanoSIMS XR prototype, contain isotopically distinct hotspots (150–500 nm in diameter), that are clearly visible in carbon isotope images. The hotspots have $^{12}\text {C}/^{13}\text {C}$ ratios closer to terrestrial than their host grains. Such small-scale isotopic variations have not been observed before due to limitations imposed by the previous generation of instruments, and their origins are difficult to determine. We consider several possible explanations, particularly because instrument-related artifacts could affect future measurements made with the new NanoSIMS. Viable possibilities include redeposition during beam sputtering of an uneven surface, chemical reaction products formed during parent body metasomatism, and subgrains formed in the same star as the host grain. Most importantly, the observation of isotopically distinct features approximately 100 nm in size demonstrates the potential of the new NanoSIMS XR prototype and its LoTIS source for measuring small-scale isotopic variations.

## Supplementary Information

Below is the link to the electronic supplementary material.

Supplementary file 1 (pdf 33807 KB)

## Data Availability

This manuscript has data included as electronic supplementary material. [Author’s comment: Most data generated or analyzed during this study are included in the published article and its supplementary information files.]

## Code availability statement

Code/software will be made available on reasonable request. [Author’s comment: The code/software generated during and/or analyzed during the current study is available from the corresponding author on reasonable request.]
