# Supplementary material for: Presolar silicon carbide grains from a CO3 meteorite
Source: Eur Phys J A Hadron Nucl. 2026 Oct 4;62(10):203. doi: 10.1140/epja/s10050-026-01959-5 (PMC13635035; doi:10.1140/epja/s10050-026-01959-5)
Supplement: Supplementary file 1 [file 10050_2026_1959_MOESM1_ESM.pdf]

Dunham et al. 2026

Supplemental Document

- Presolar SiC images show in same order as Table 1, labeled by type.
- SEM image of NanoSIMS XR prototype individual fixed beam ~25 nm craters
- Isotopically anomalous spot images at the end of the document.

AB

CO_HE08_07

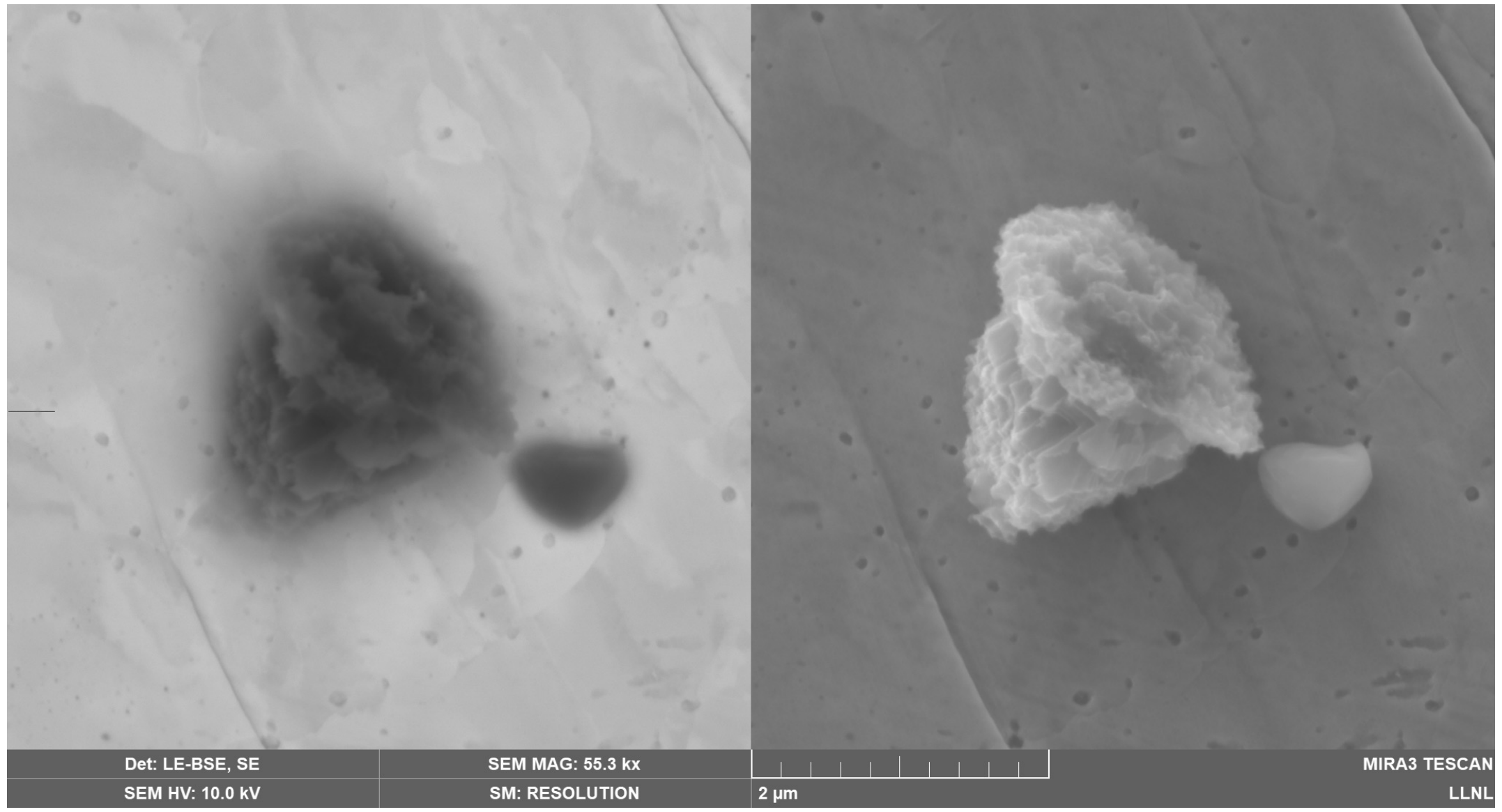

CO_HE24_08

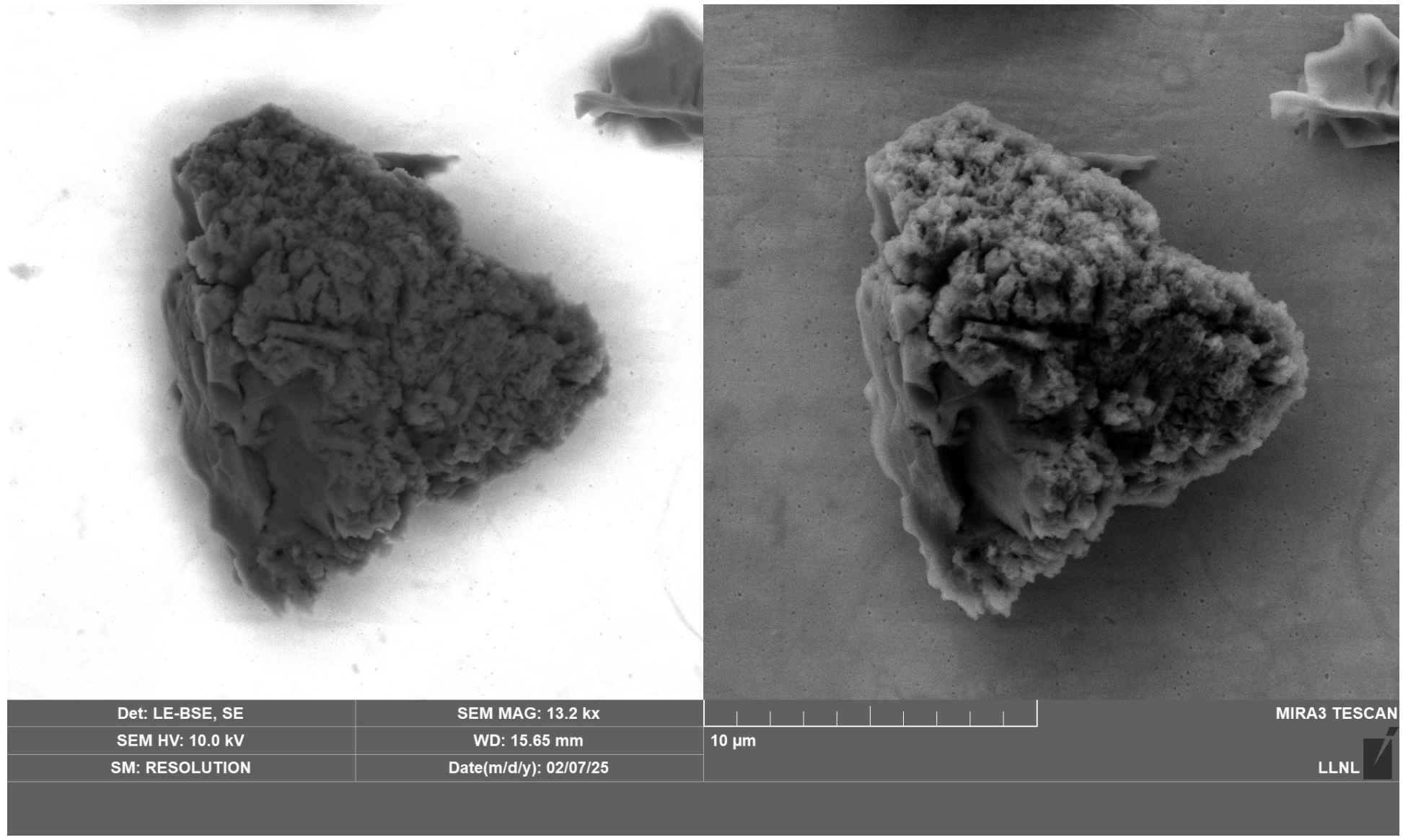

CO_HE08_11

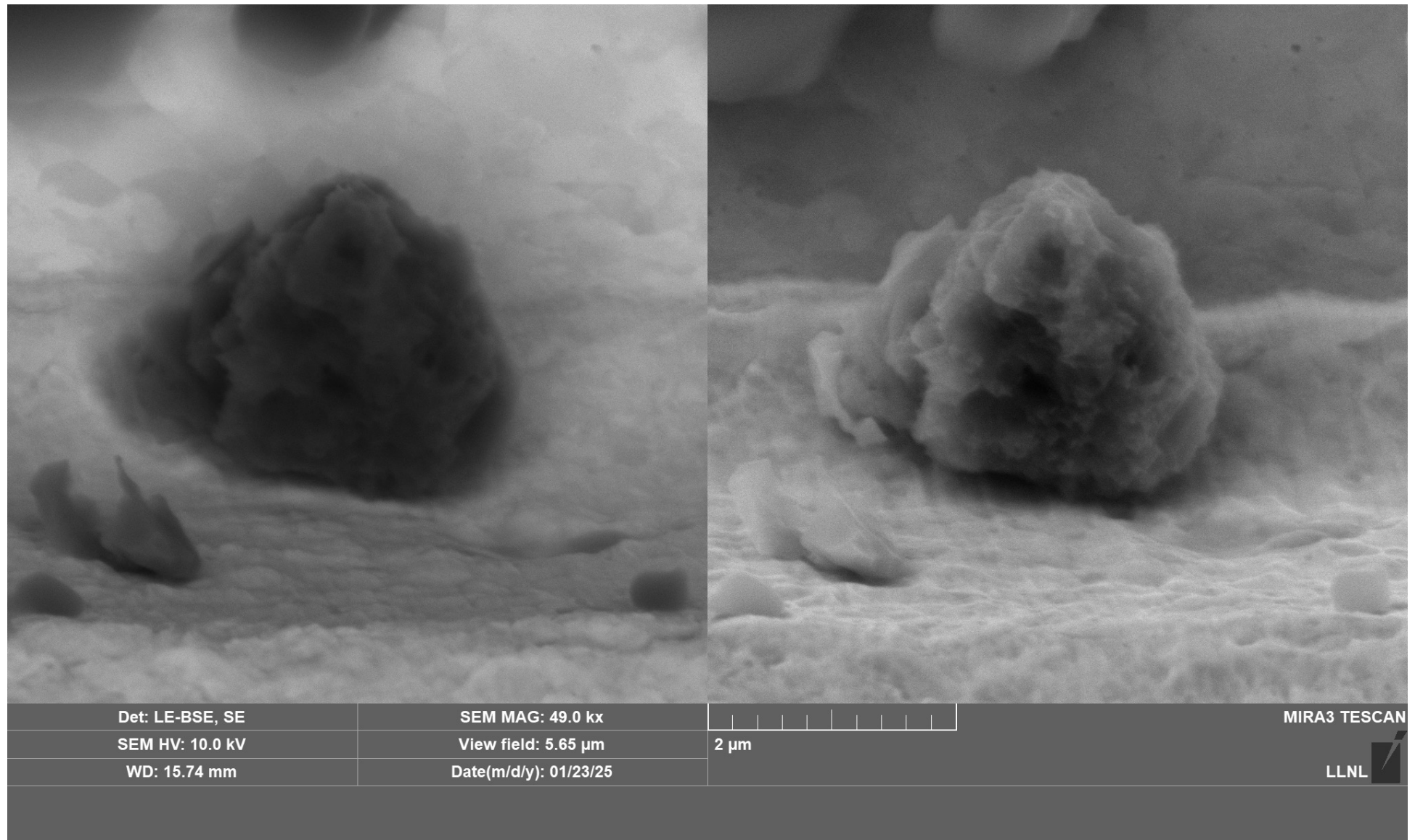

CO_HE29_05

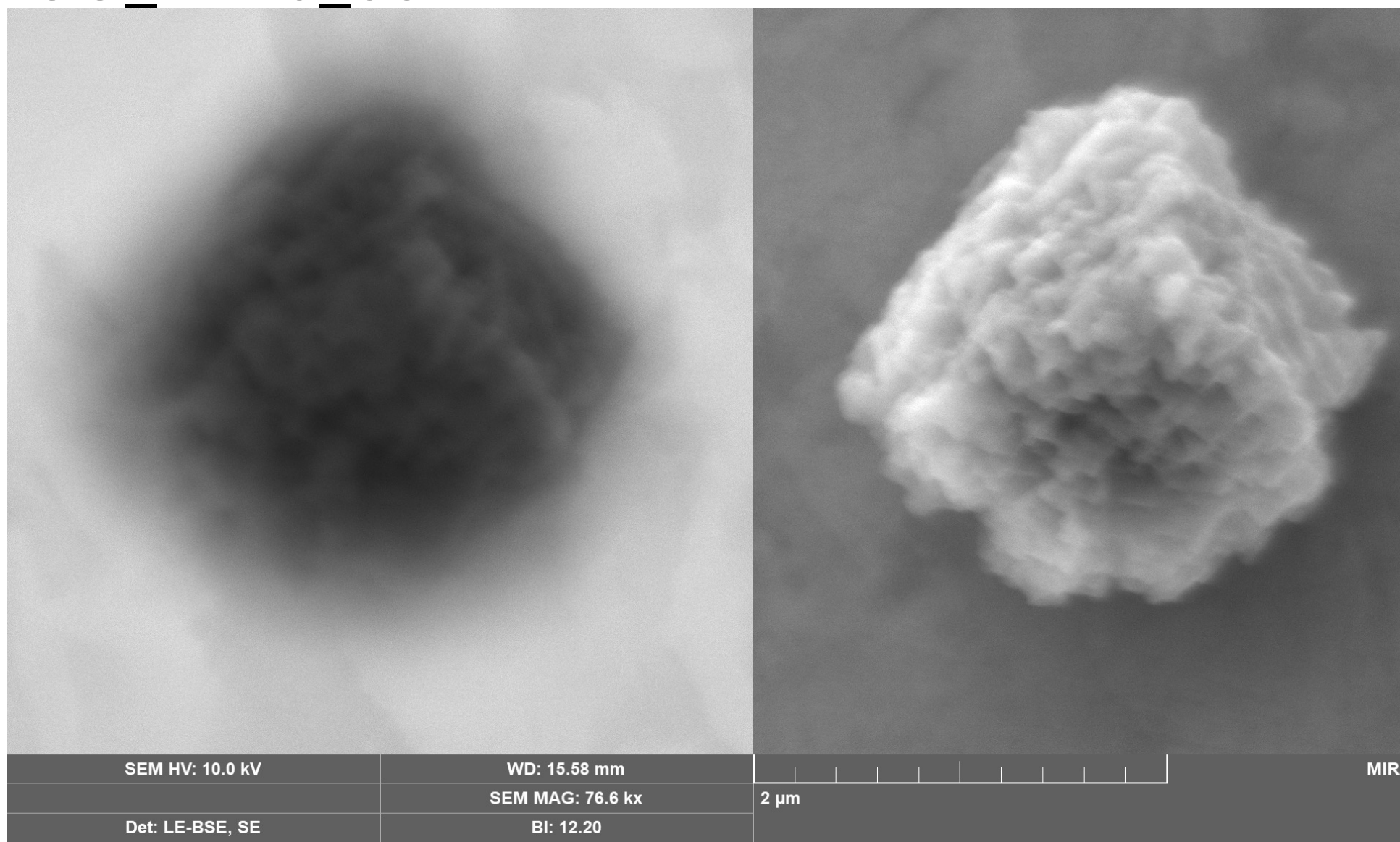

M

CO_GA07_01

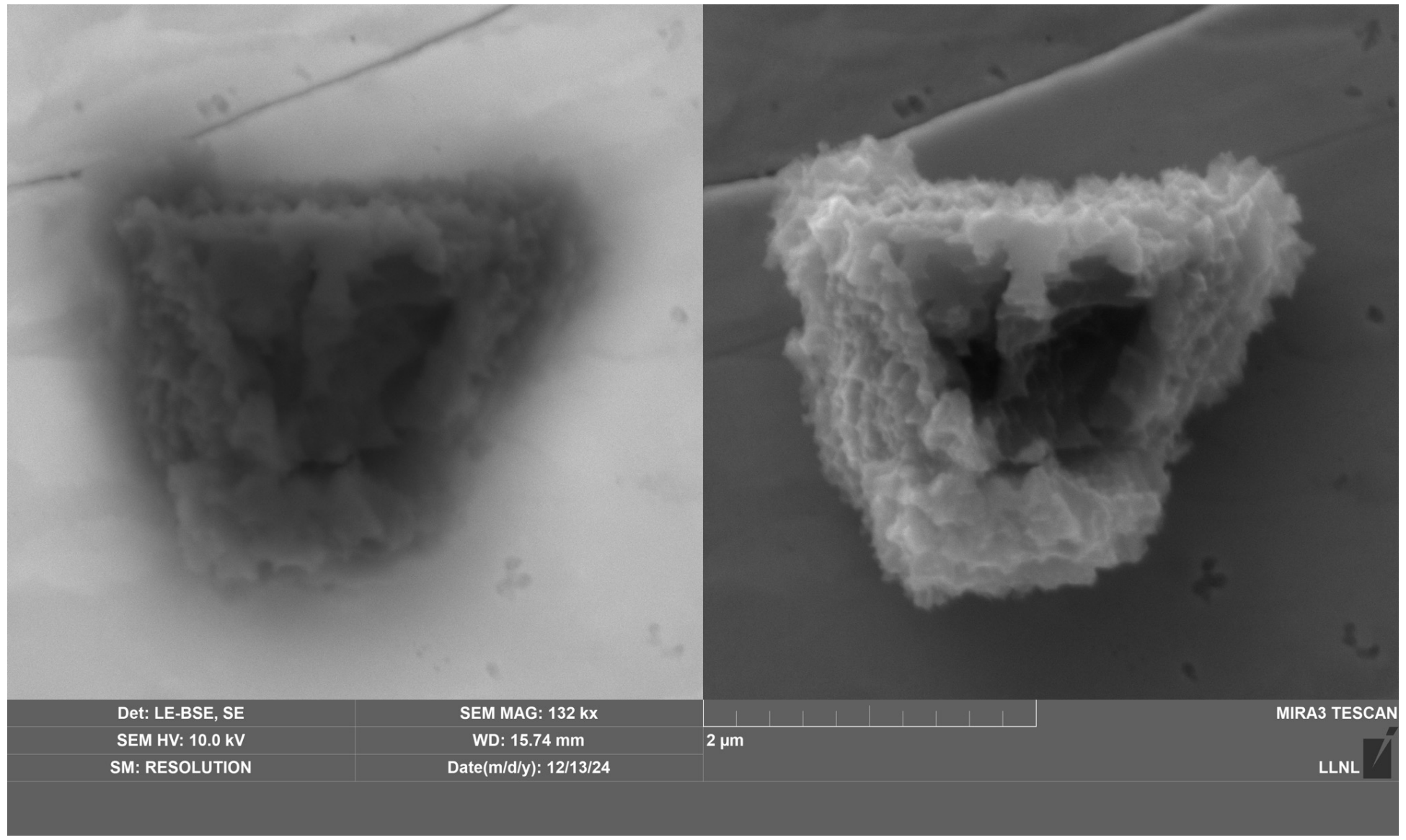

CO_GA07_12

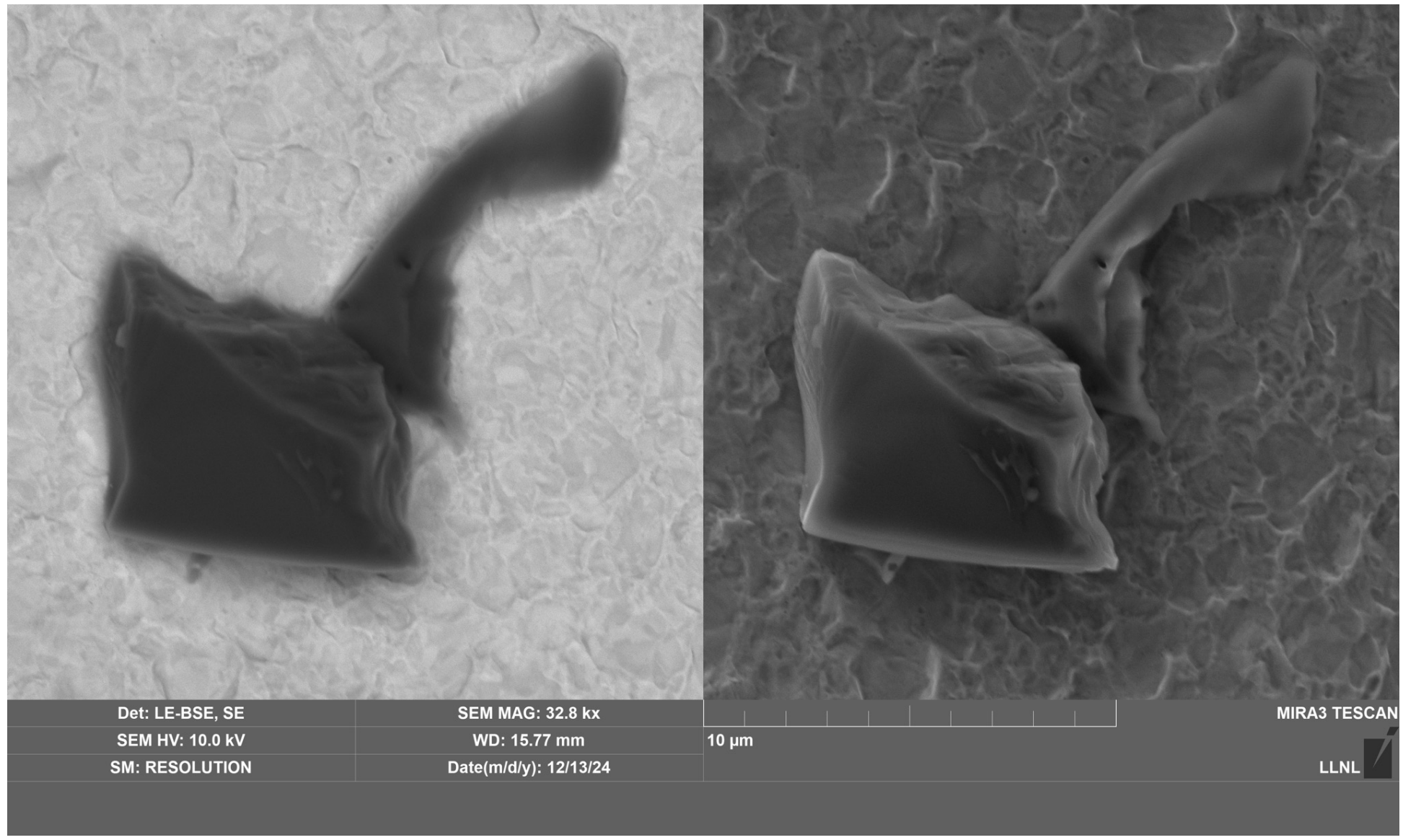

CO_GA07_09

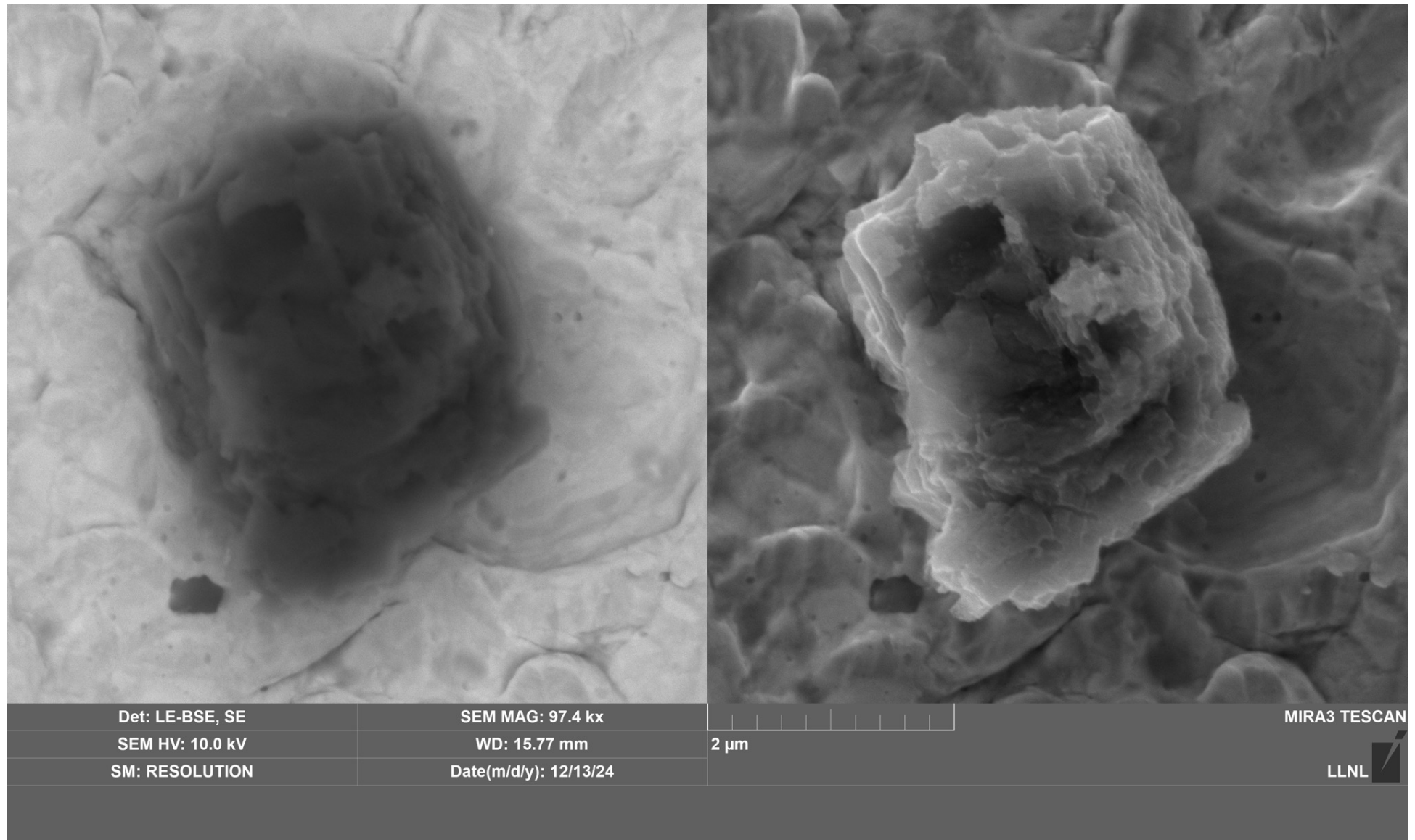

CO_HE08_04

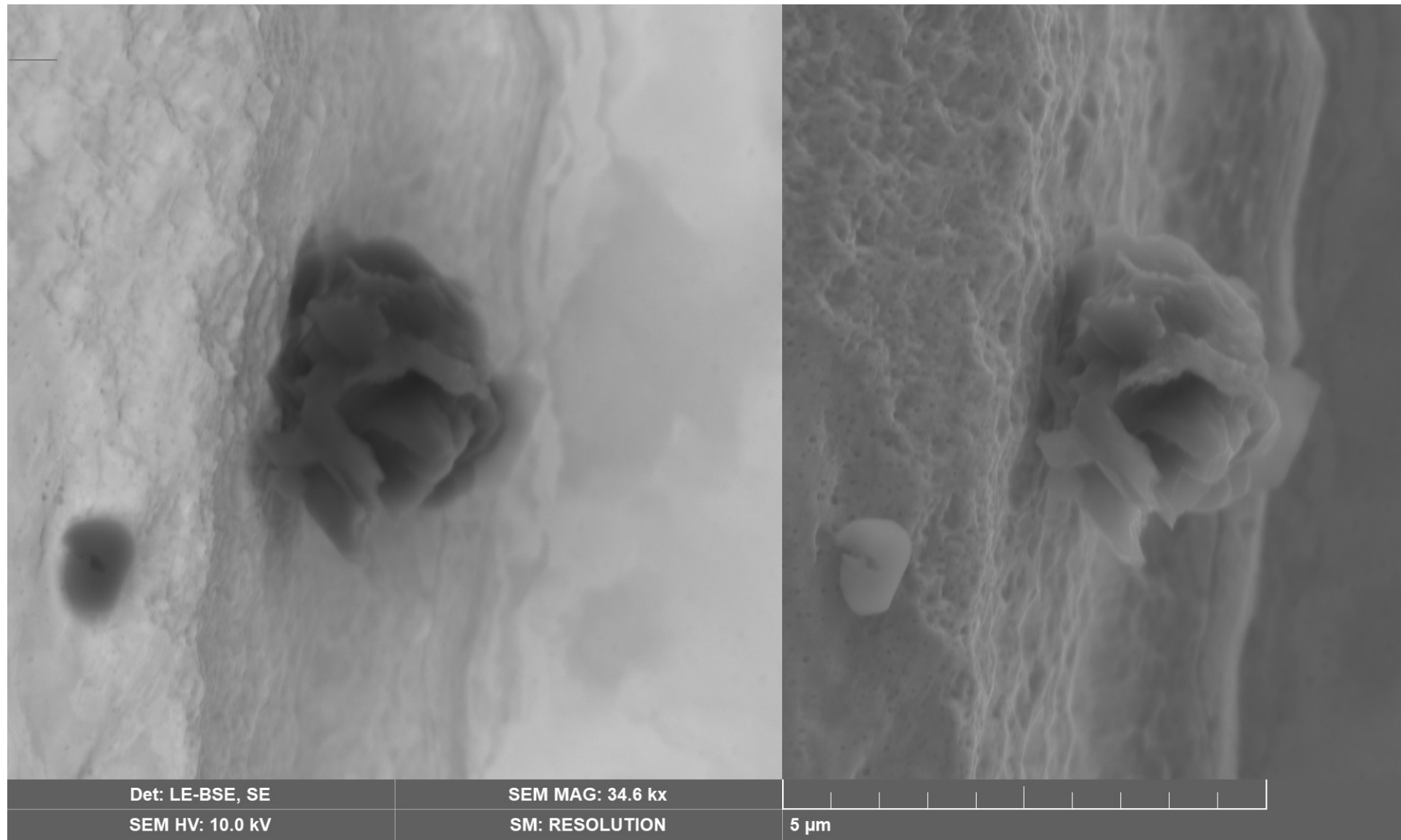

M

CO_HE08_06

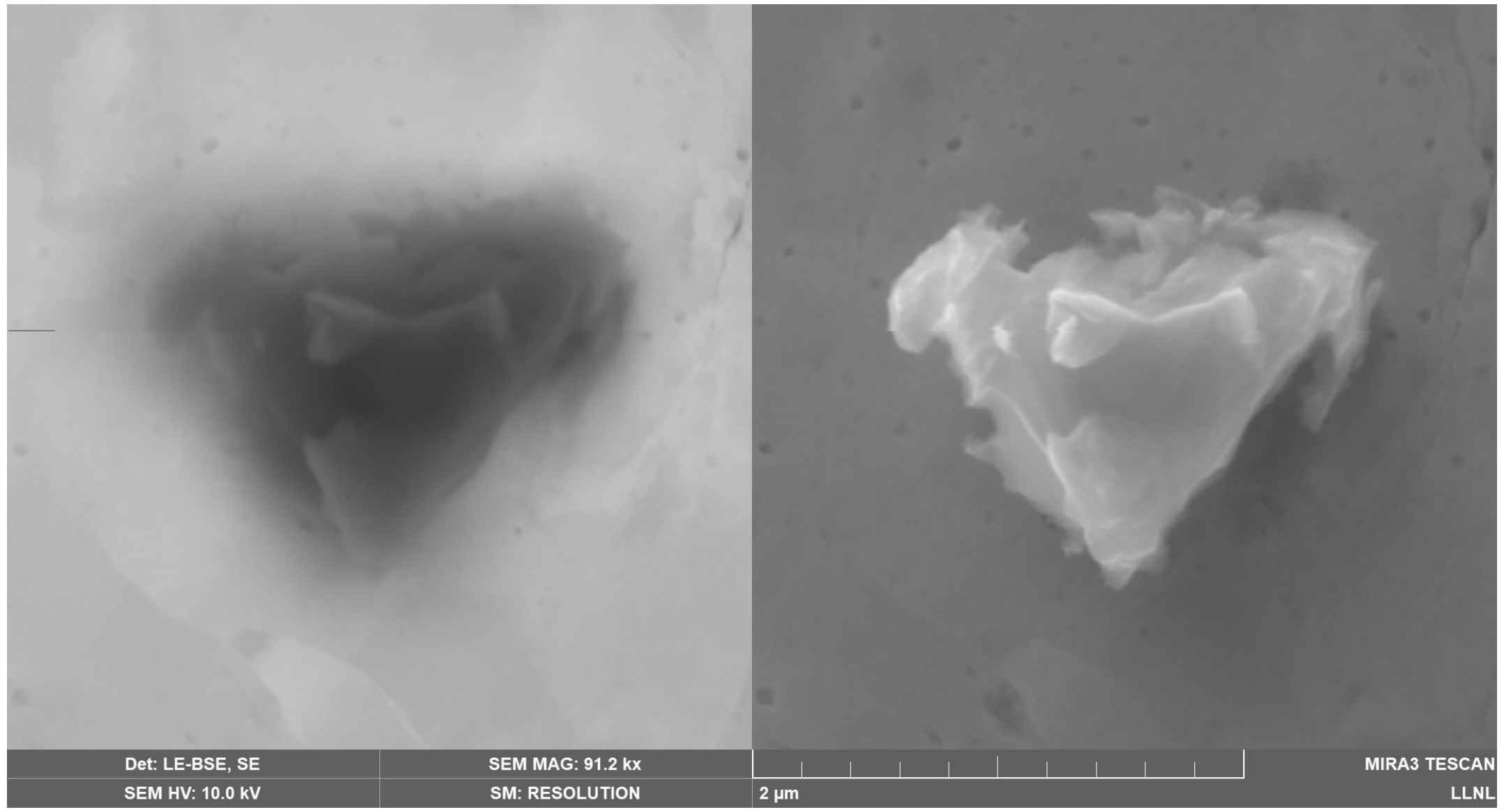

CO_HE09_06

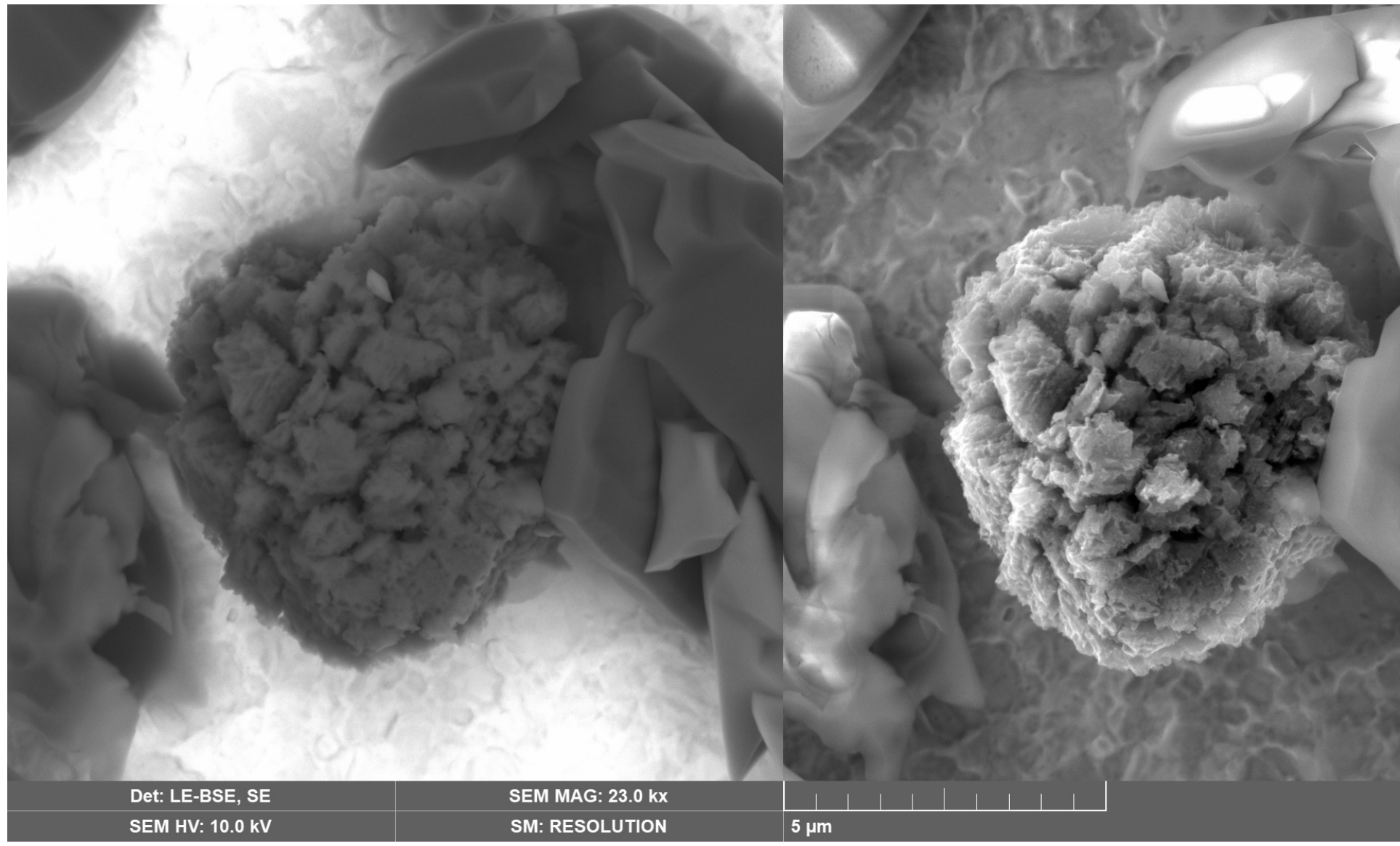

CO_HE09_05

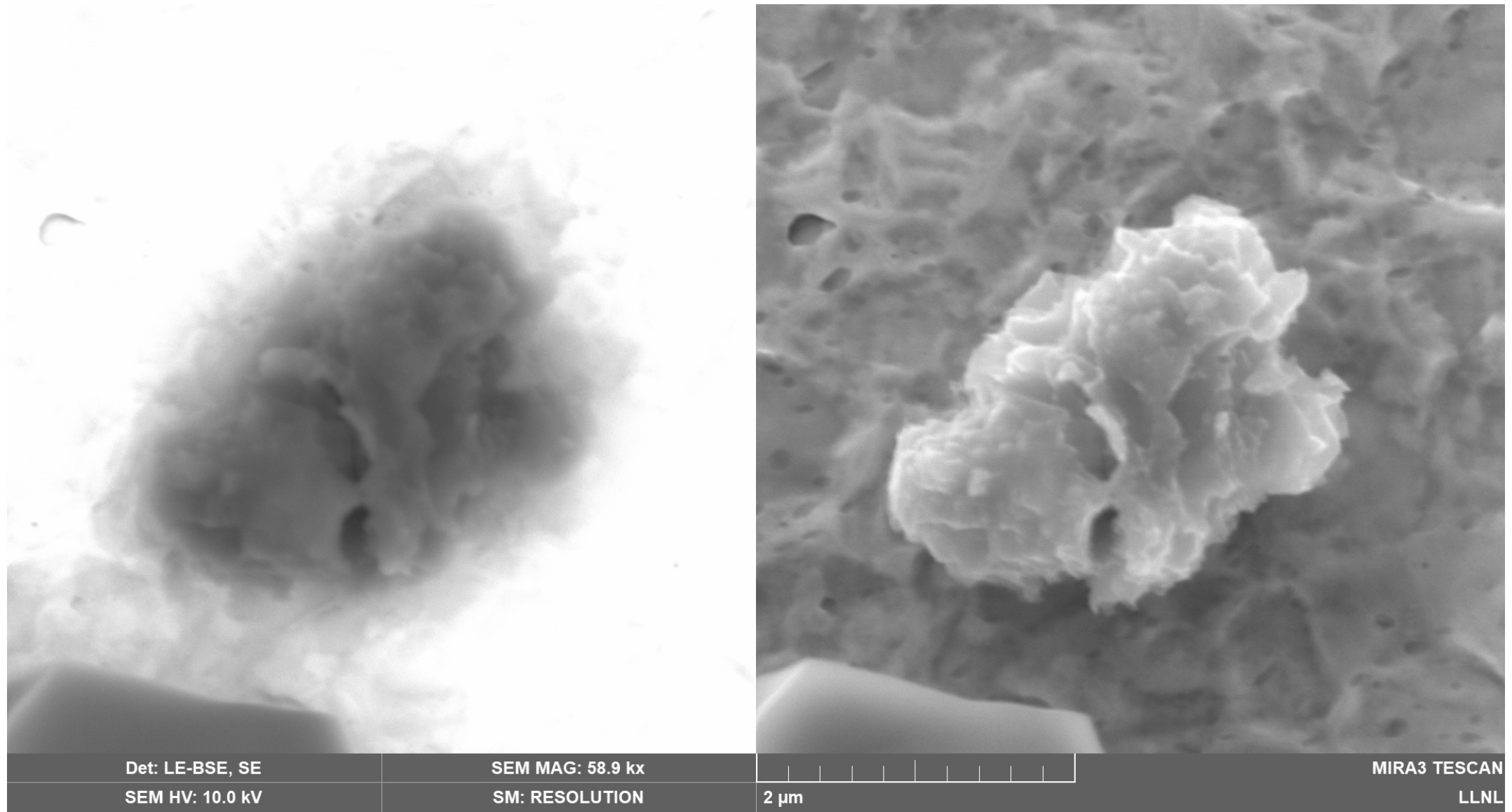

CO_HE14_04

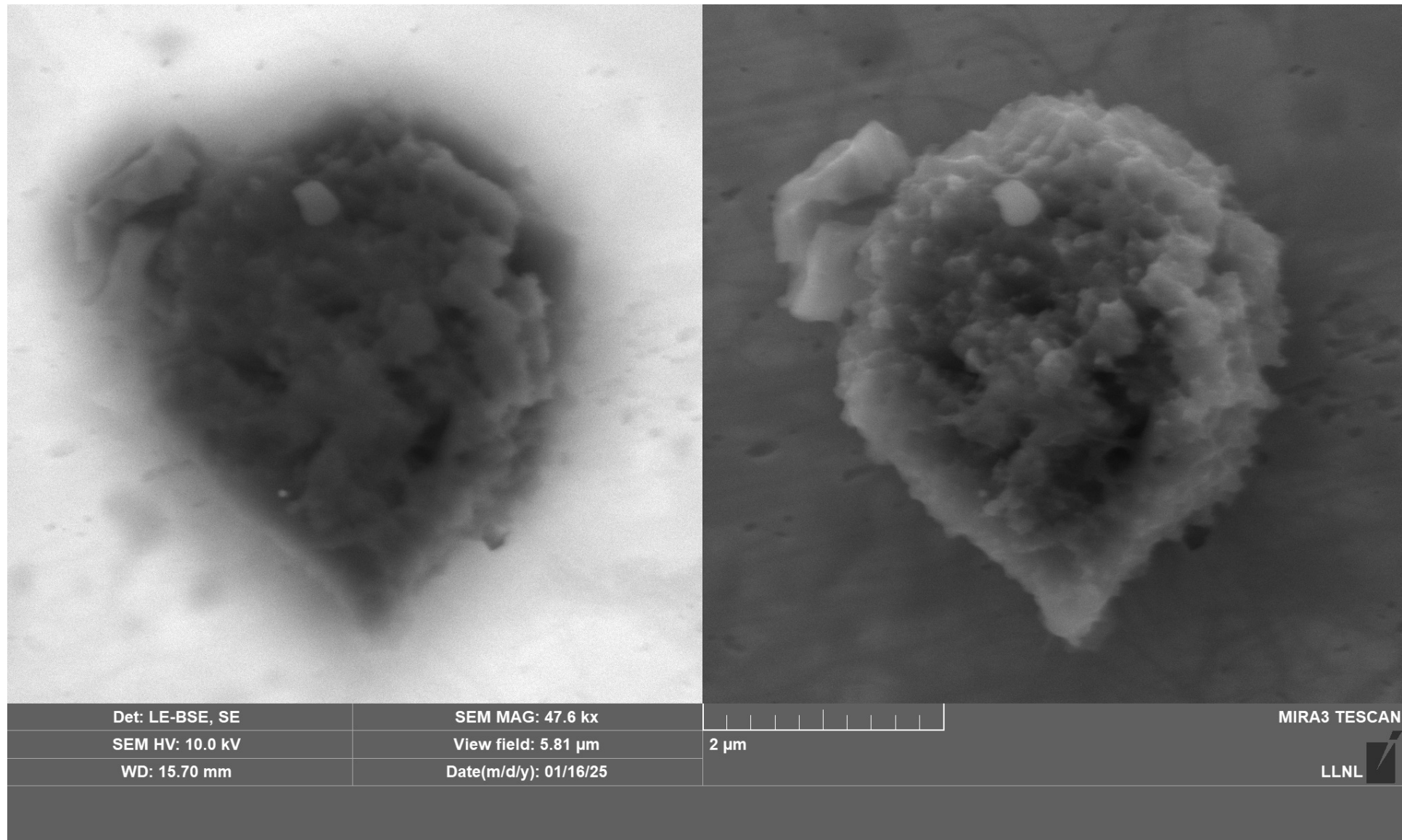

M

CO_HE16_01

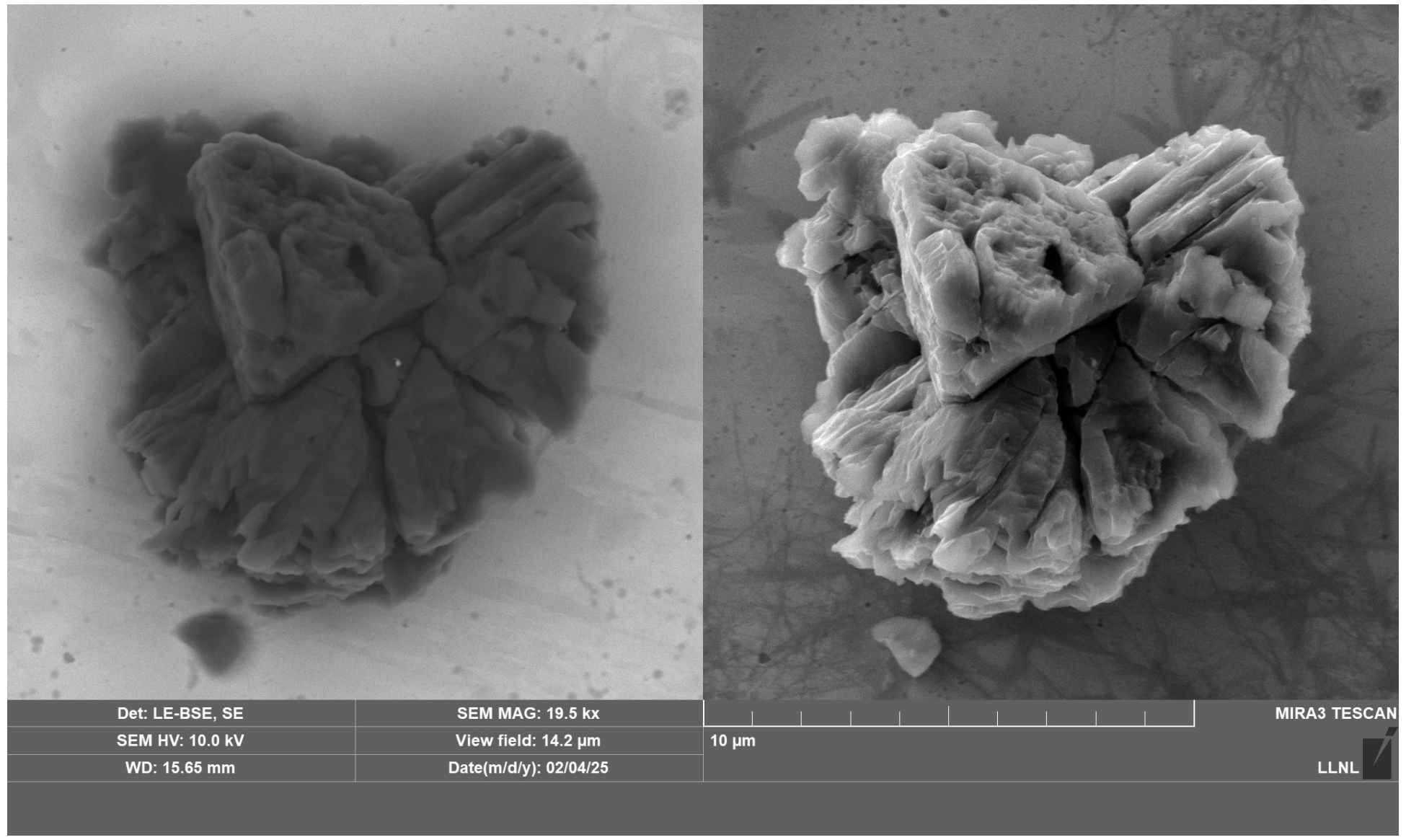

CO_HE18_04

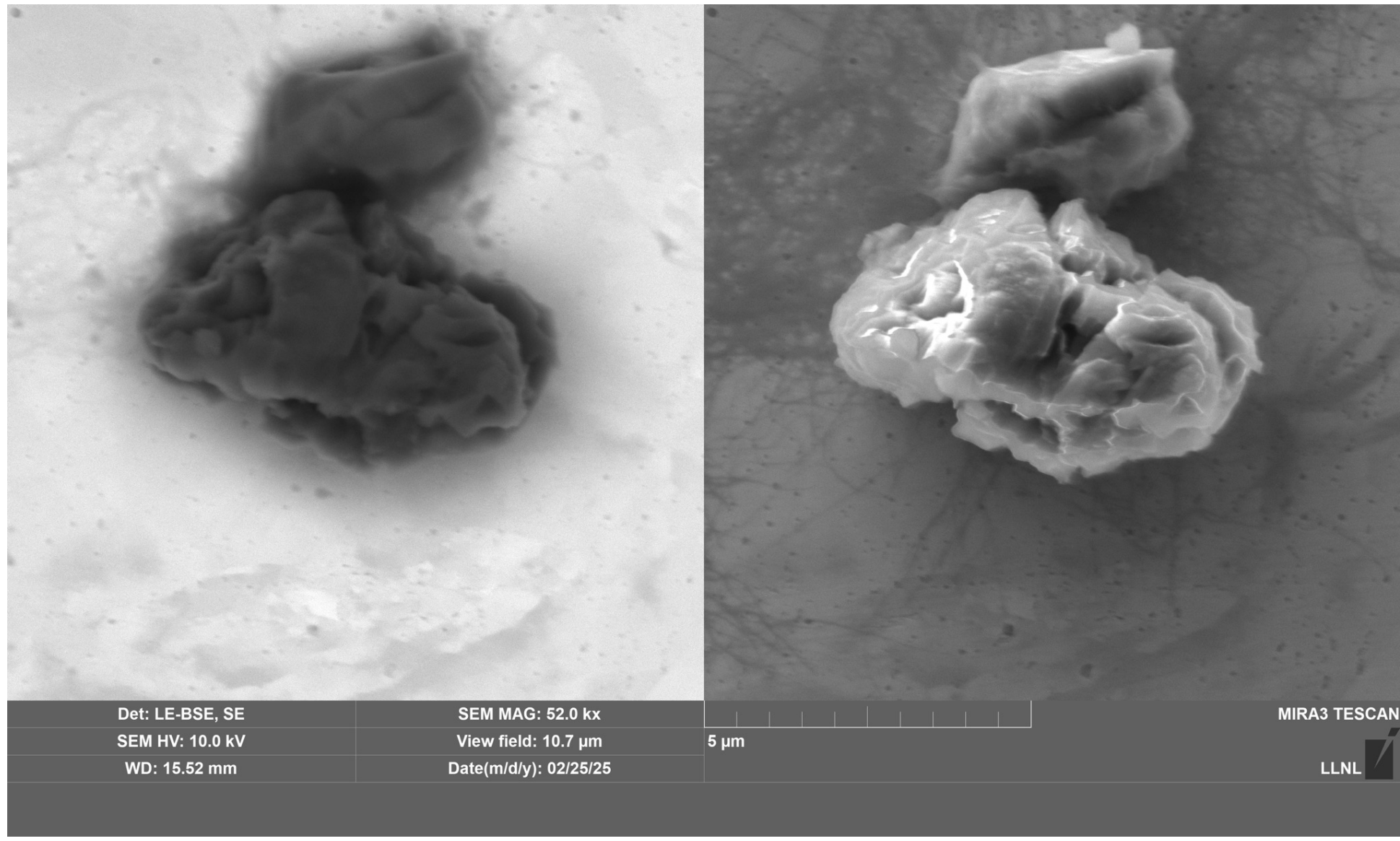

CO_ HE18_03

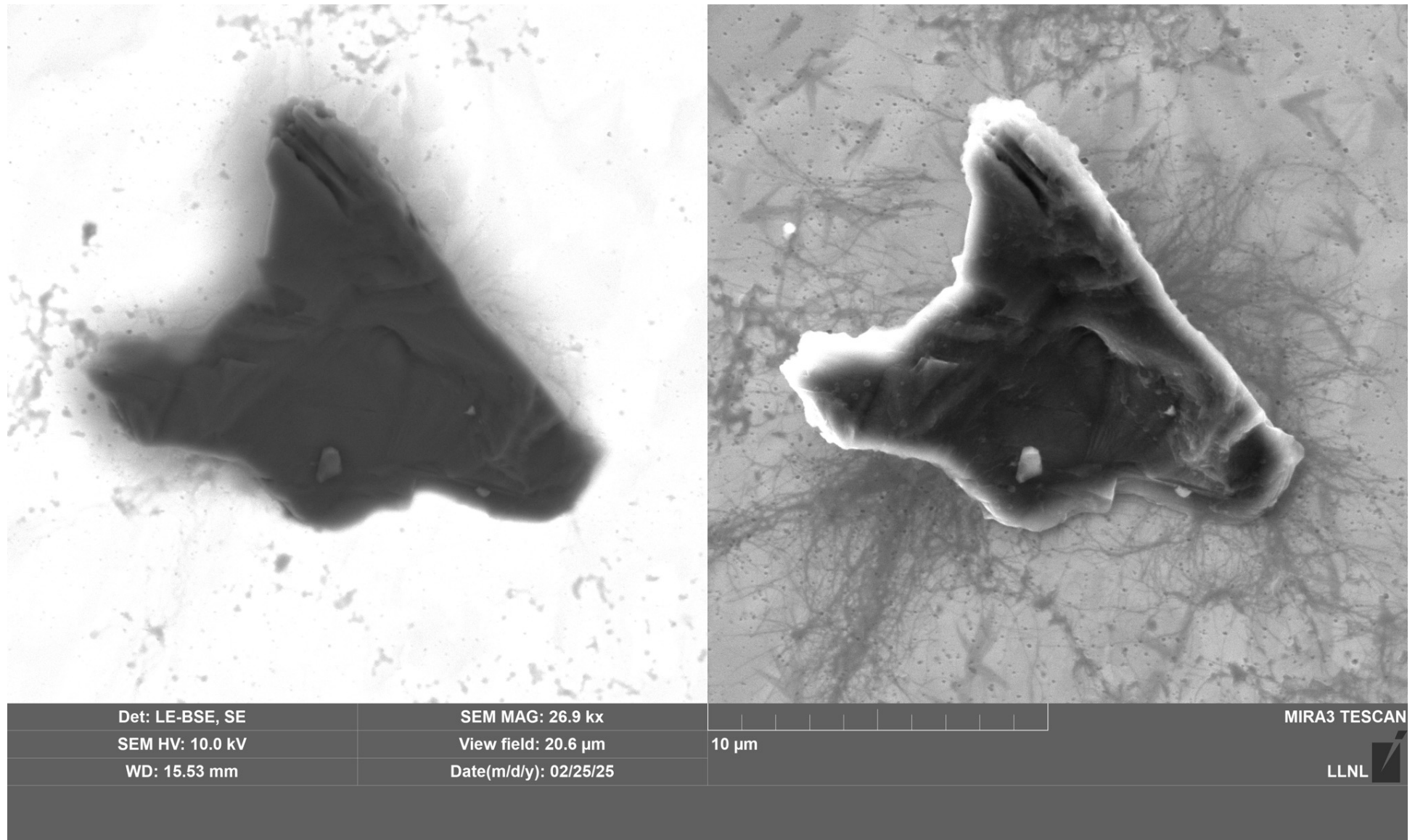

CO_ HE19_02

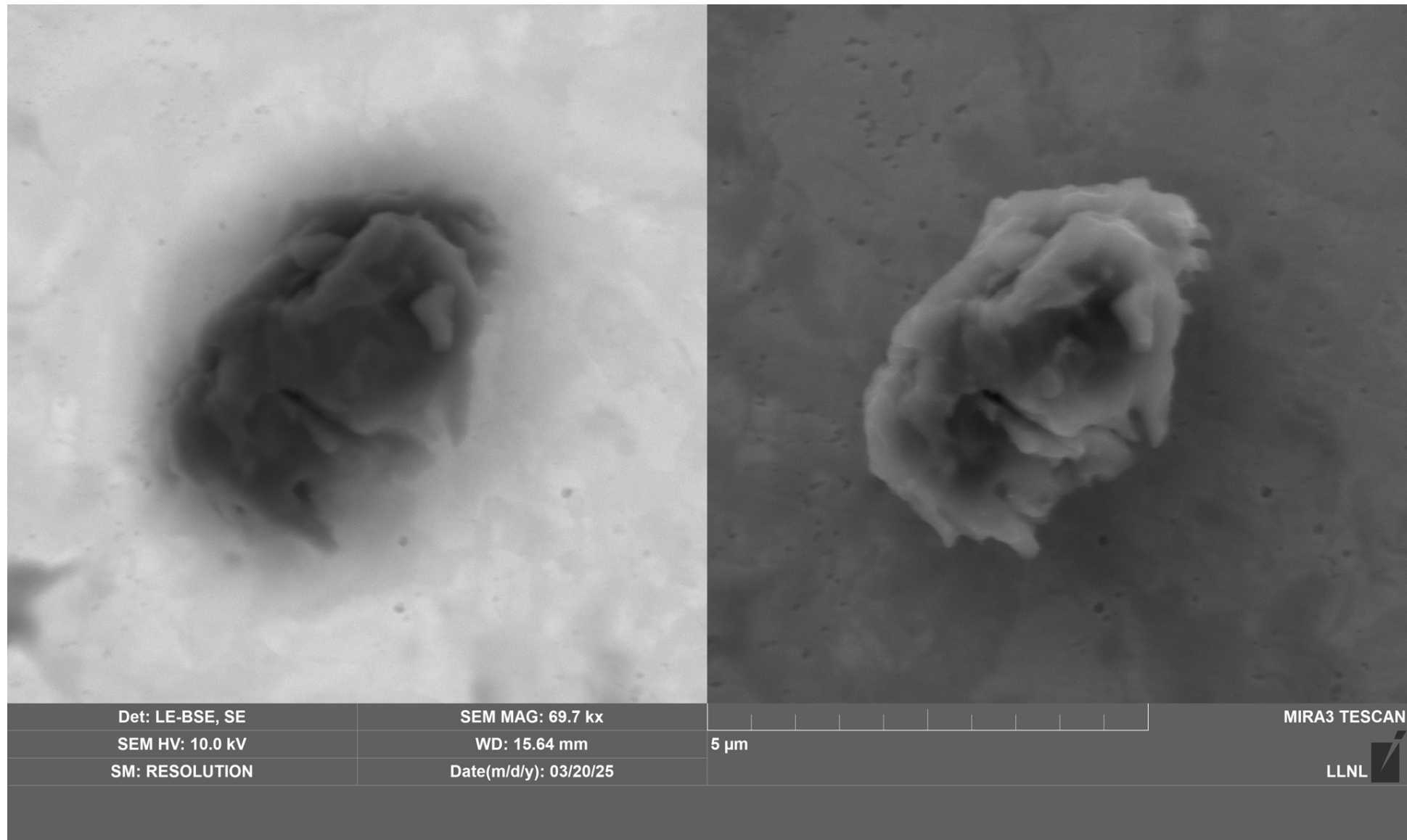

M

CO_ HE20_01

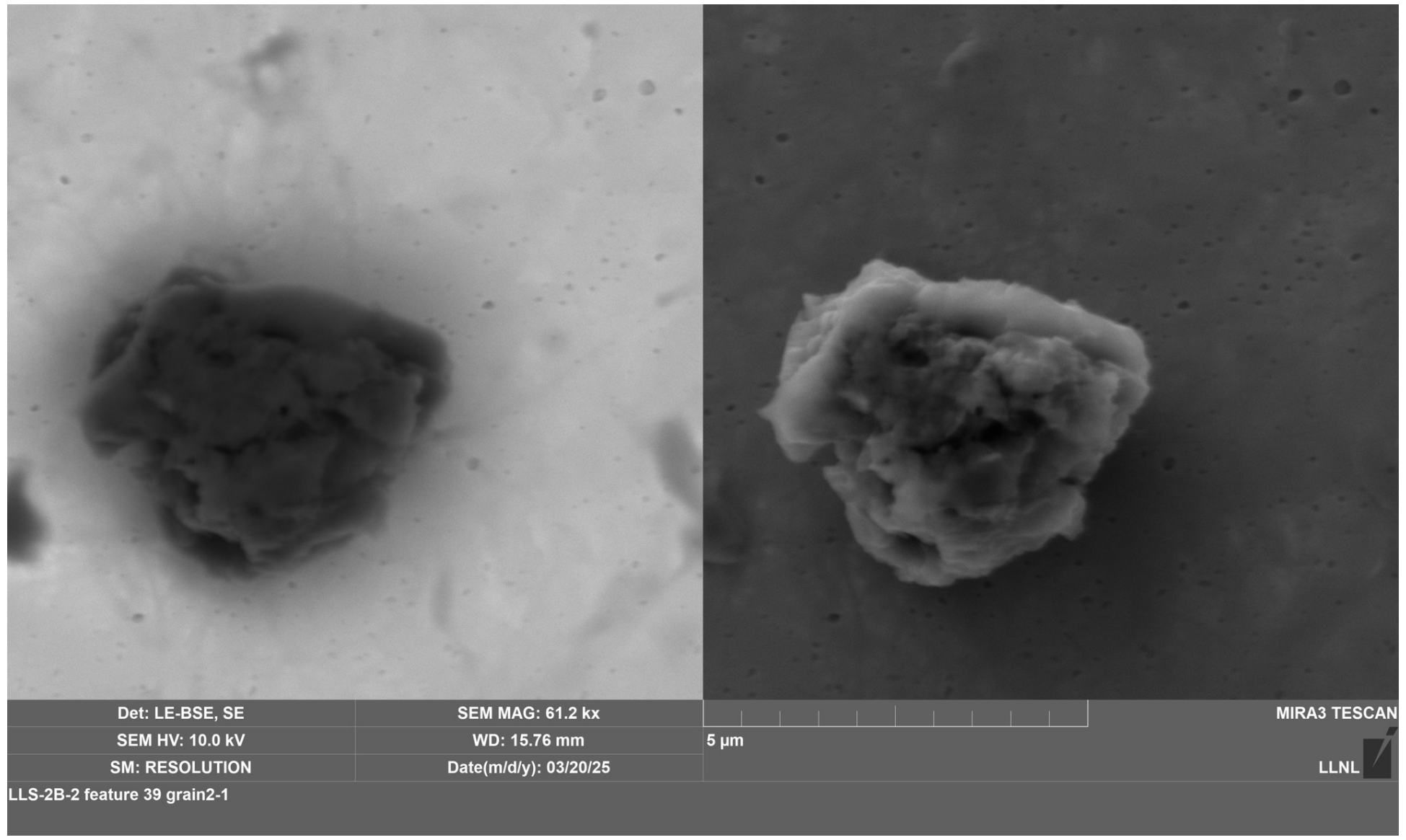

CO_HE23_04

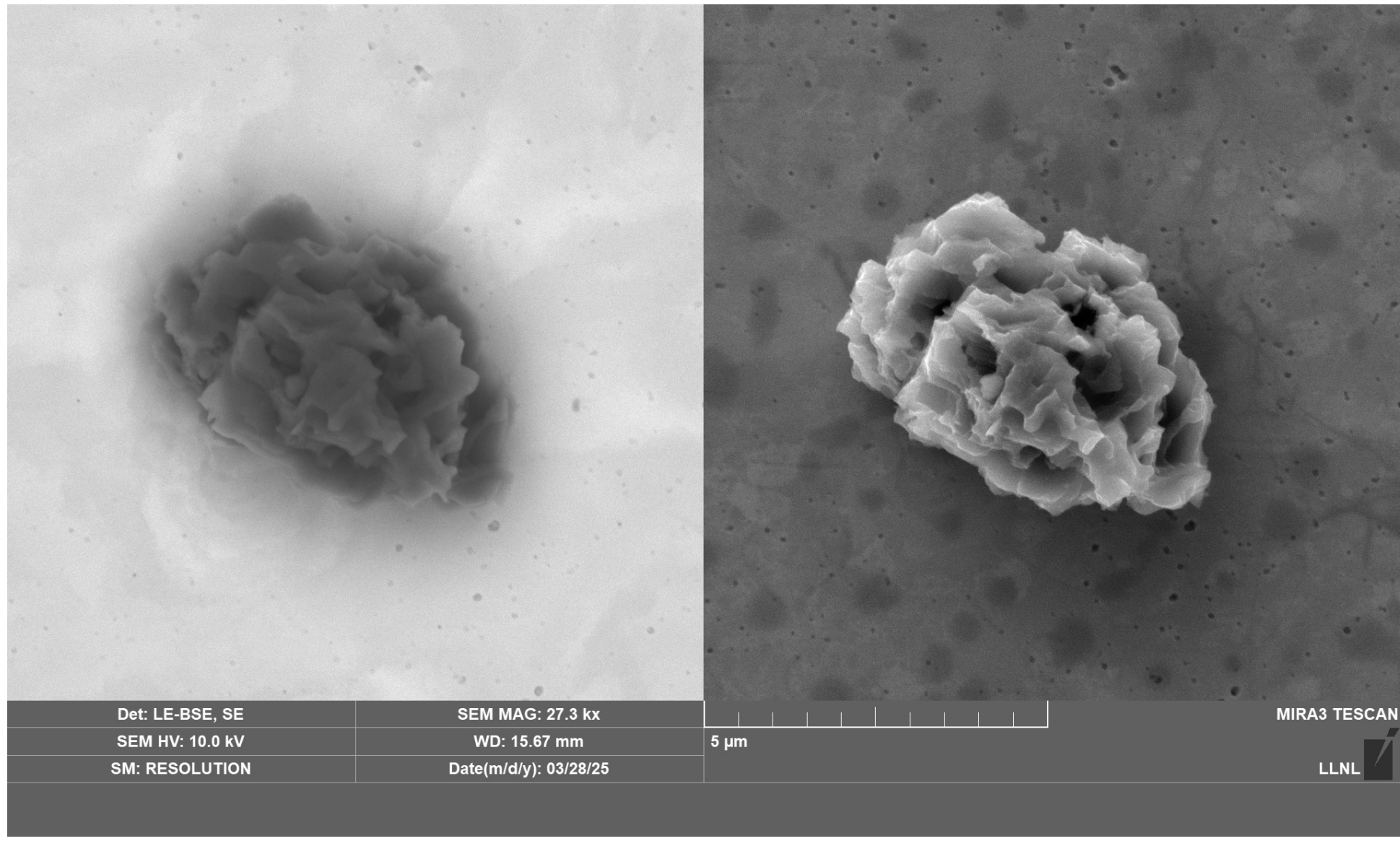

CO_ HE23_03

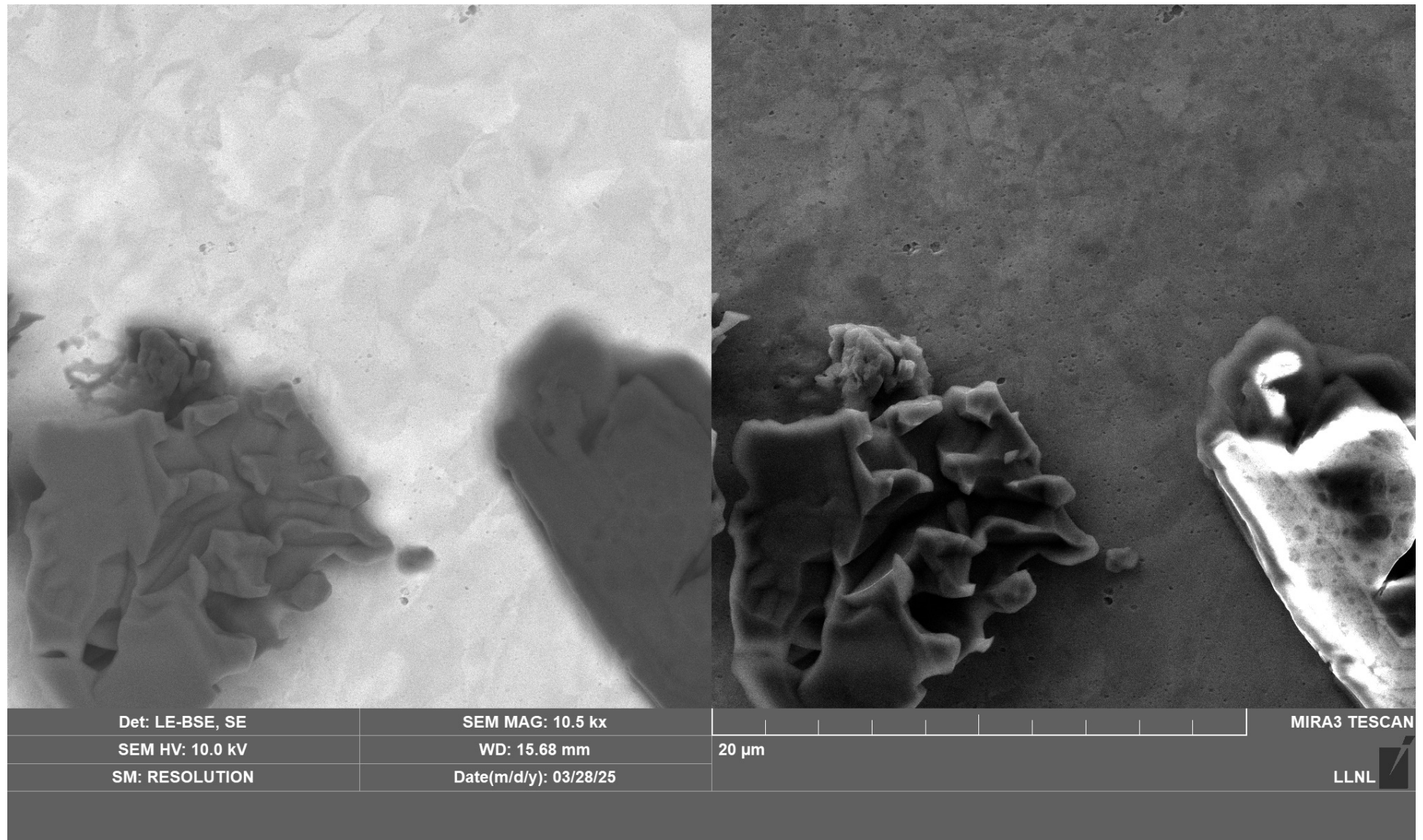

CO_ HE23_06

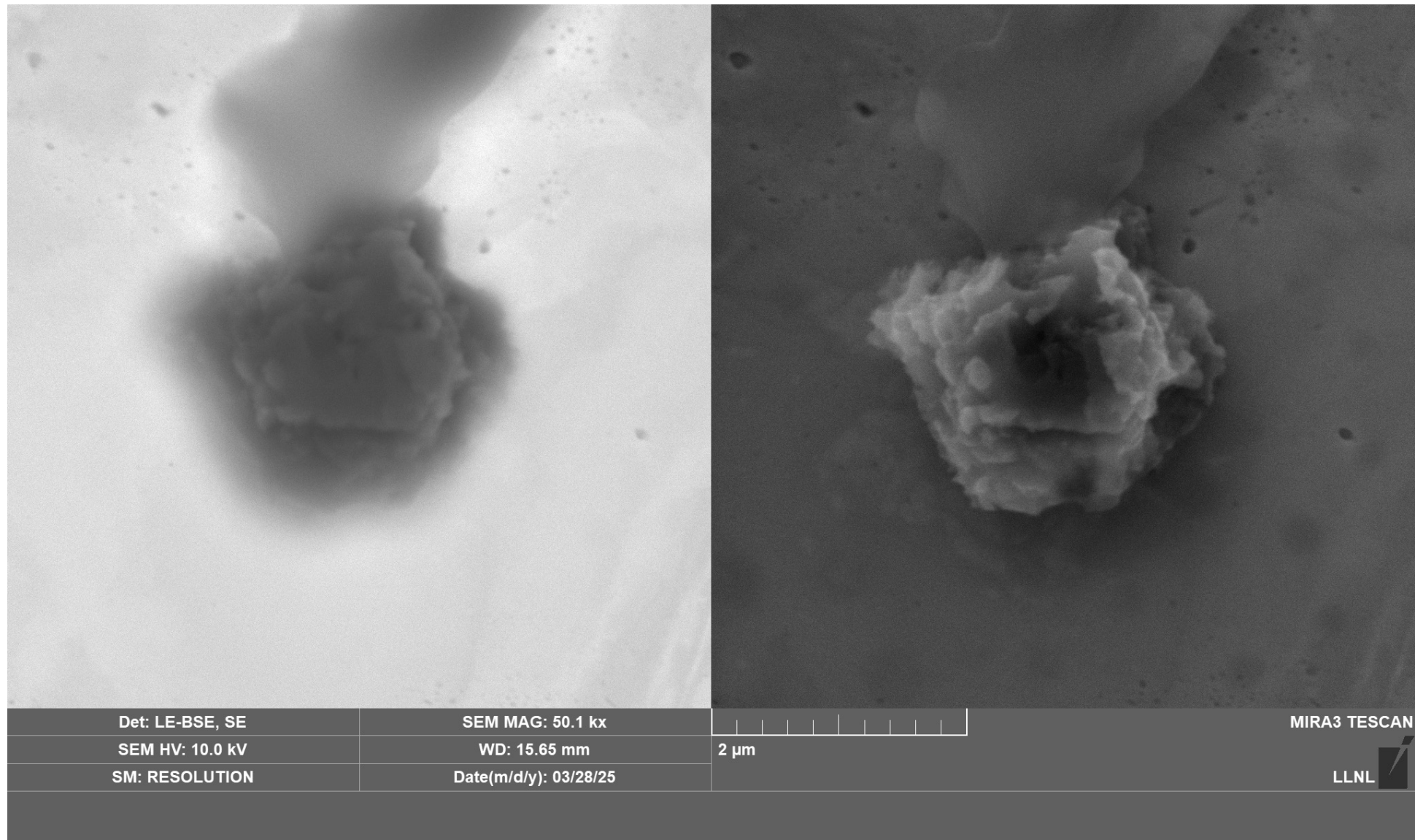

M

CO_ HE23_08

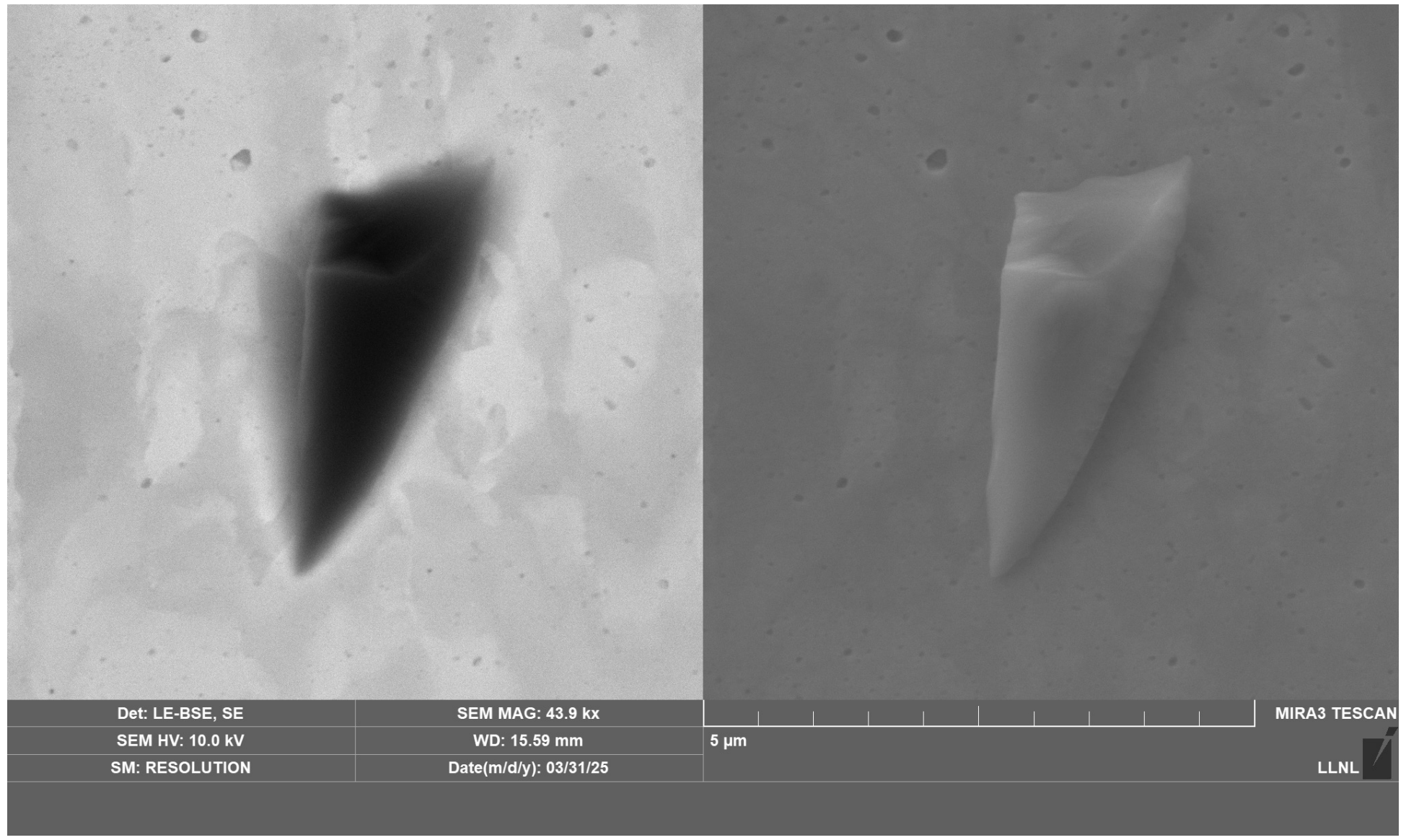

CO_ HE24_01

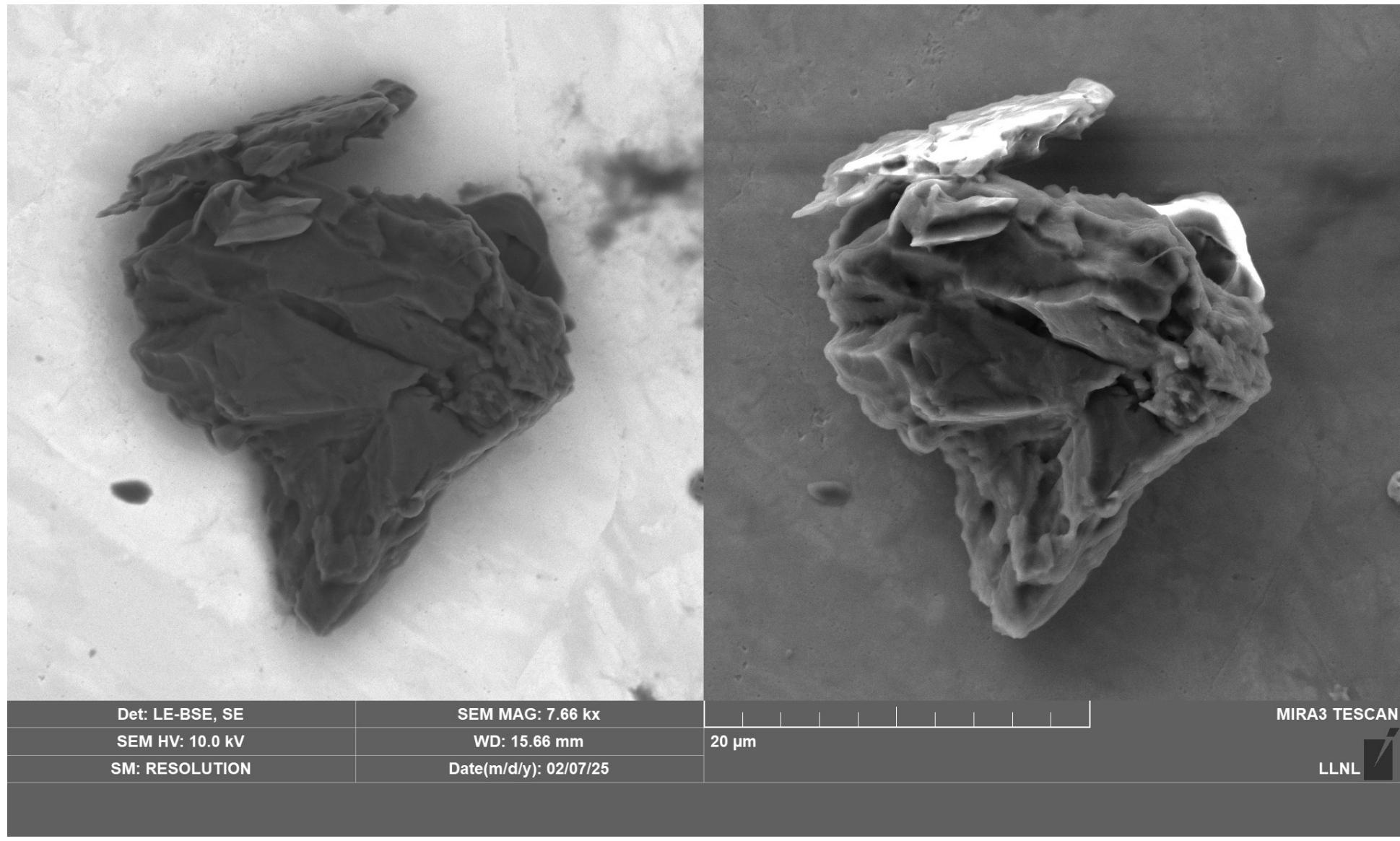

CO_ HE23_09

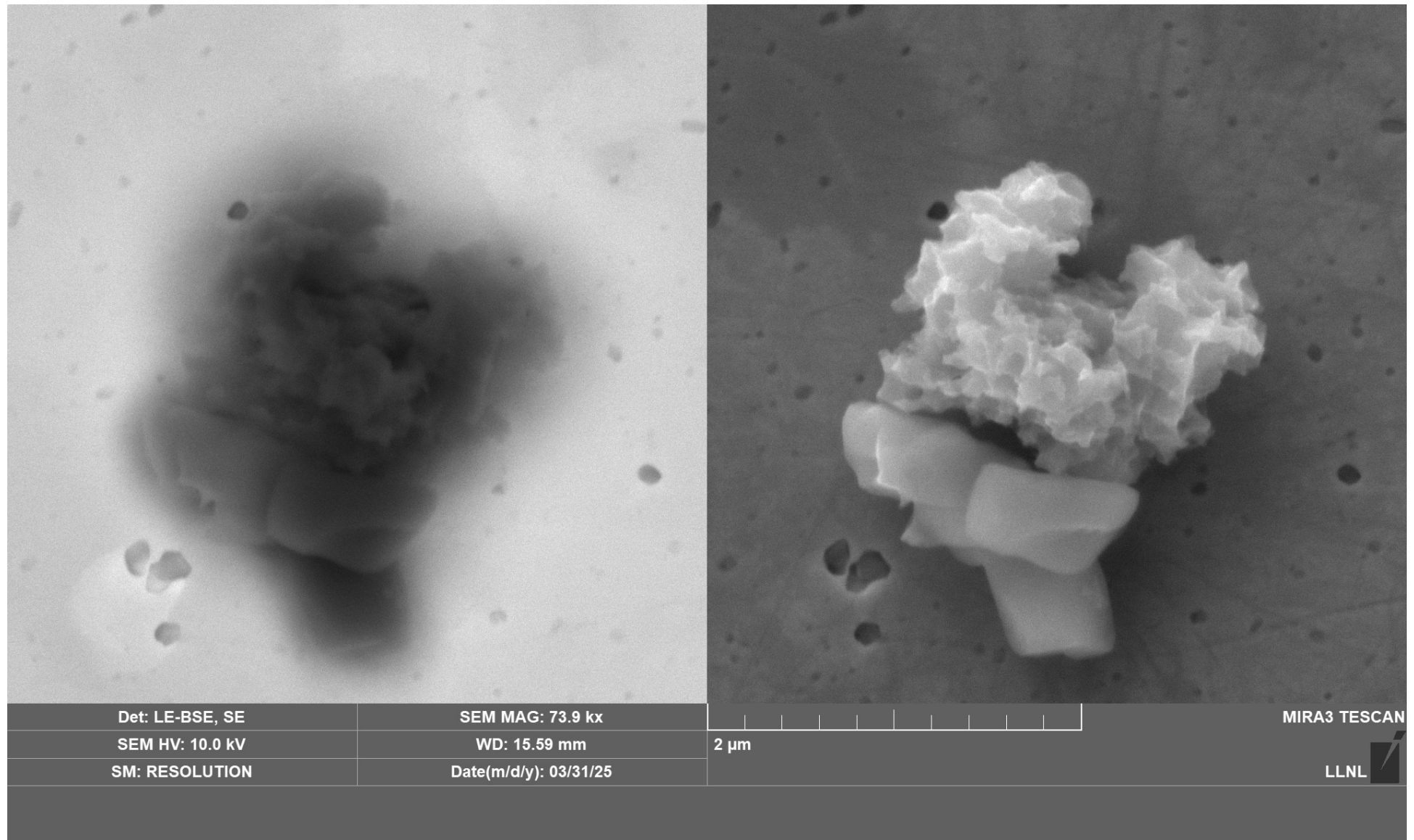

CO_ HE24_10

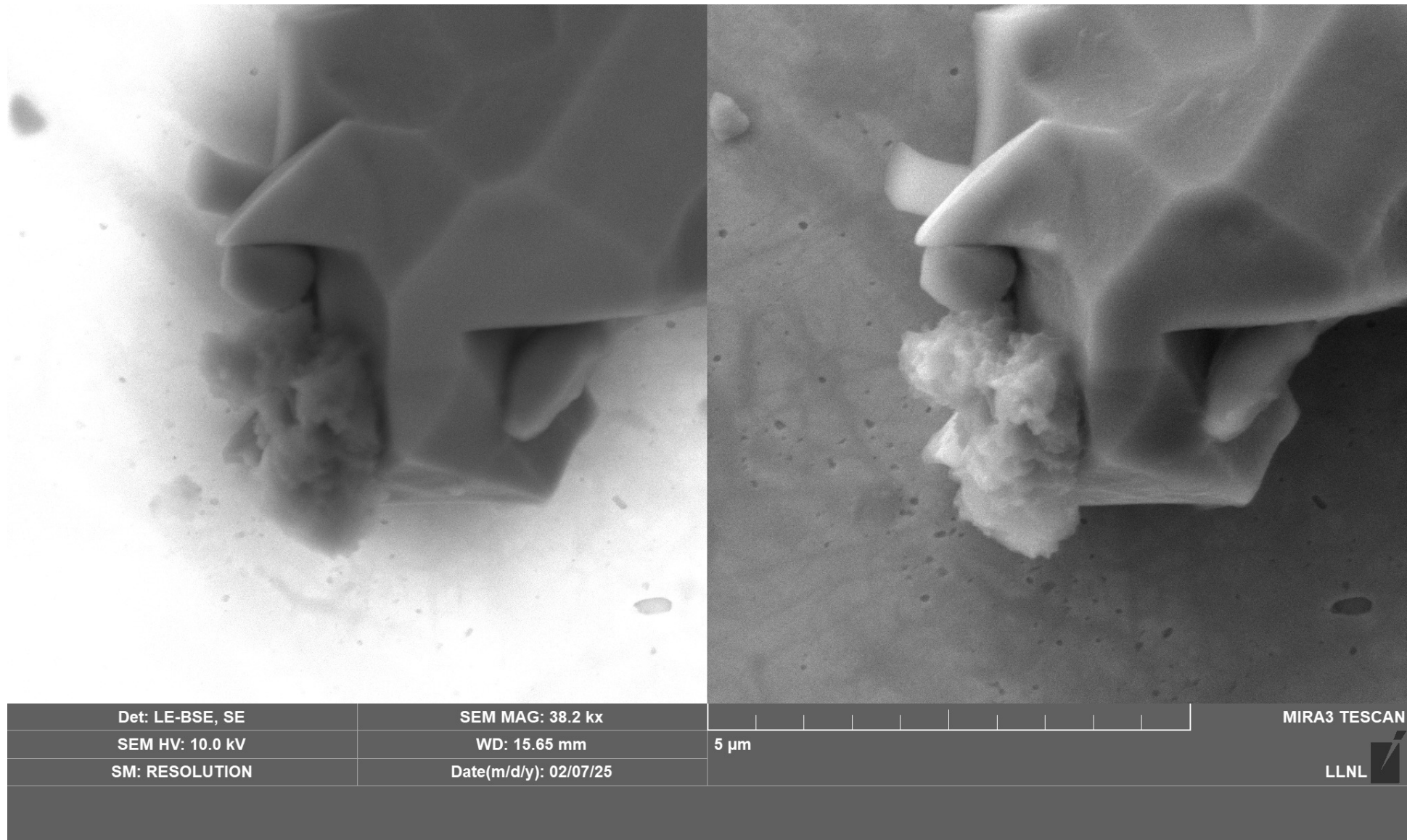

M

CO_ HE25_02

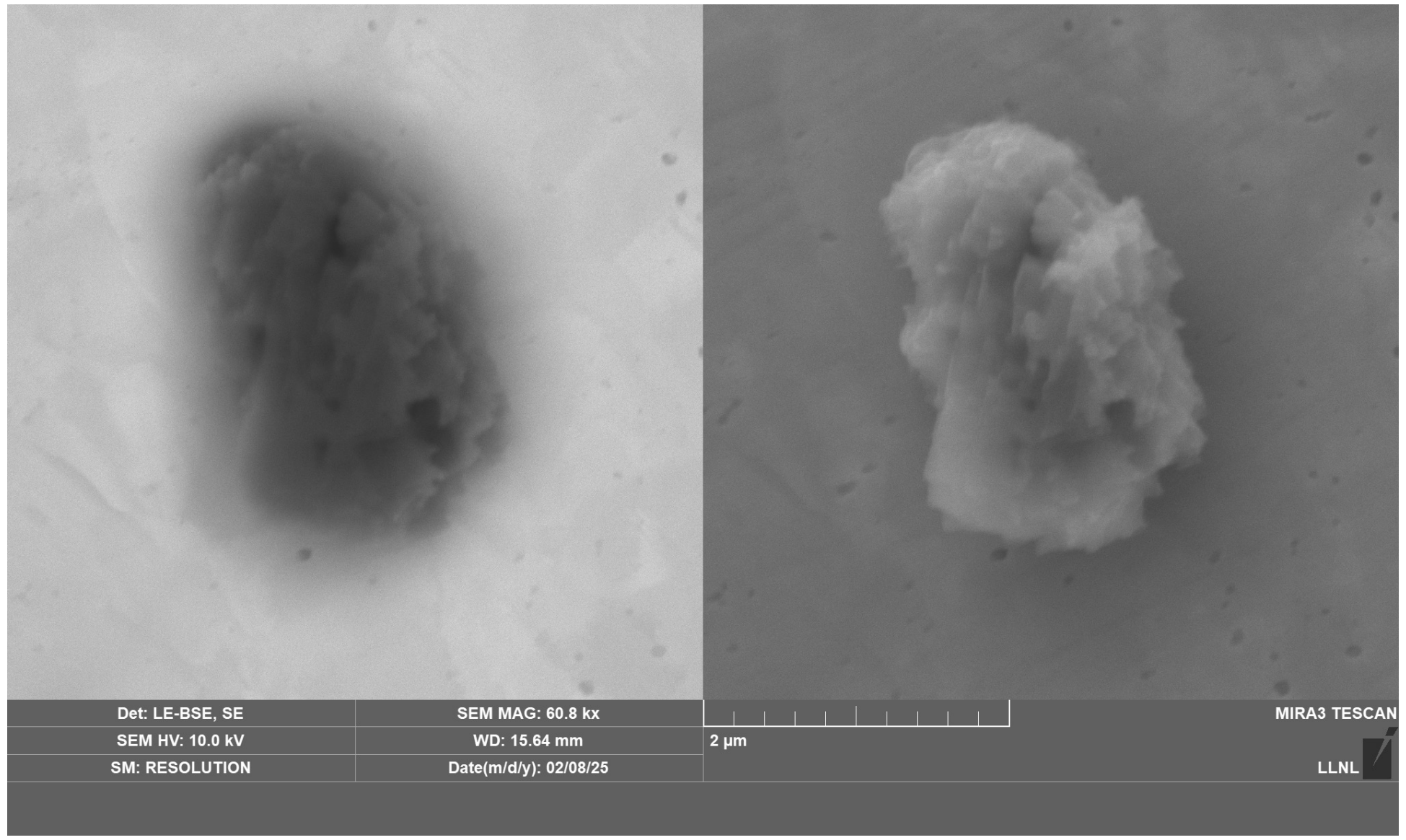

CO_ HE25_04

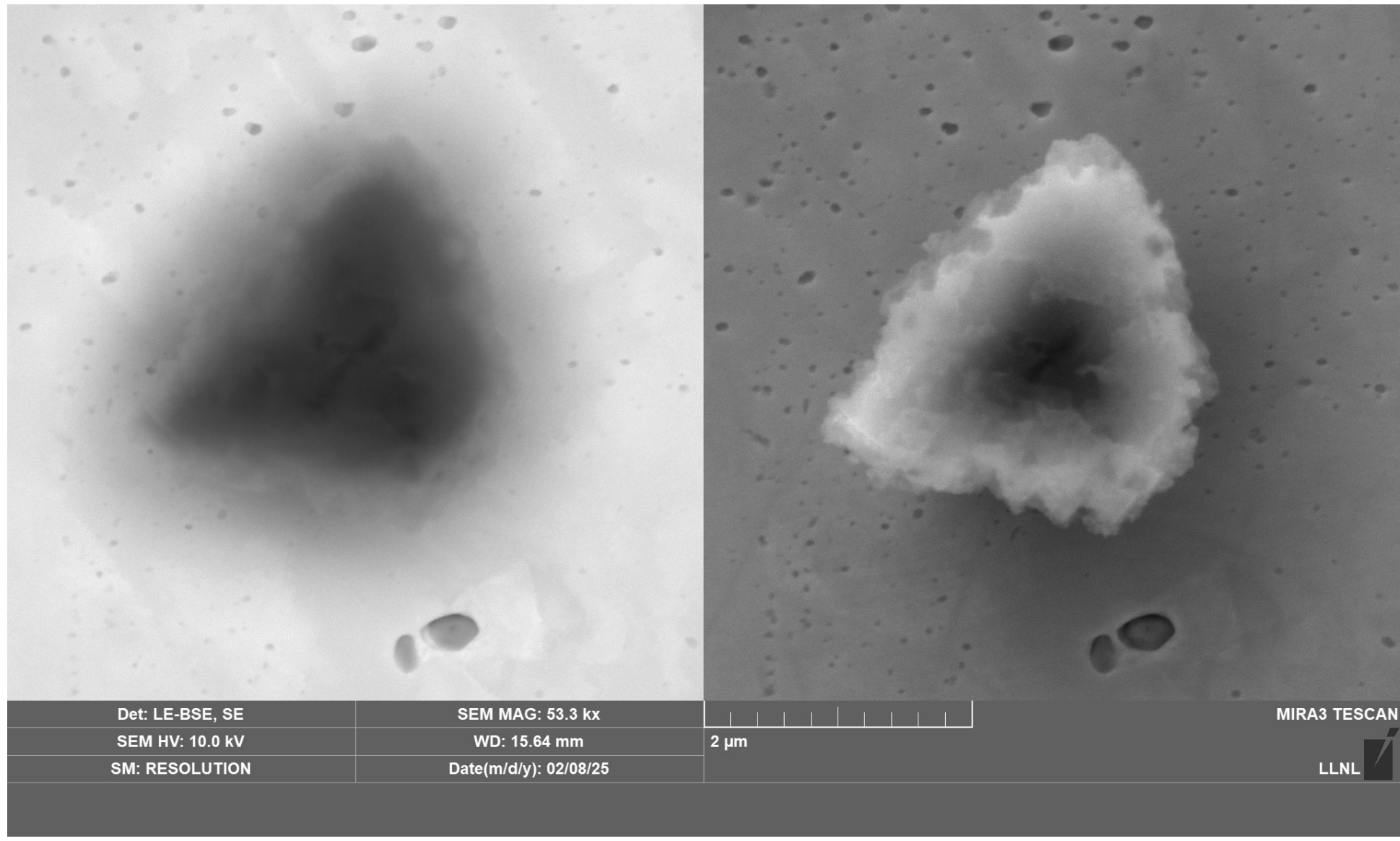

CO_ HE25_03

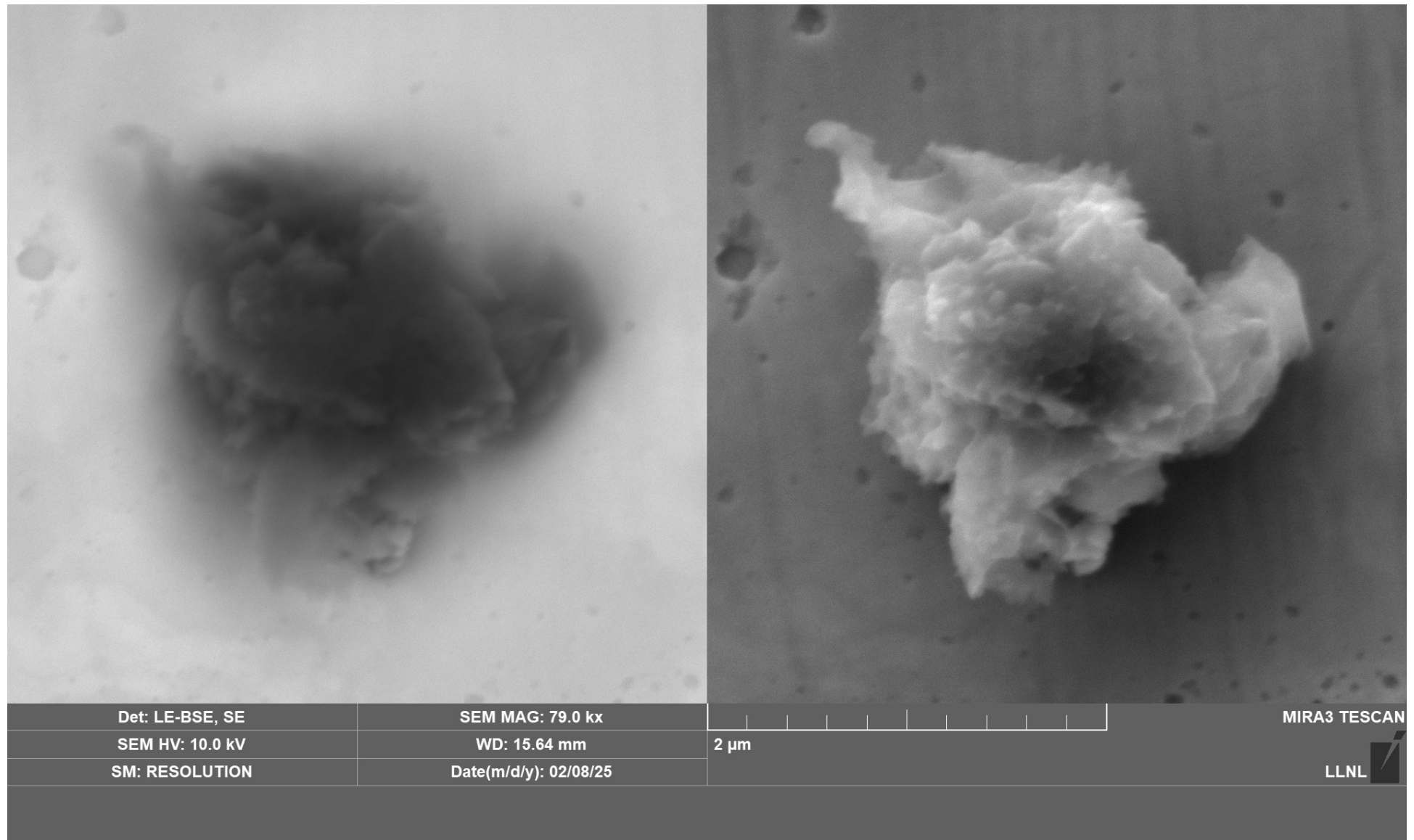

CO_ HE25_08

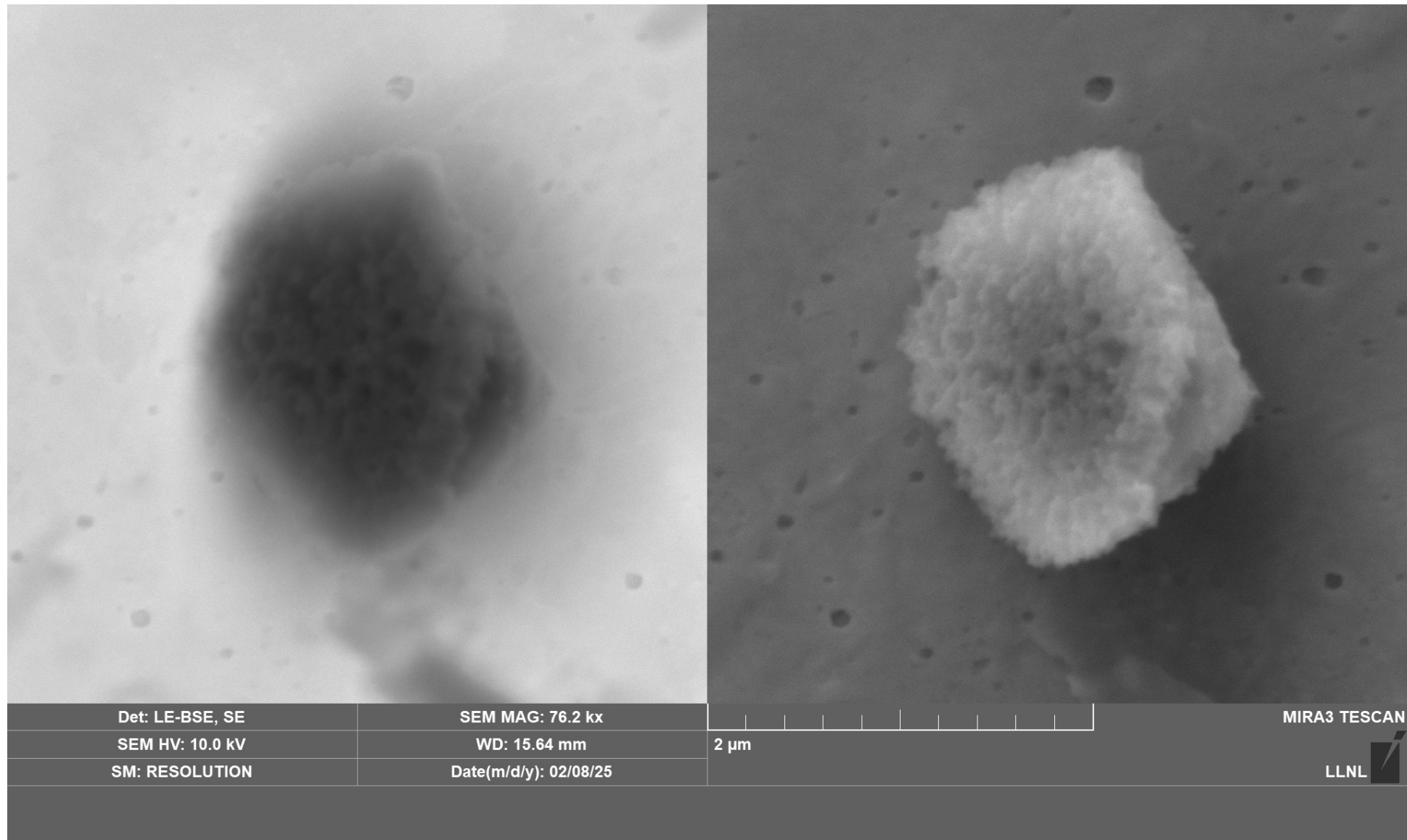

M

CO_ HE25_09

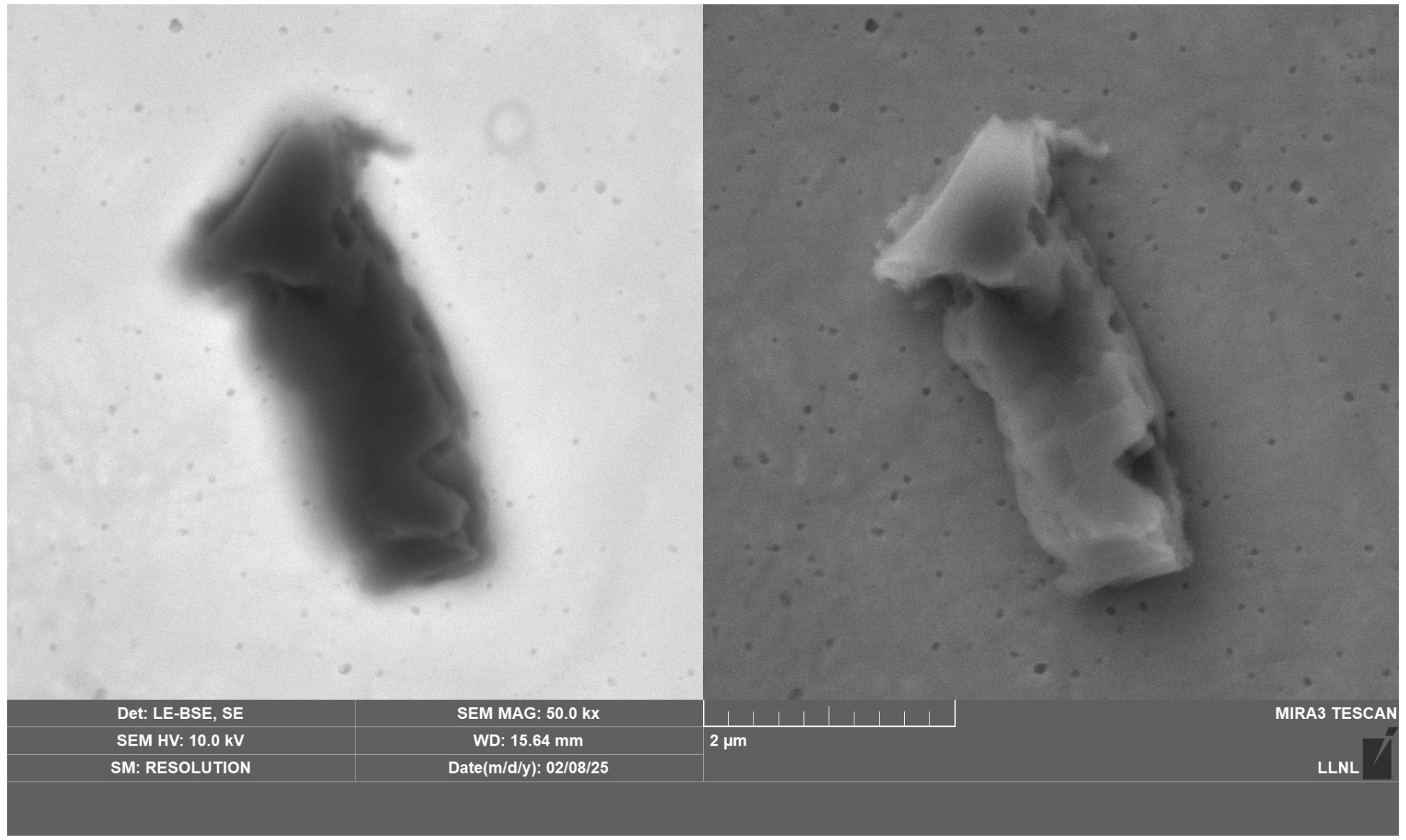

CO_ HE28_02

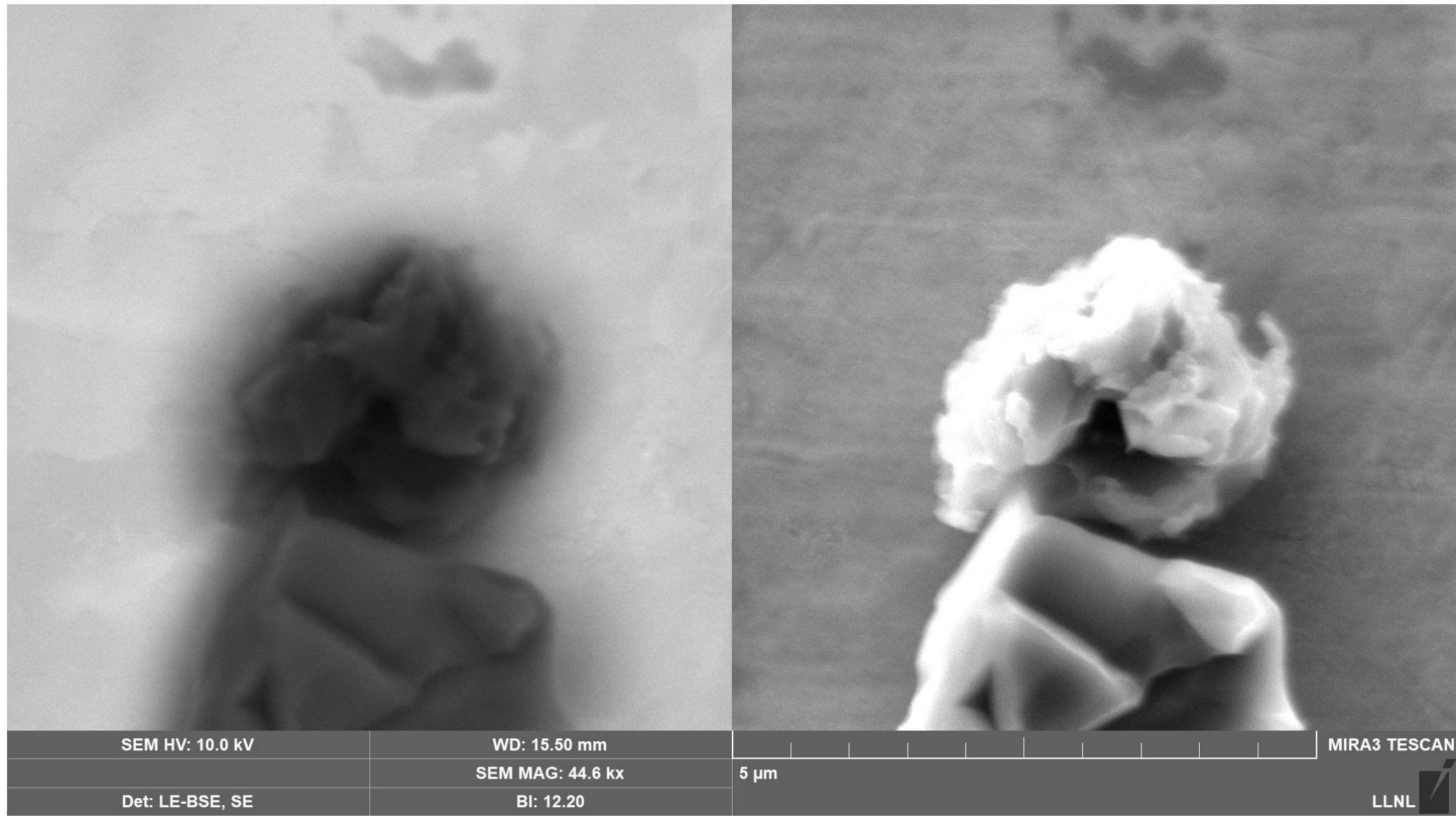

CO_ HE28_01

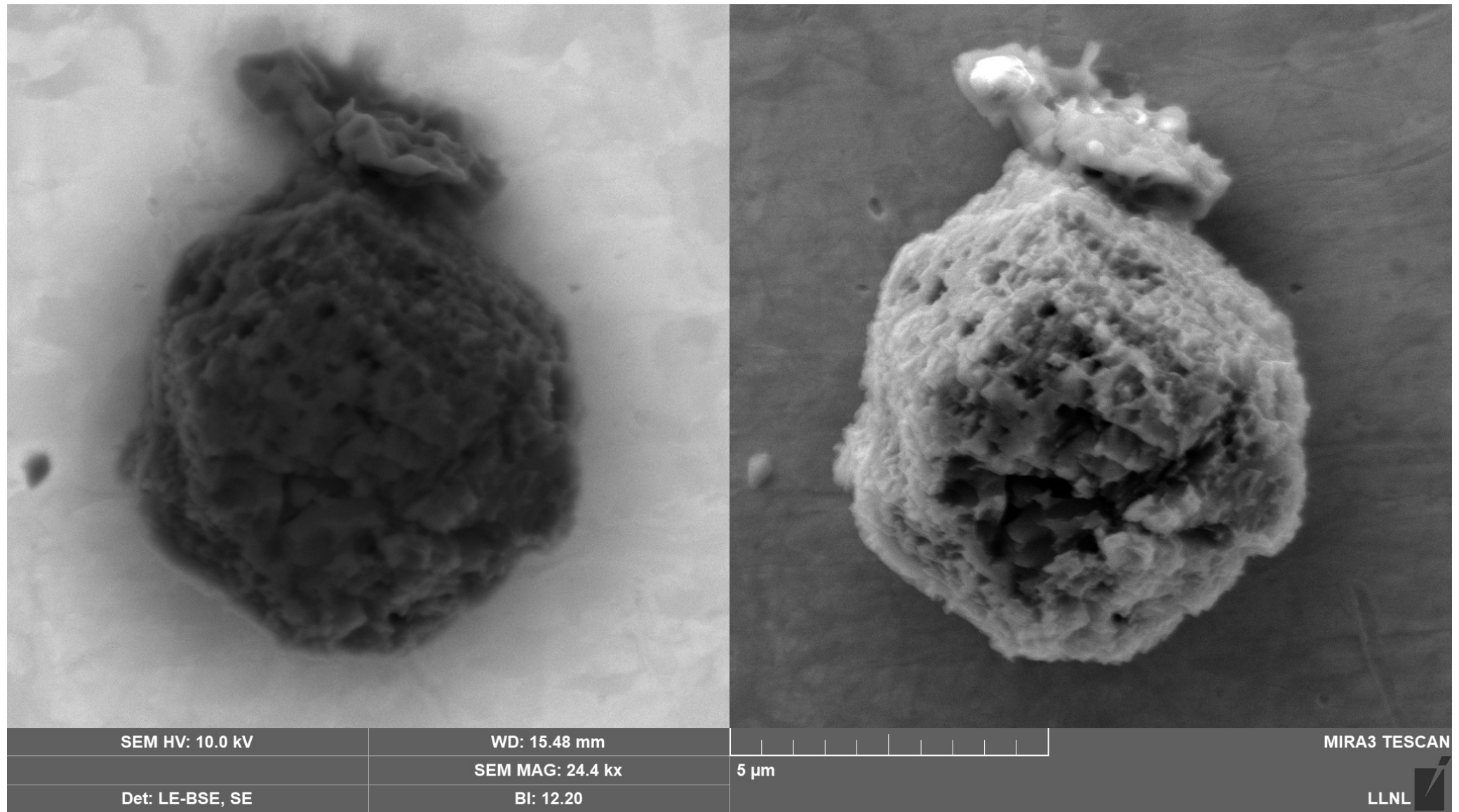

CO_ HE28_03

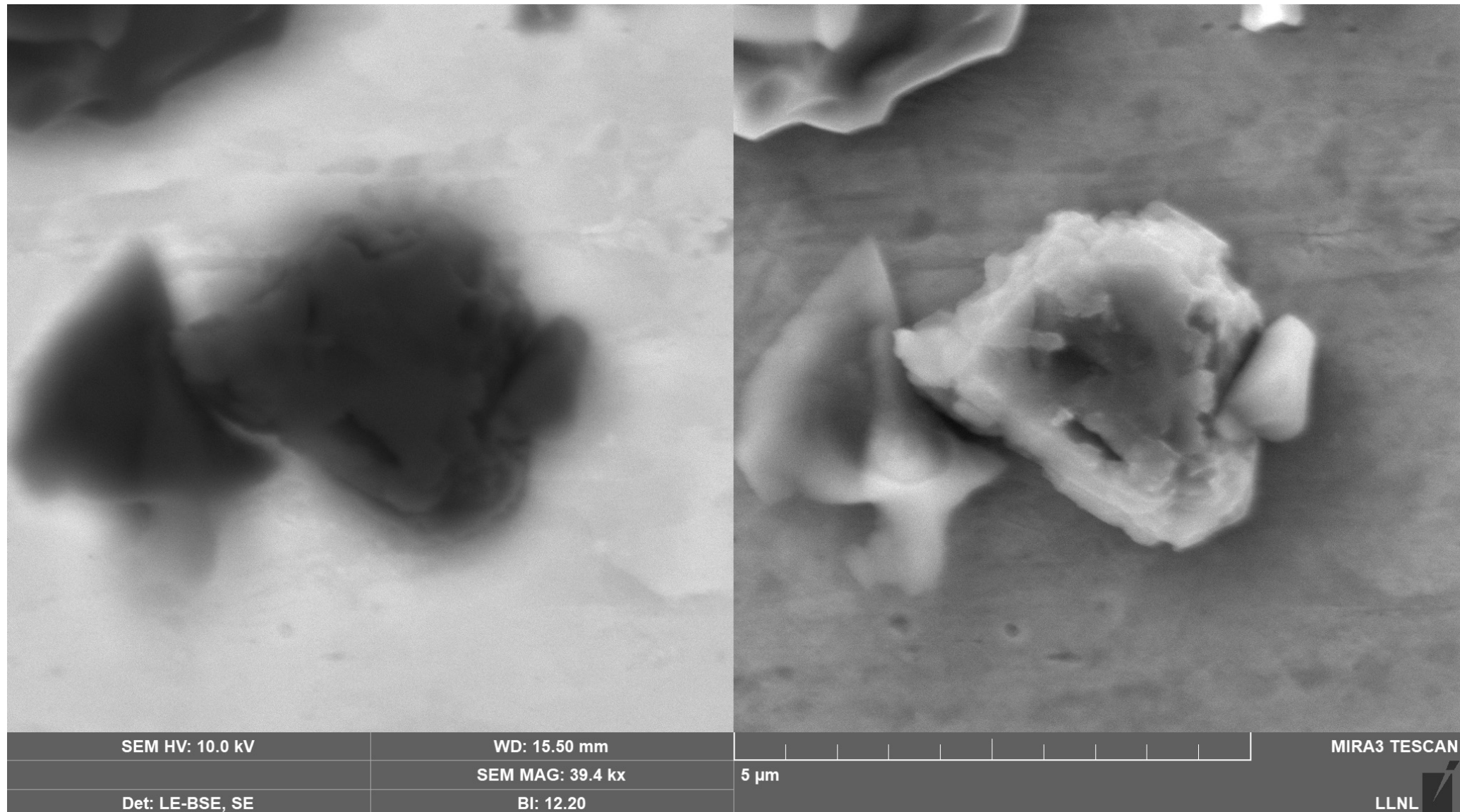

M

CO_ HE28_04

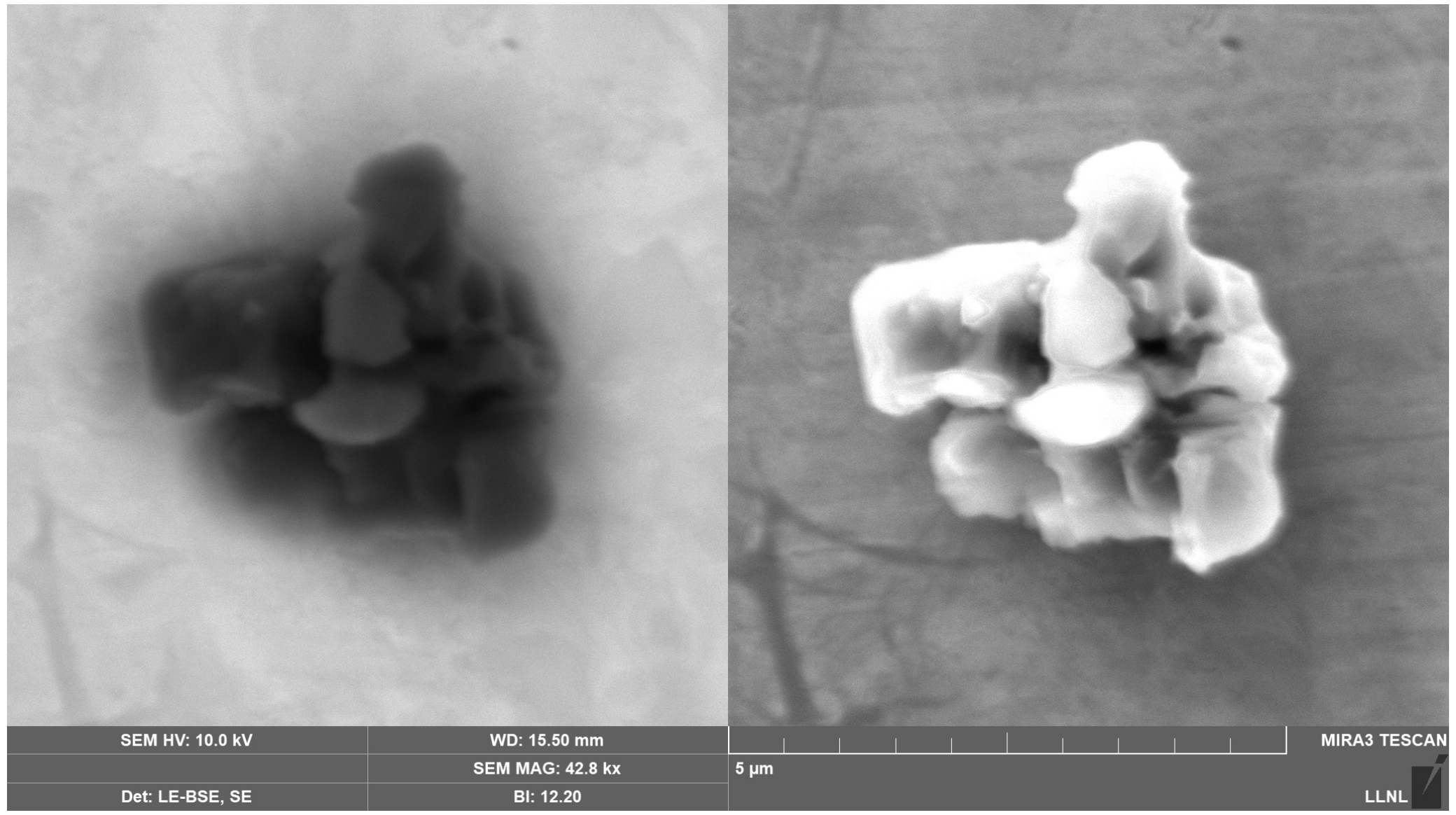

CO_ HE28_07

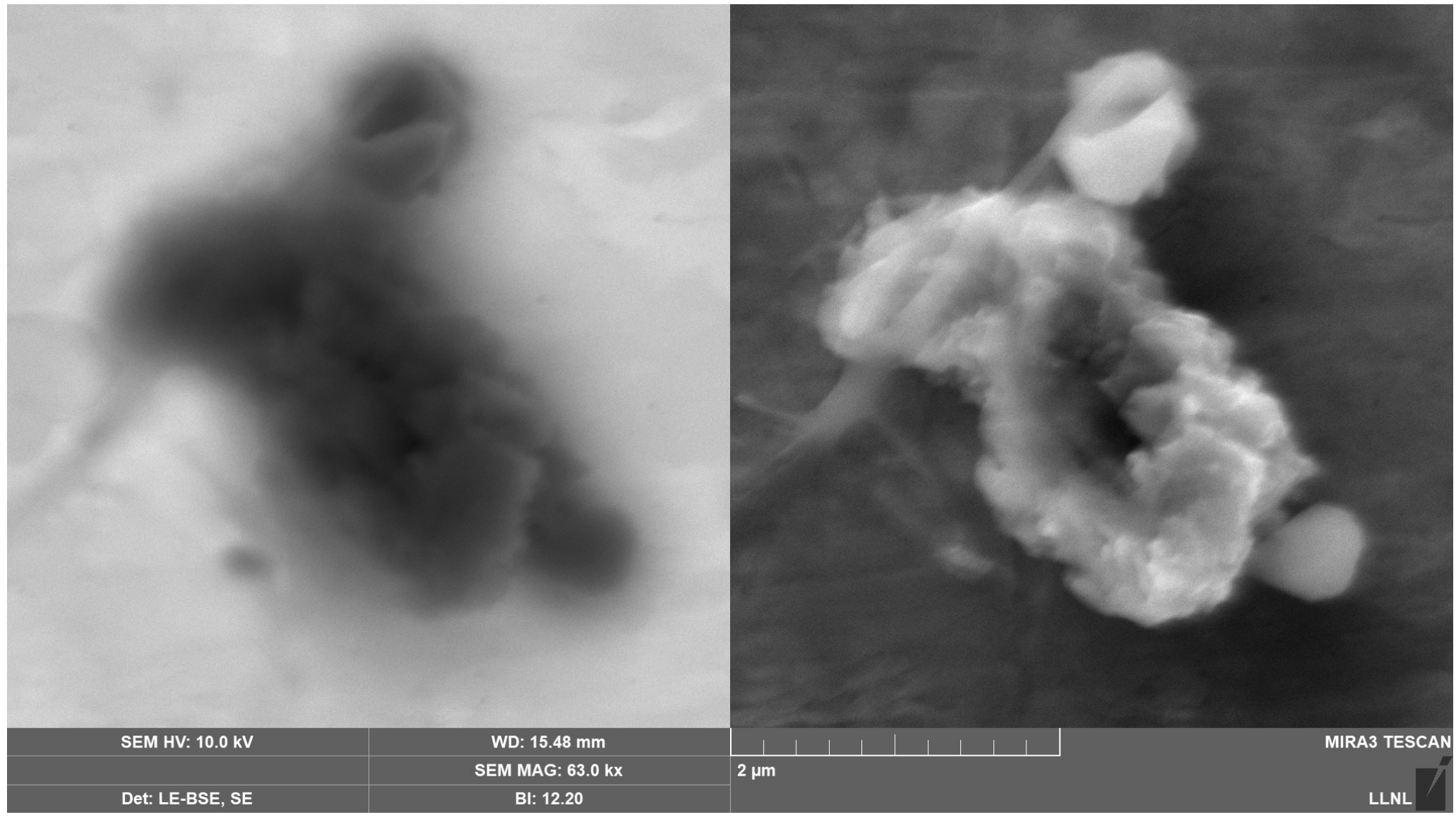

CO_ HE28_06

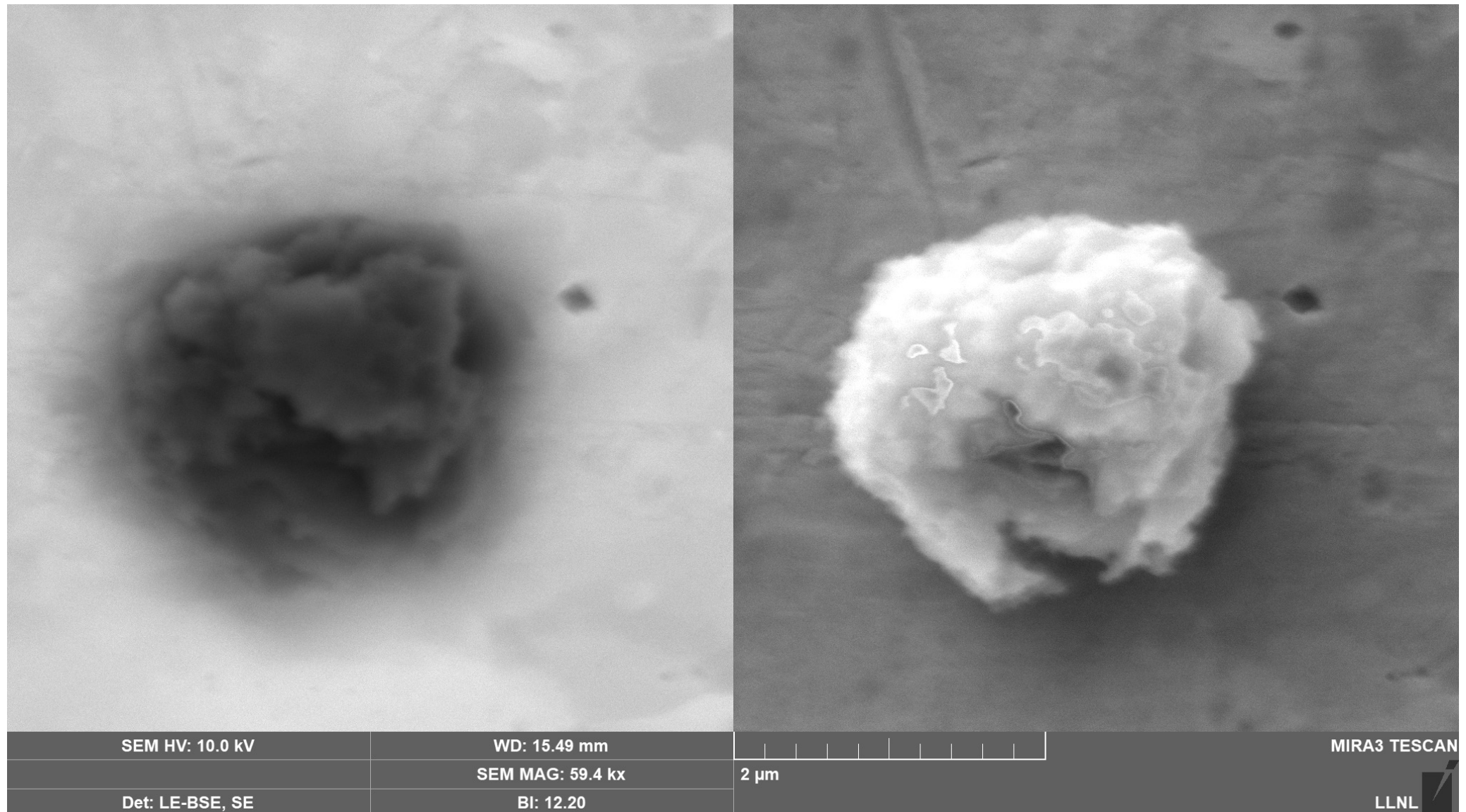

CO_ HE28_08

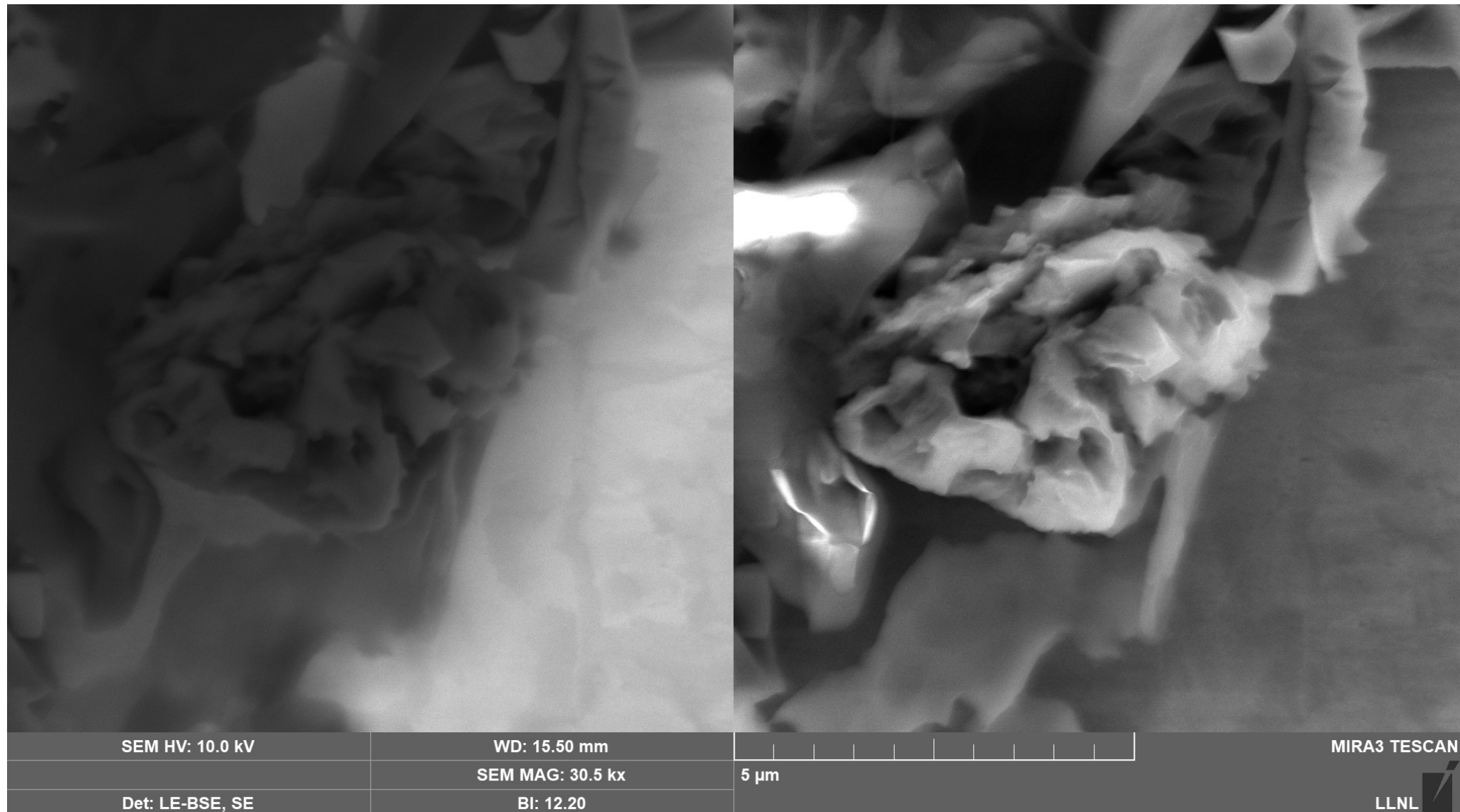

M

CO_ HE29_01

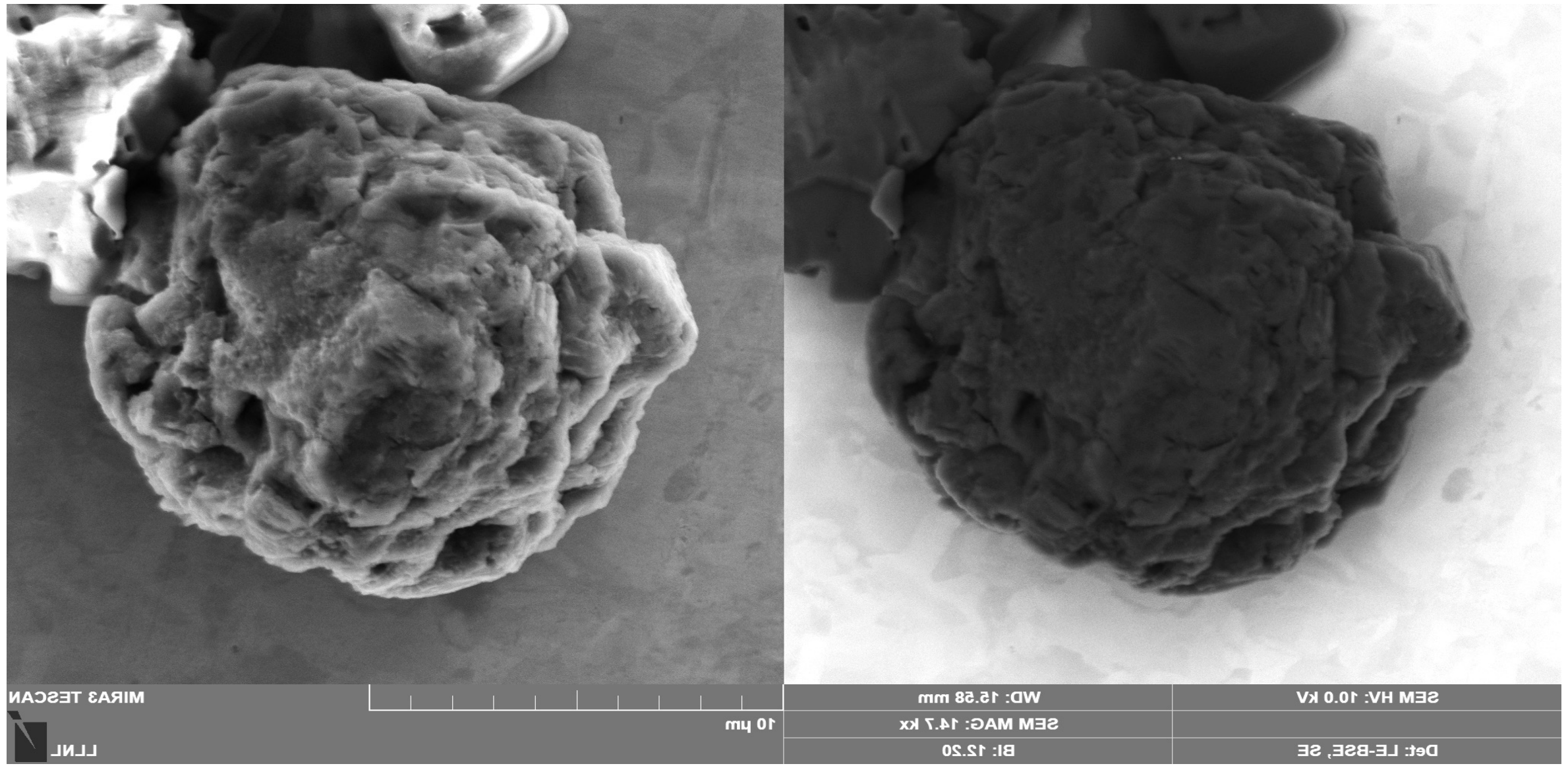

CO_ HE29_03

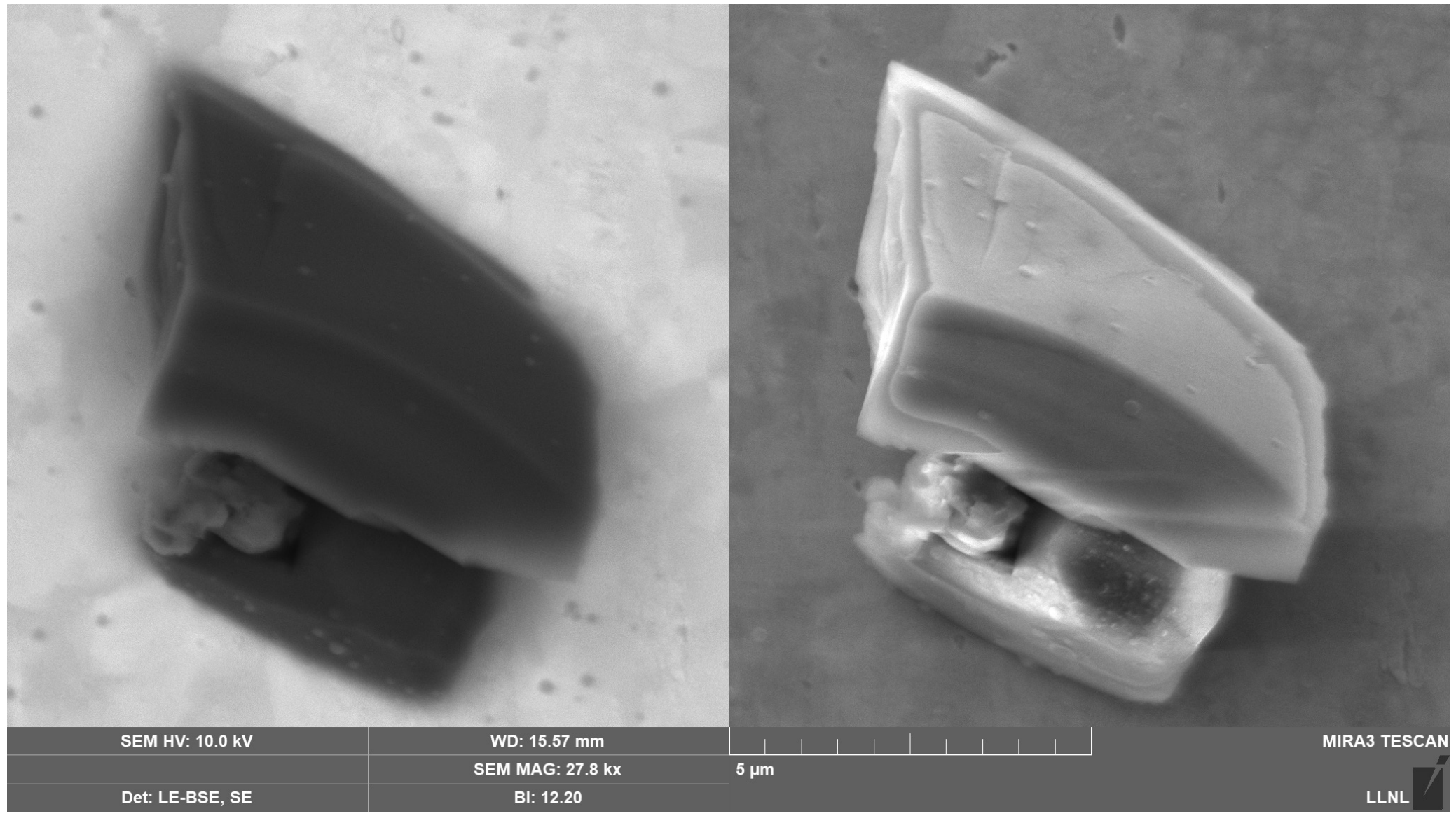

CO_ HE29_02

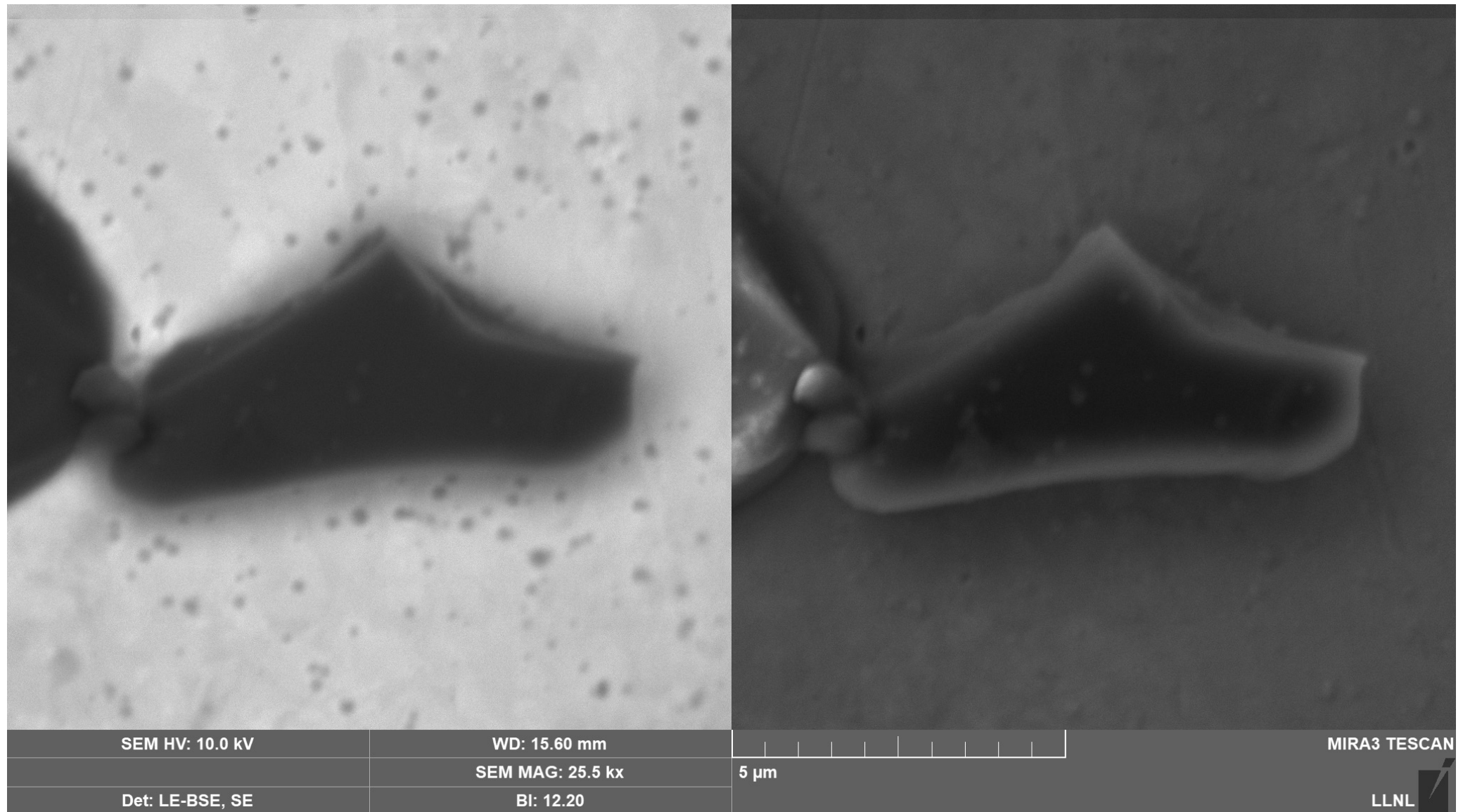

CO_ HE29_06

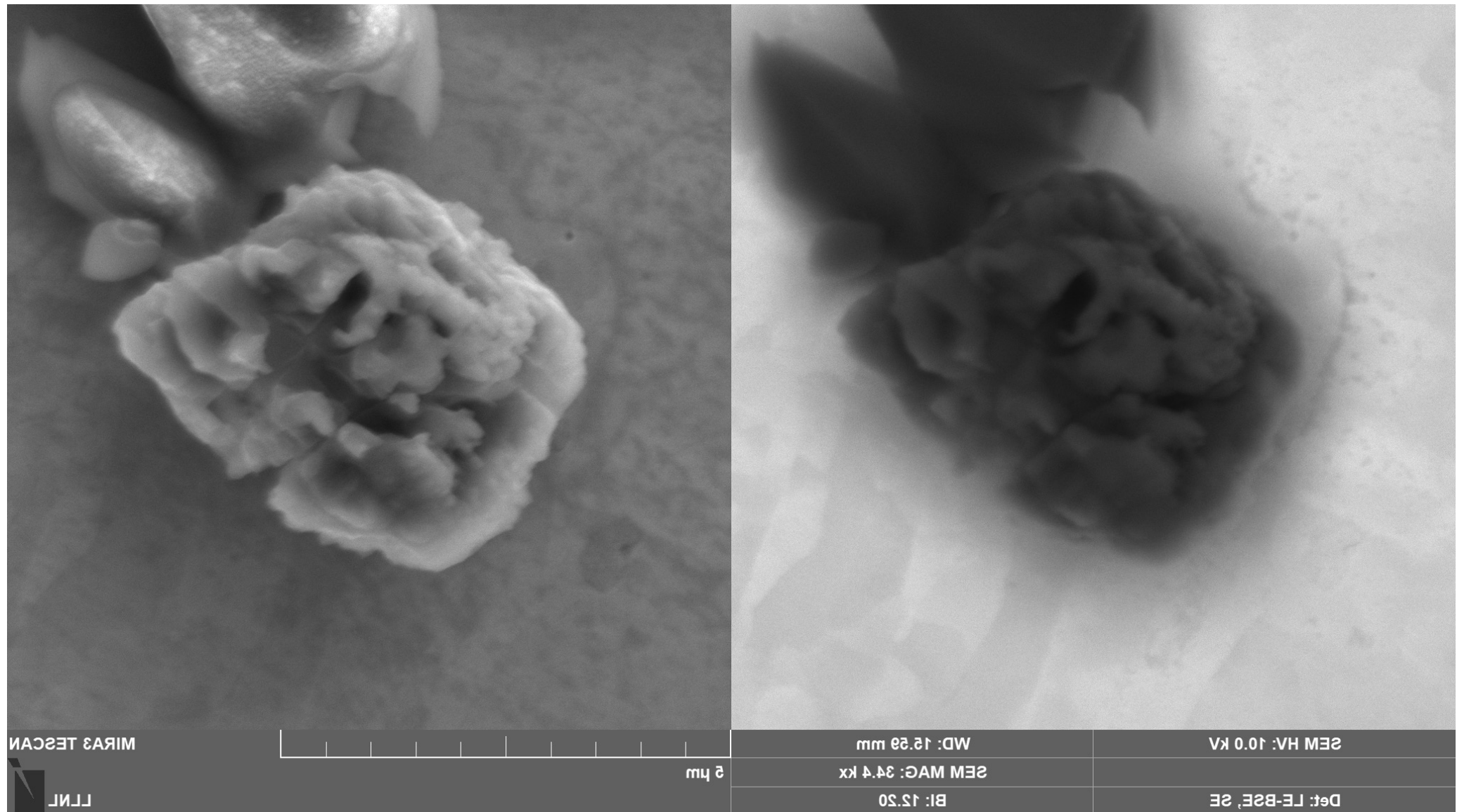

M

CO_ HE29_07

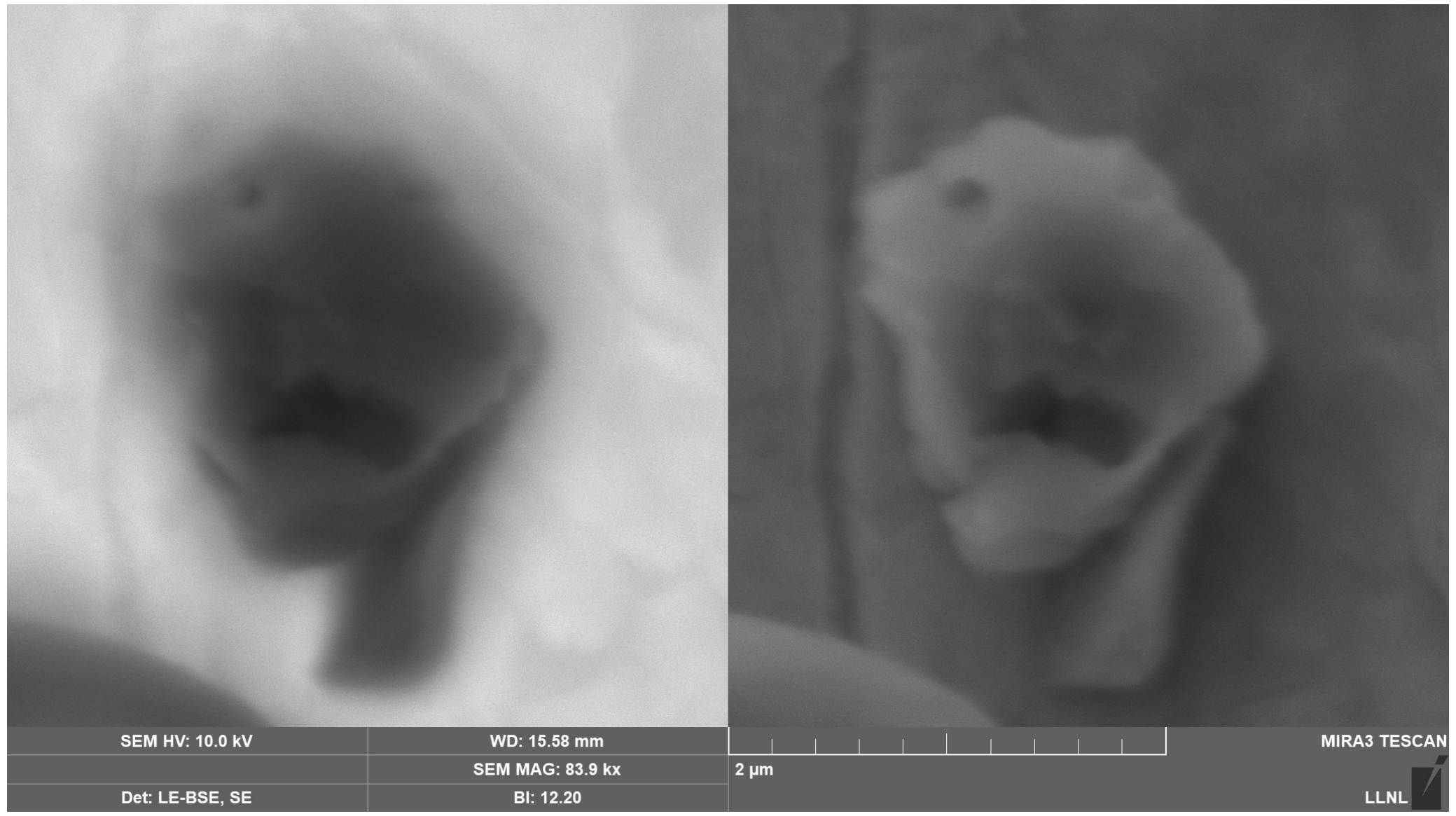

CO_ HE29_08

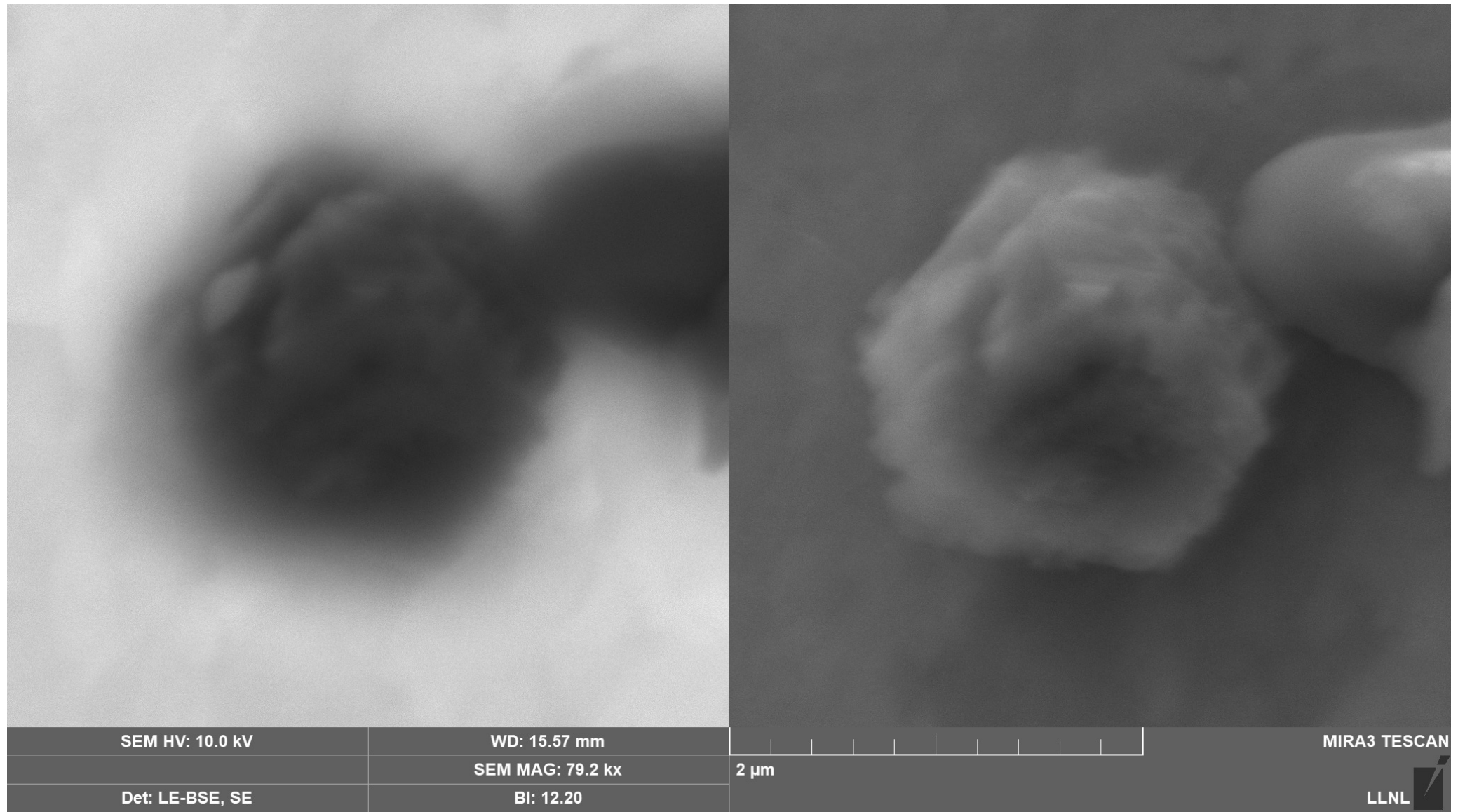

X CO_ HE23_01

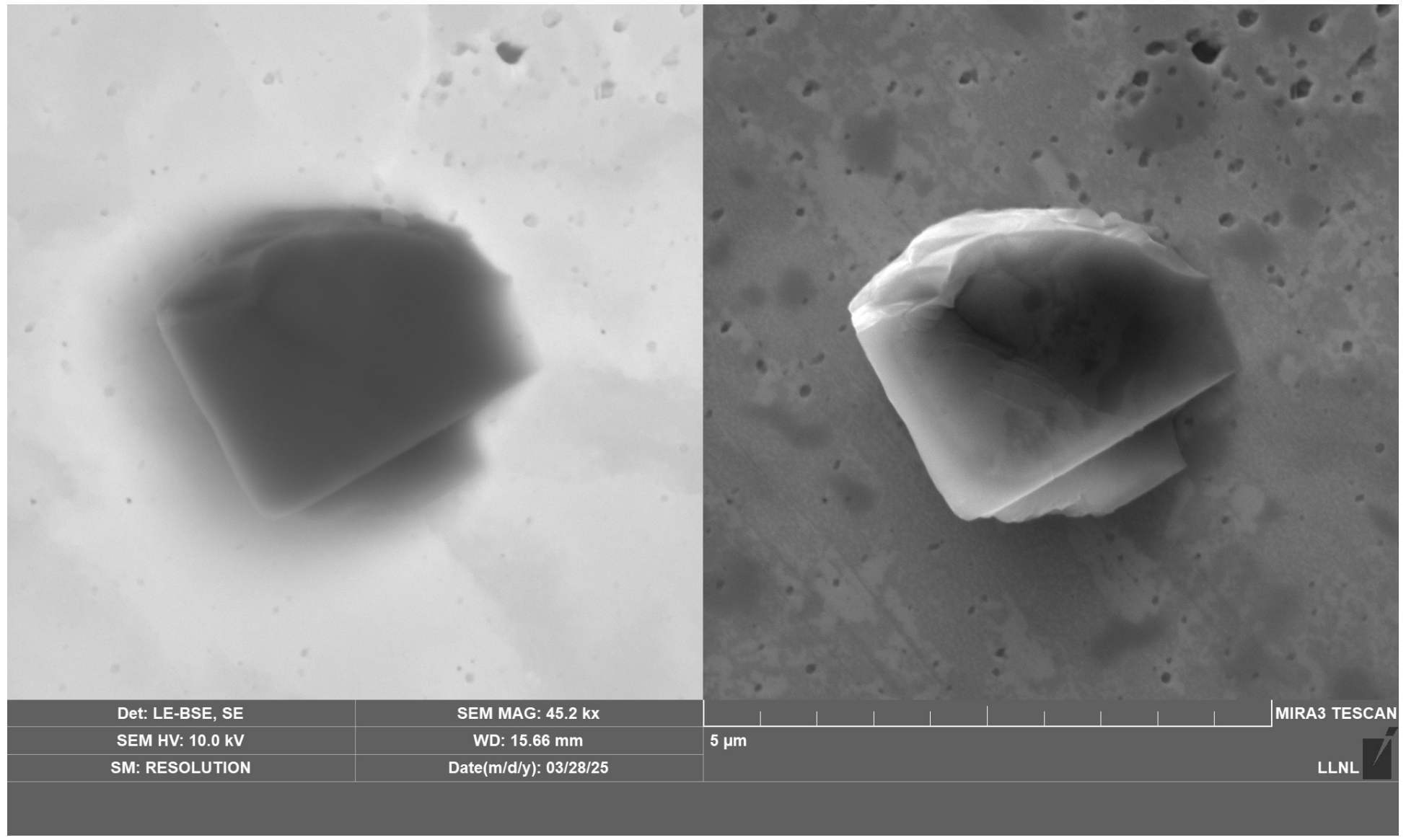

Y CO_ HE25_07

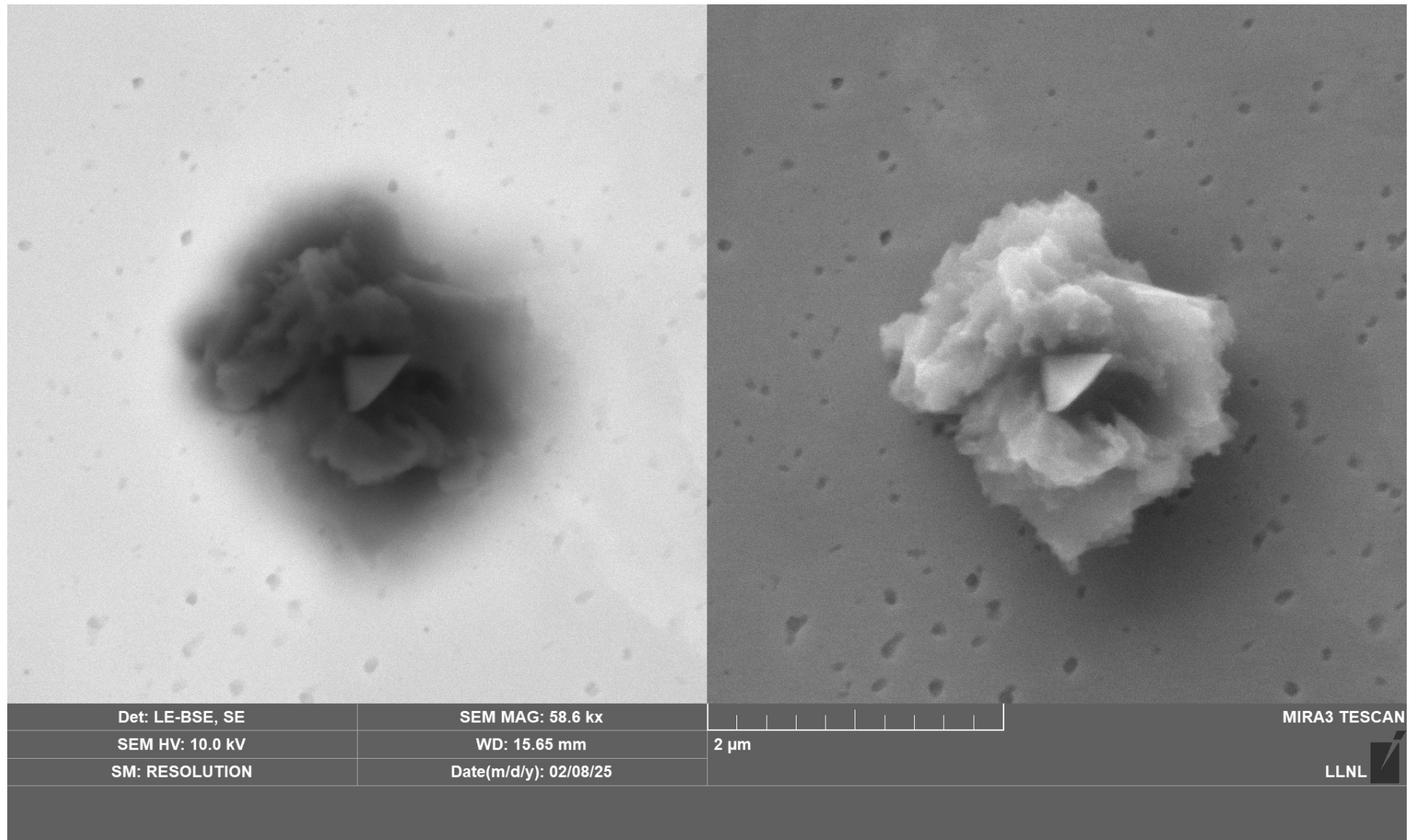

terrestrial

CO_GA07_32

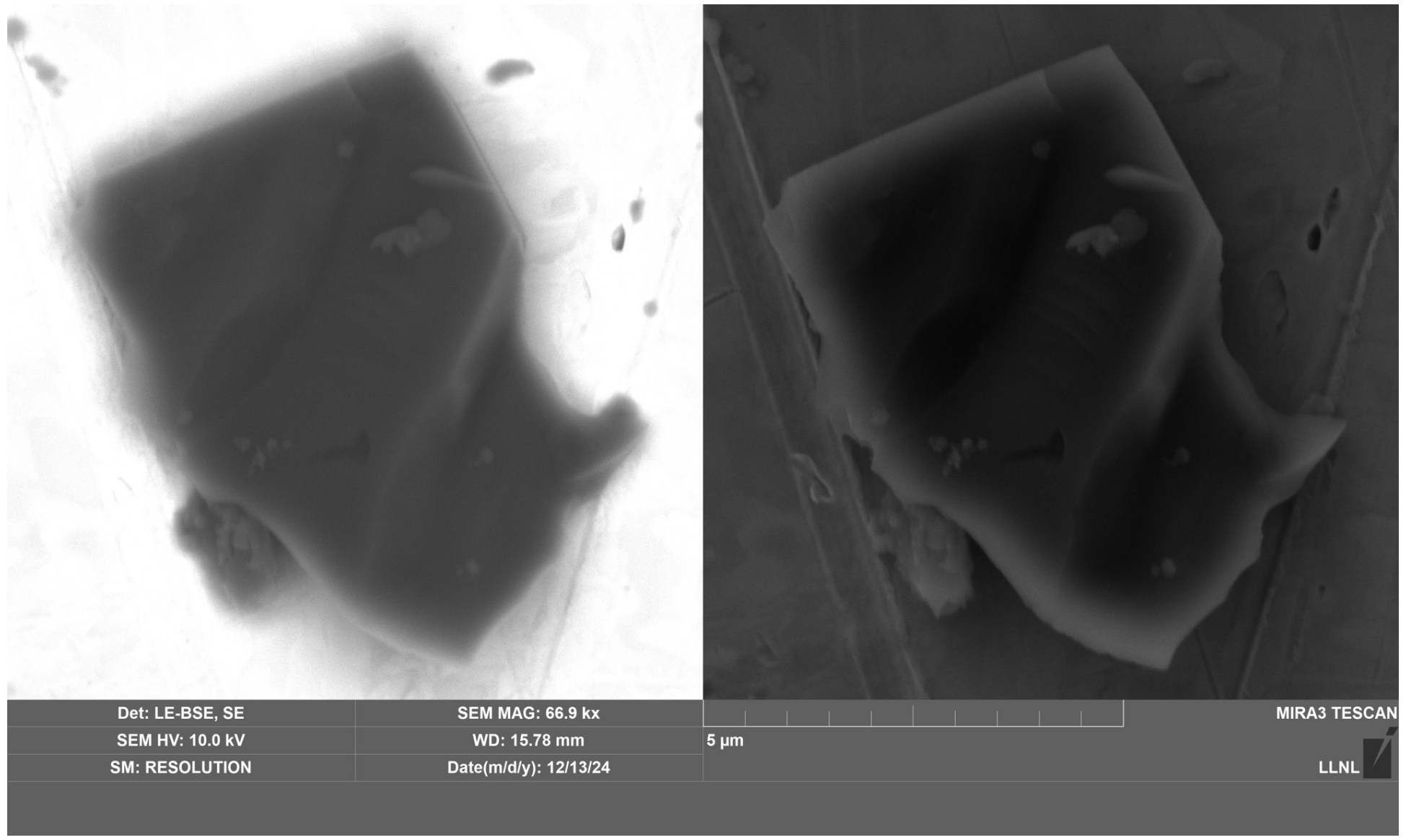

CO_ HE14_01

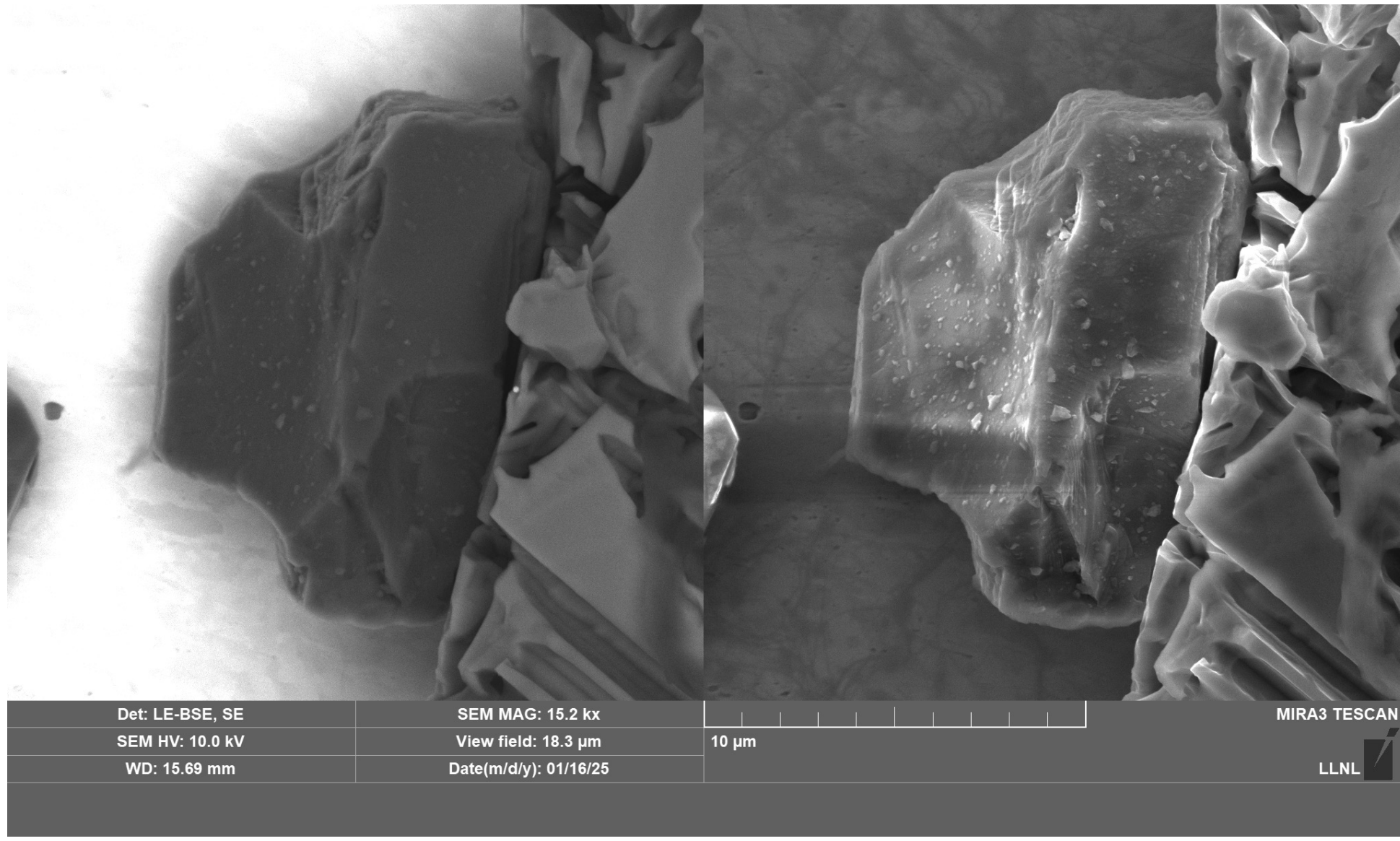

CO_GA08_01

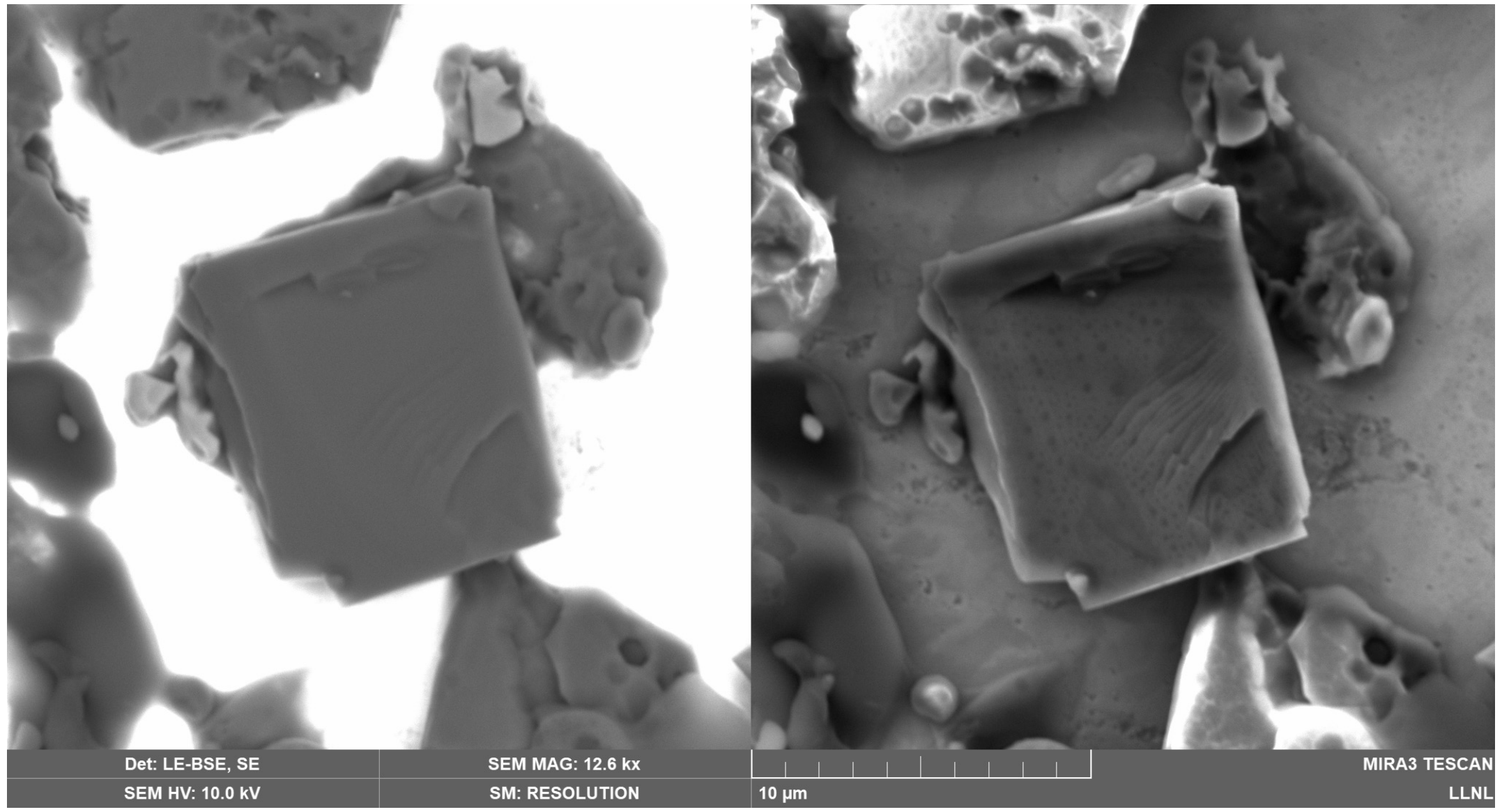

CO_ HE14_02

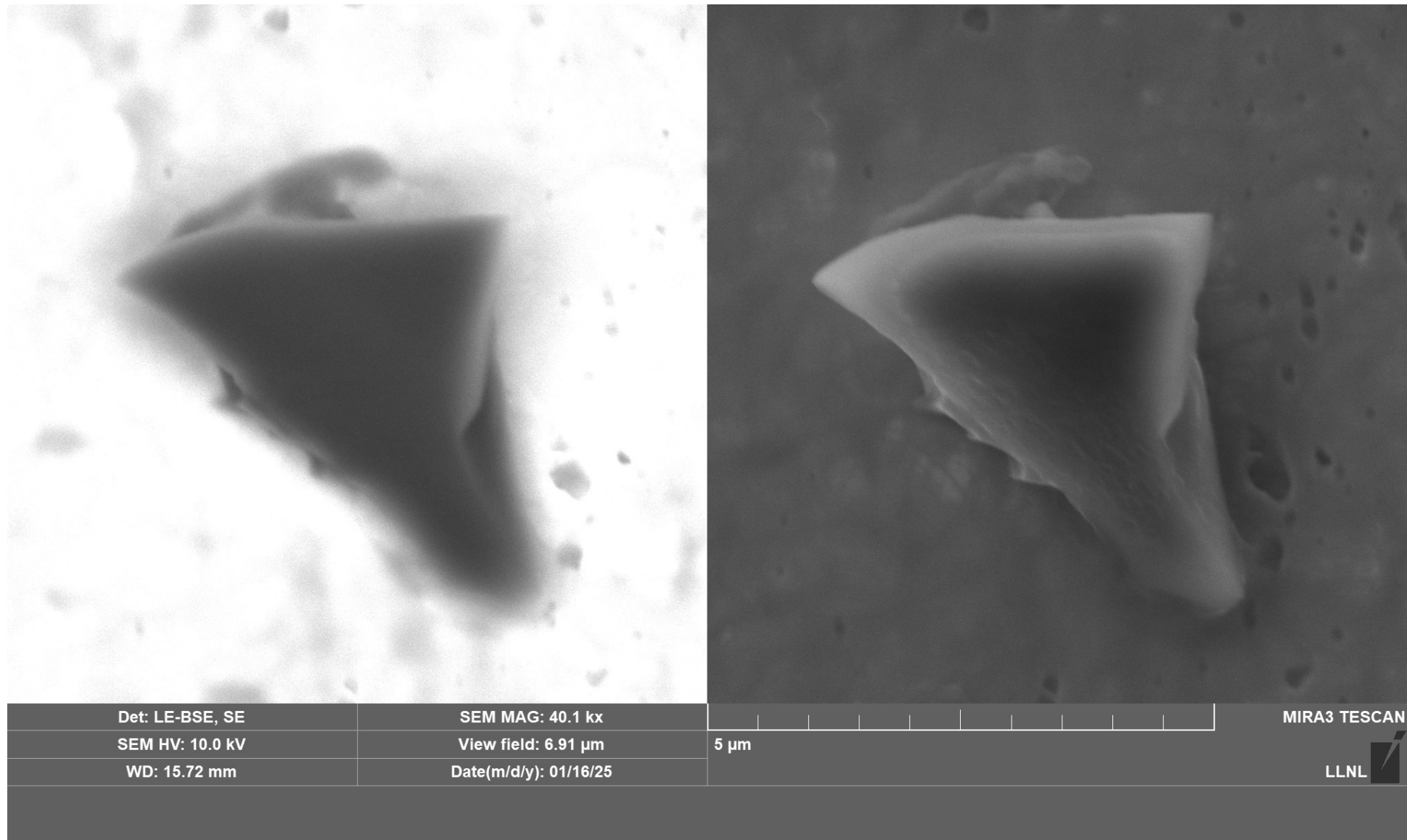

terrestrial

CO_ HE14_03

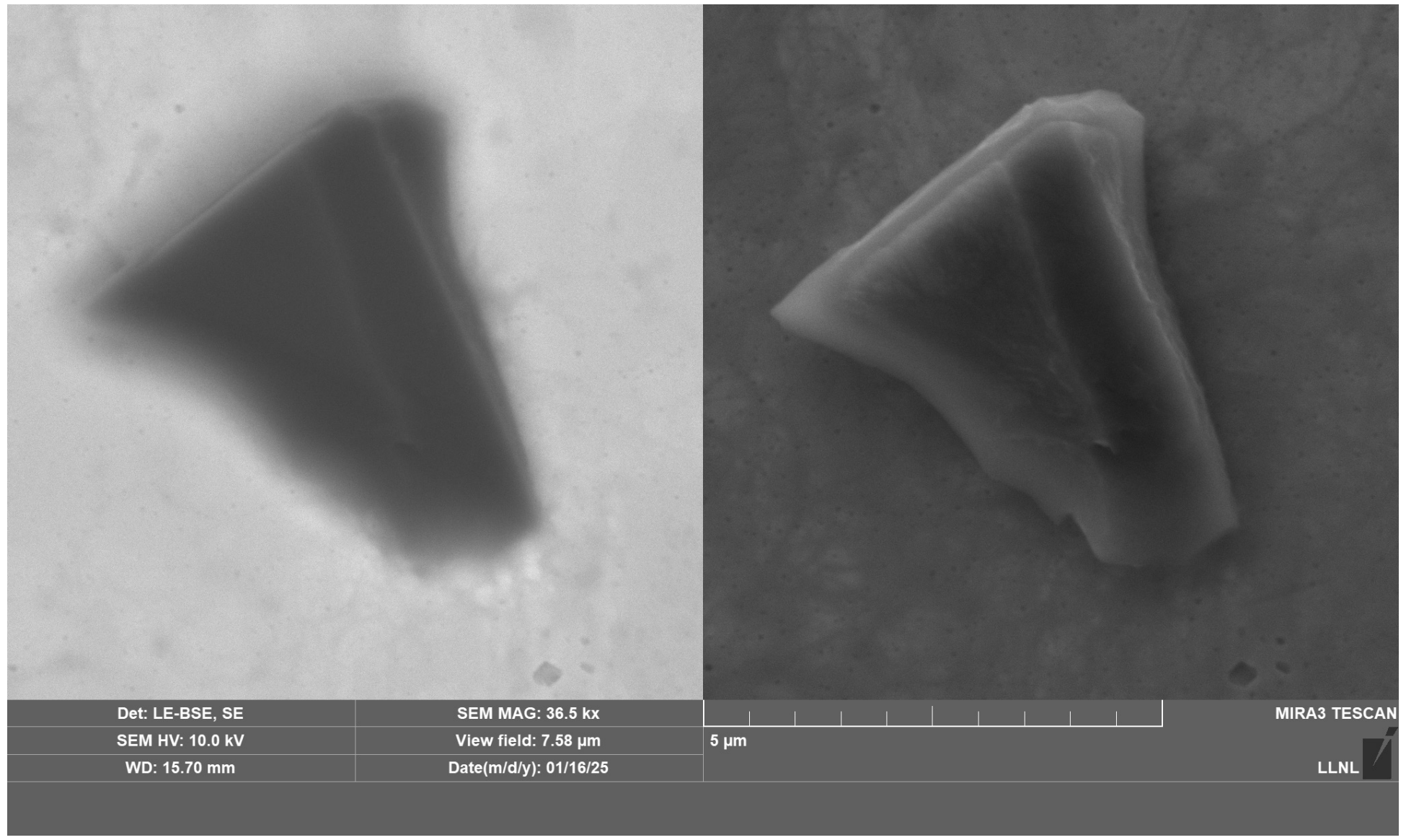

CO_ HE19_01

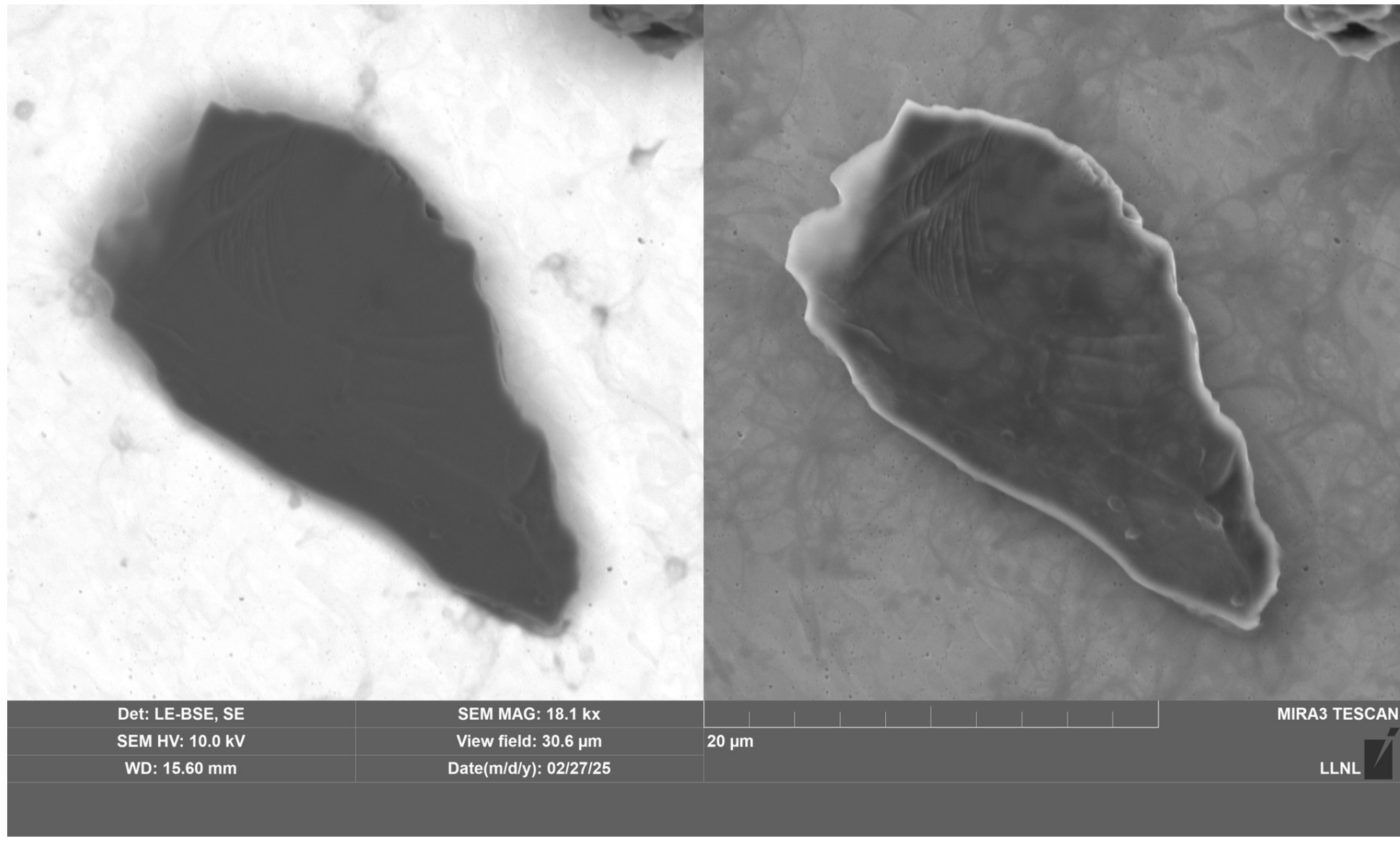

CO_ HE18_01

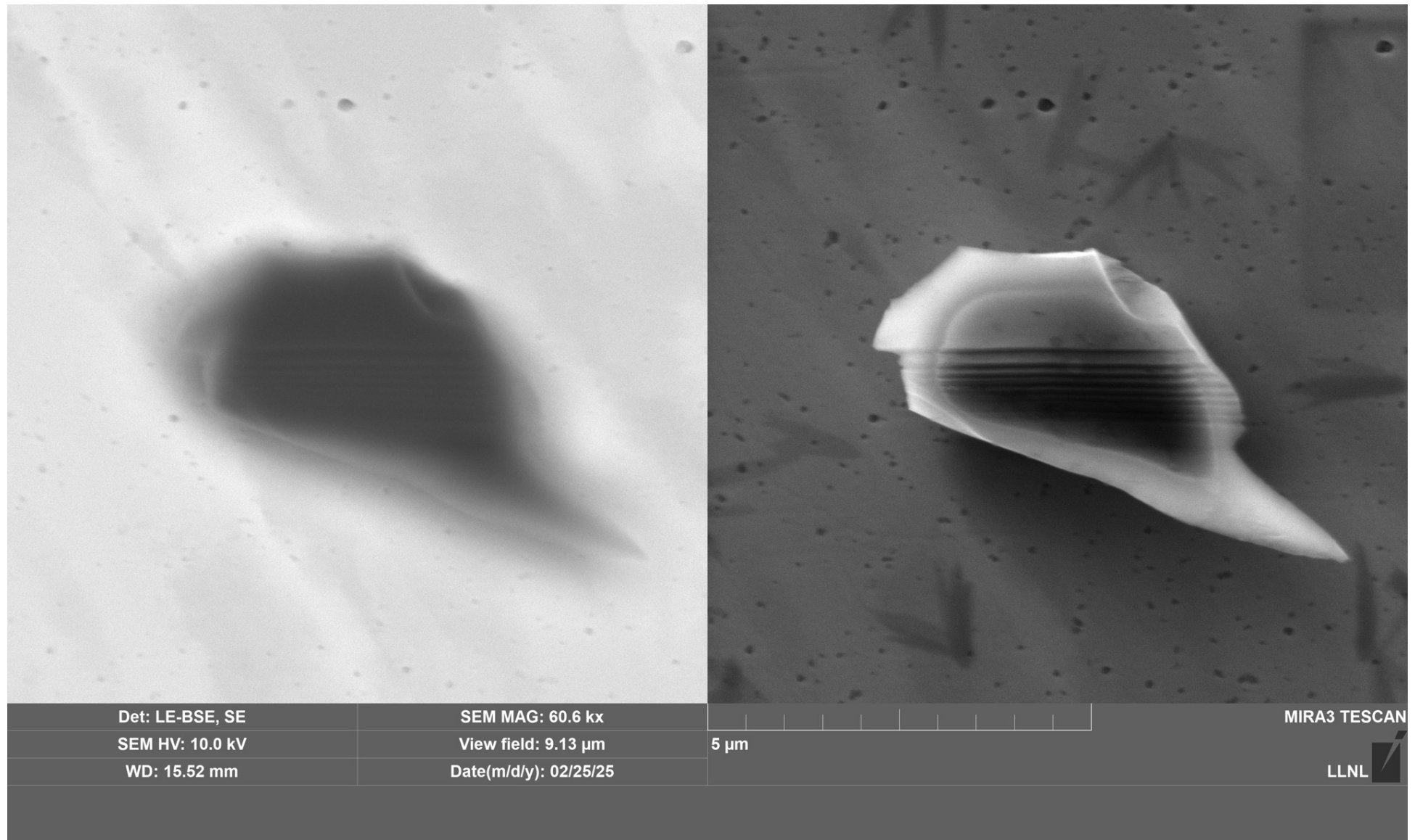

CO_ HE19_03

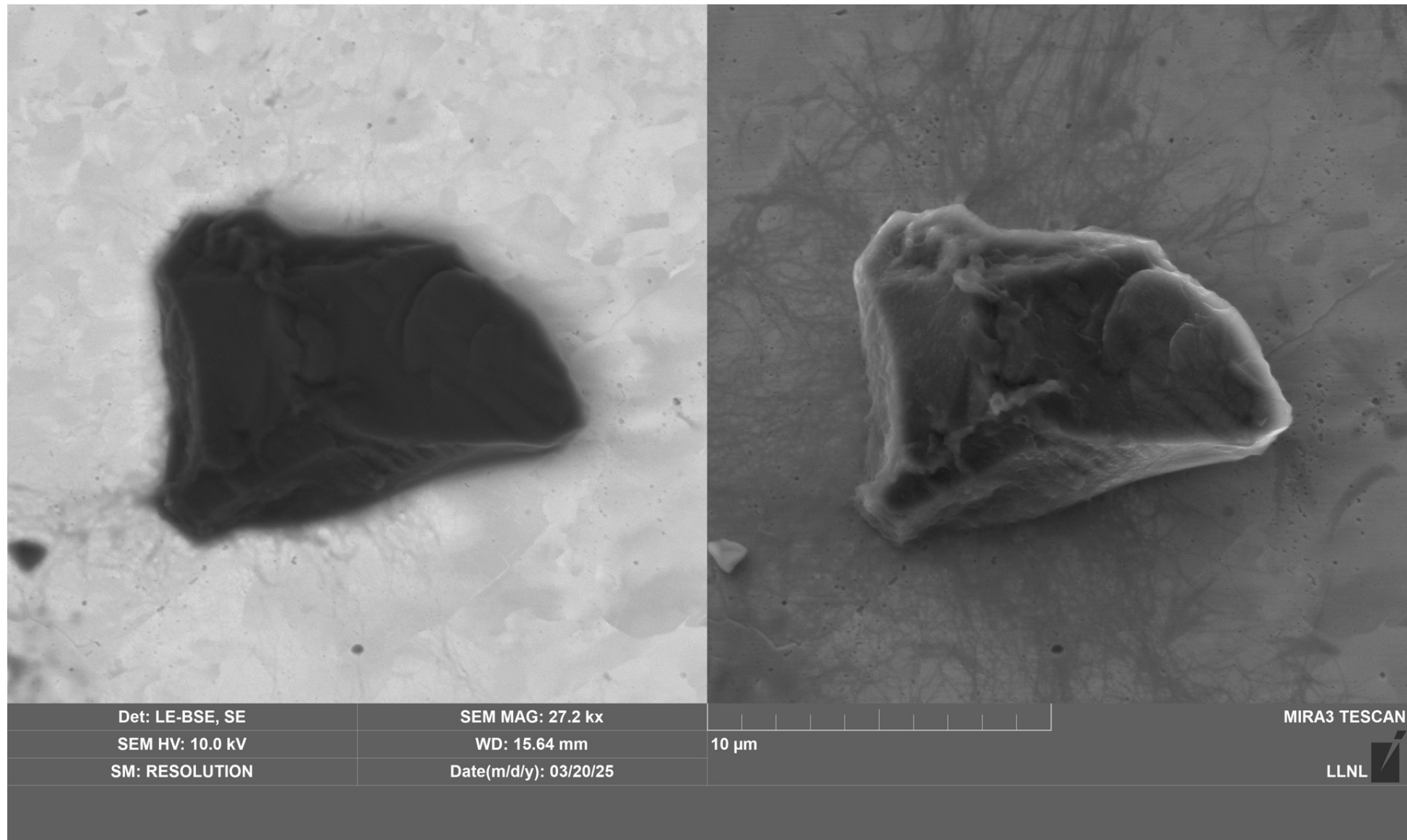

terrestrial

CO_ HE20_02

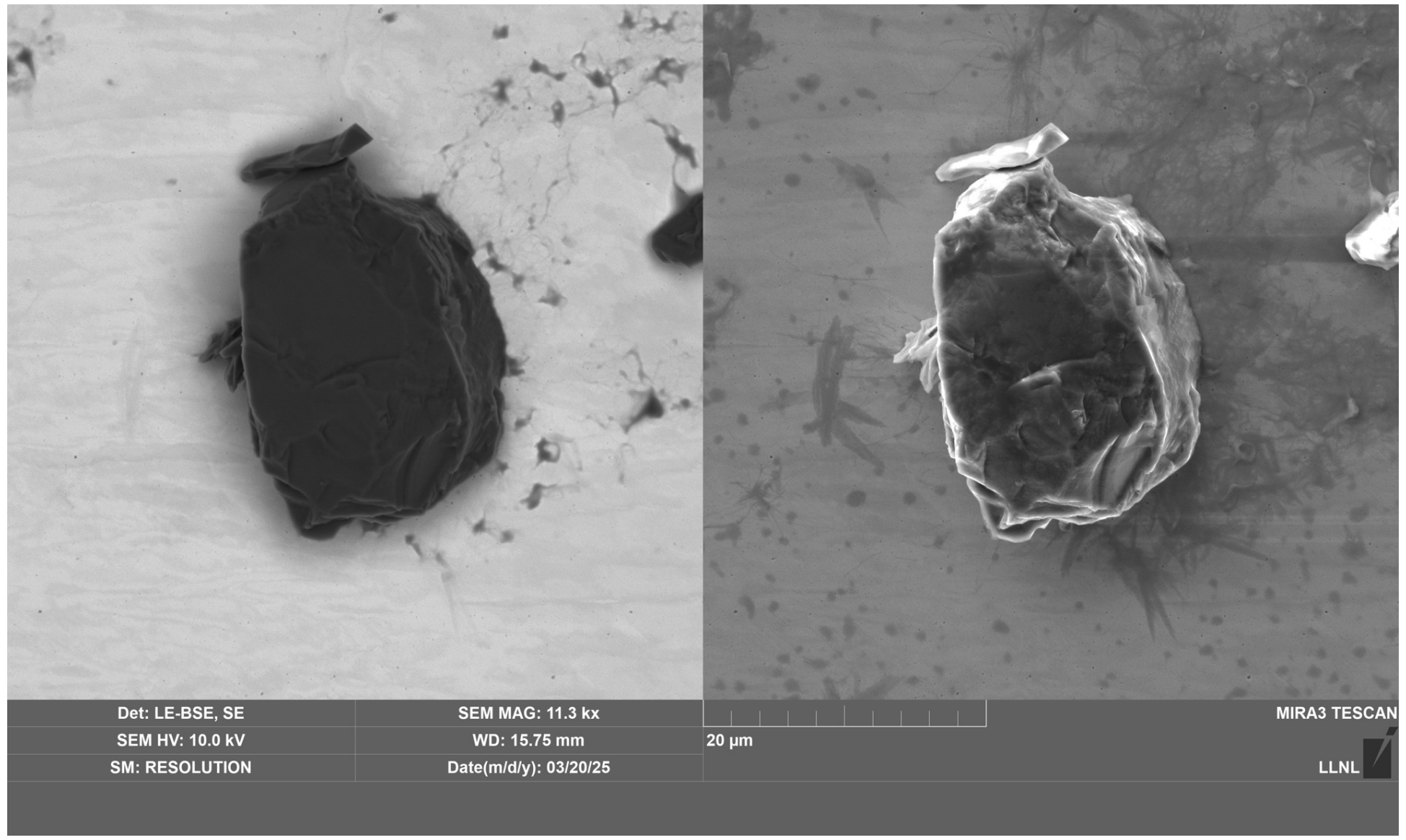

CO_ HE24_02

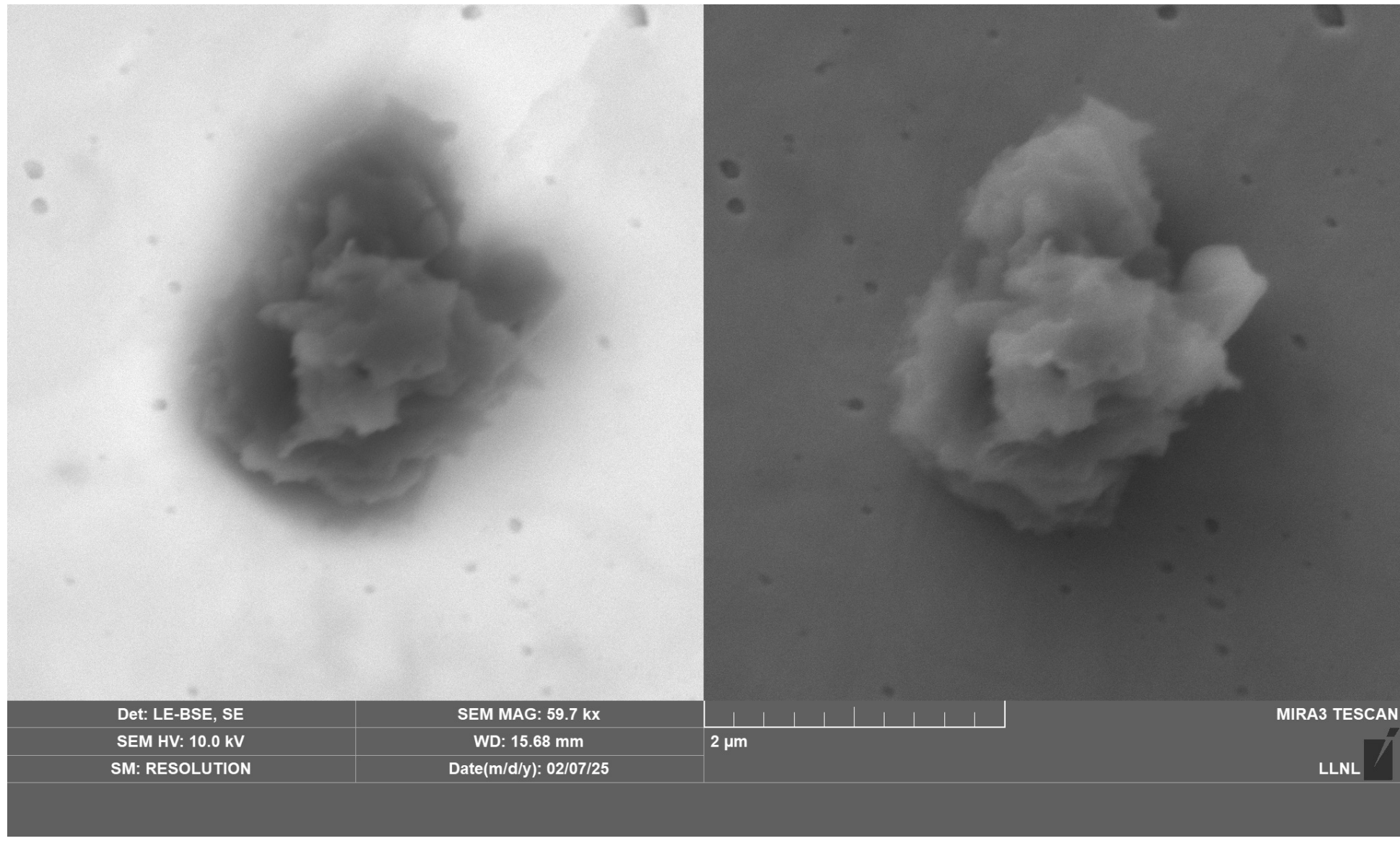

CO_ HE23_05

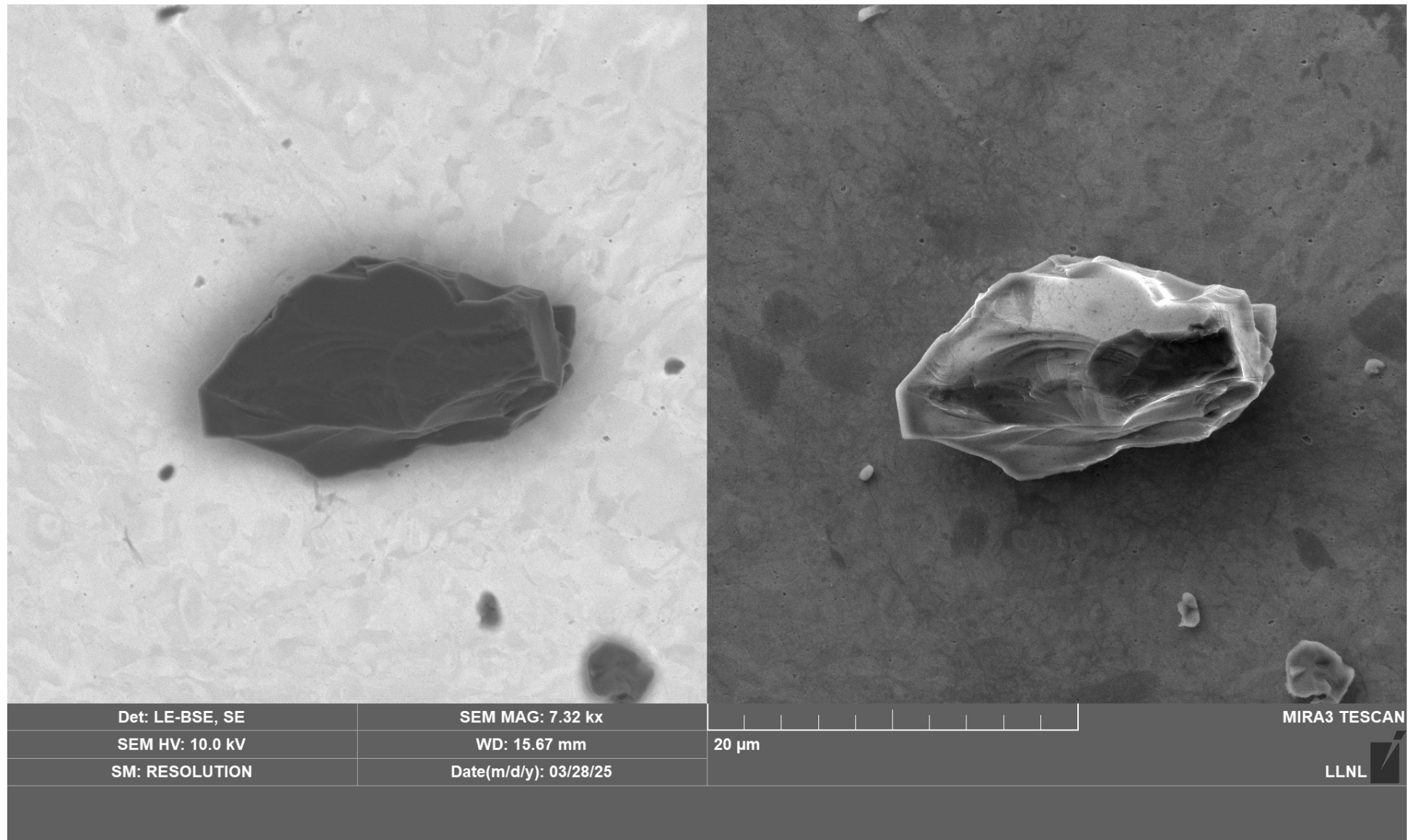

CO_ HE24_03

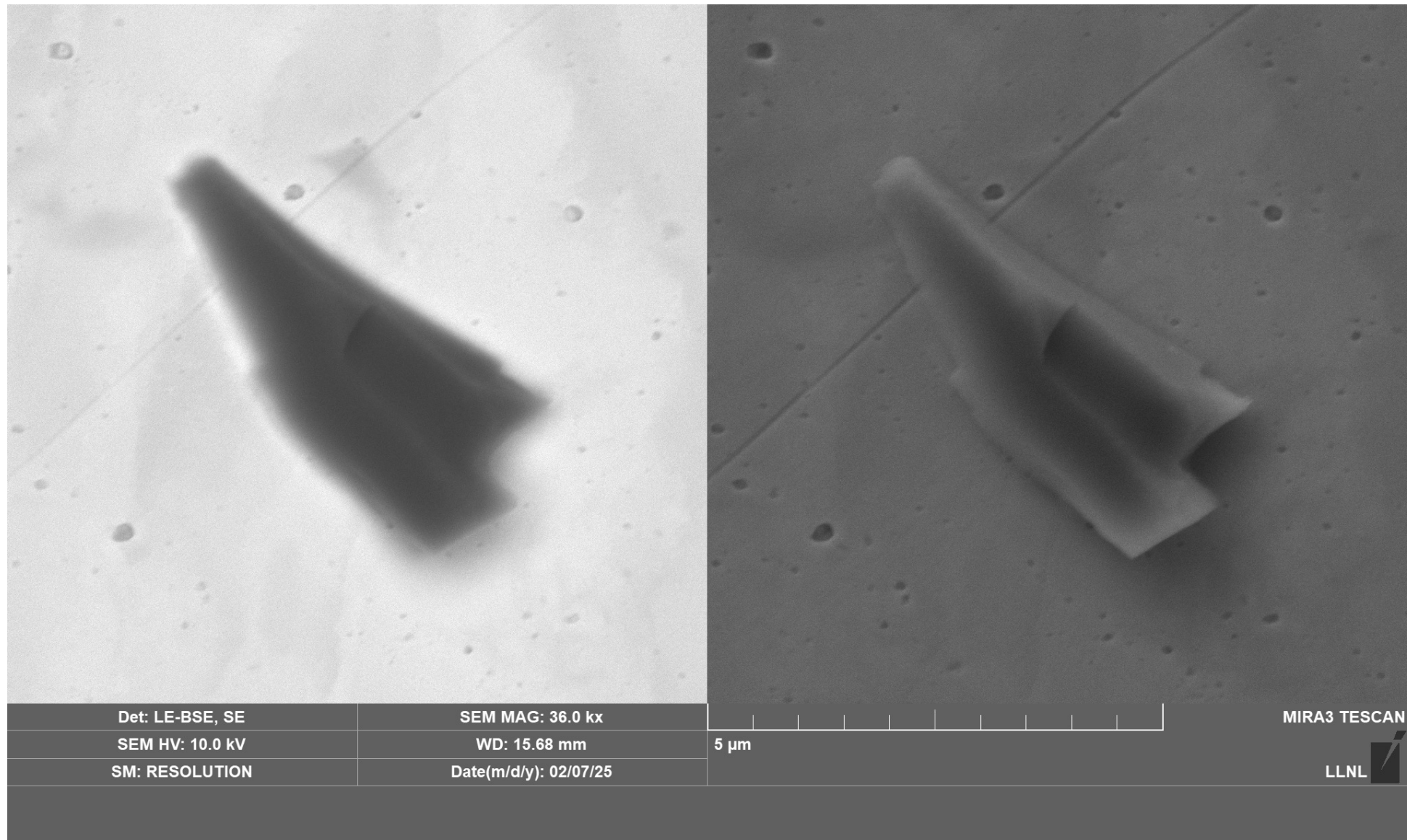

terrestrial

CO_ HE24_05

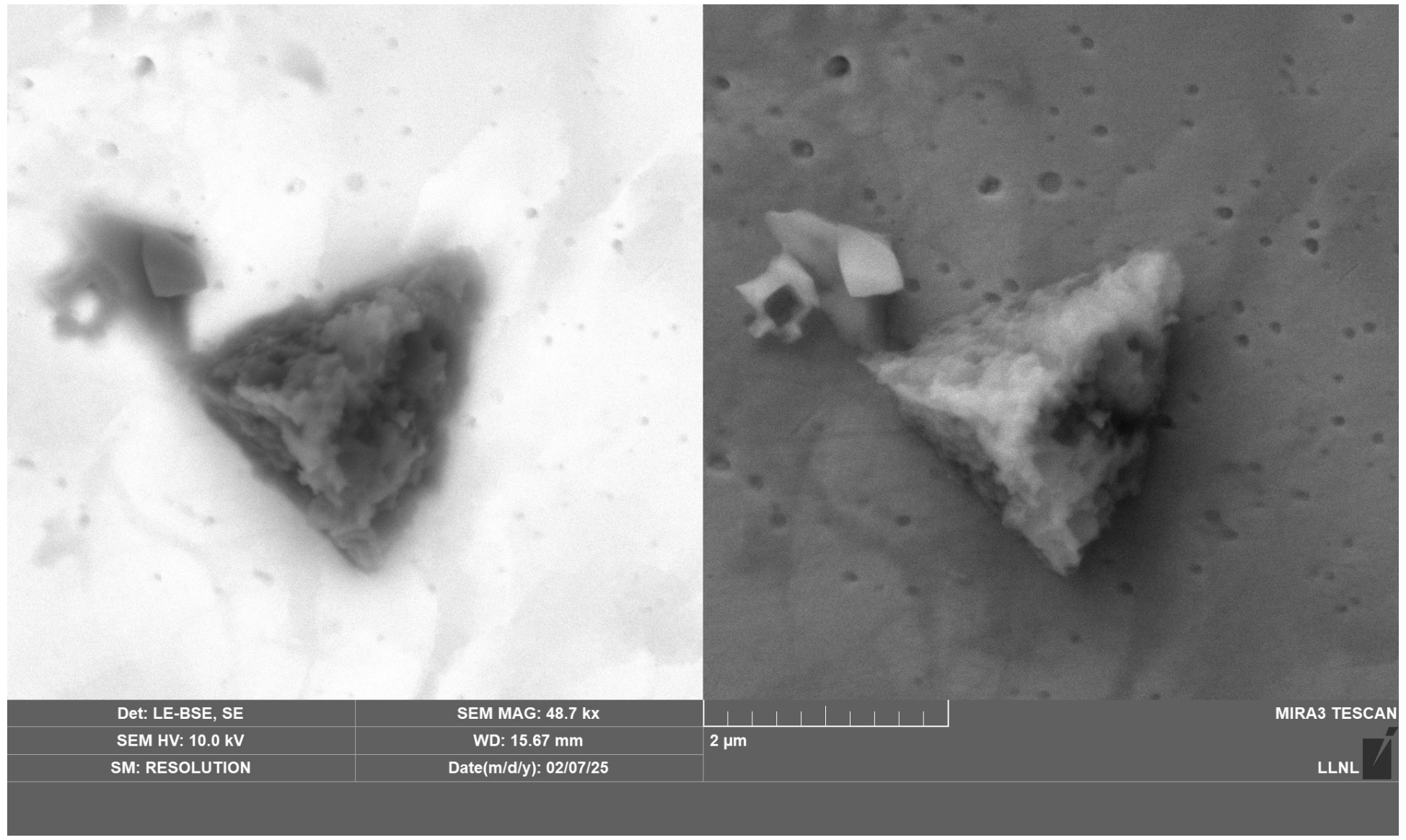

CO_ HE24_07

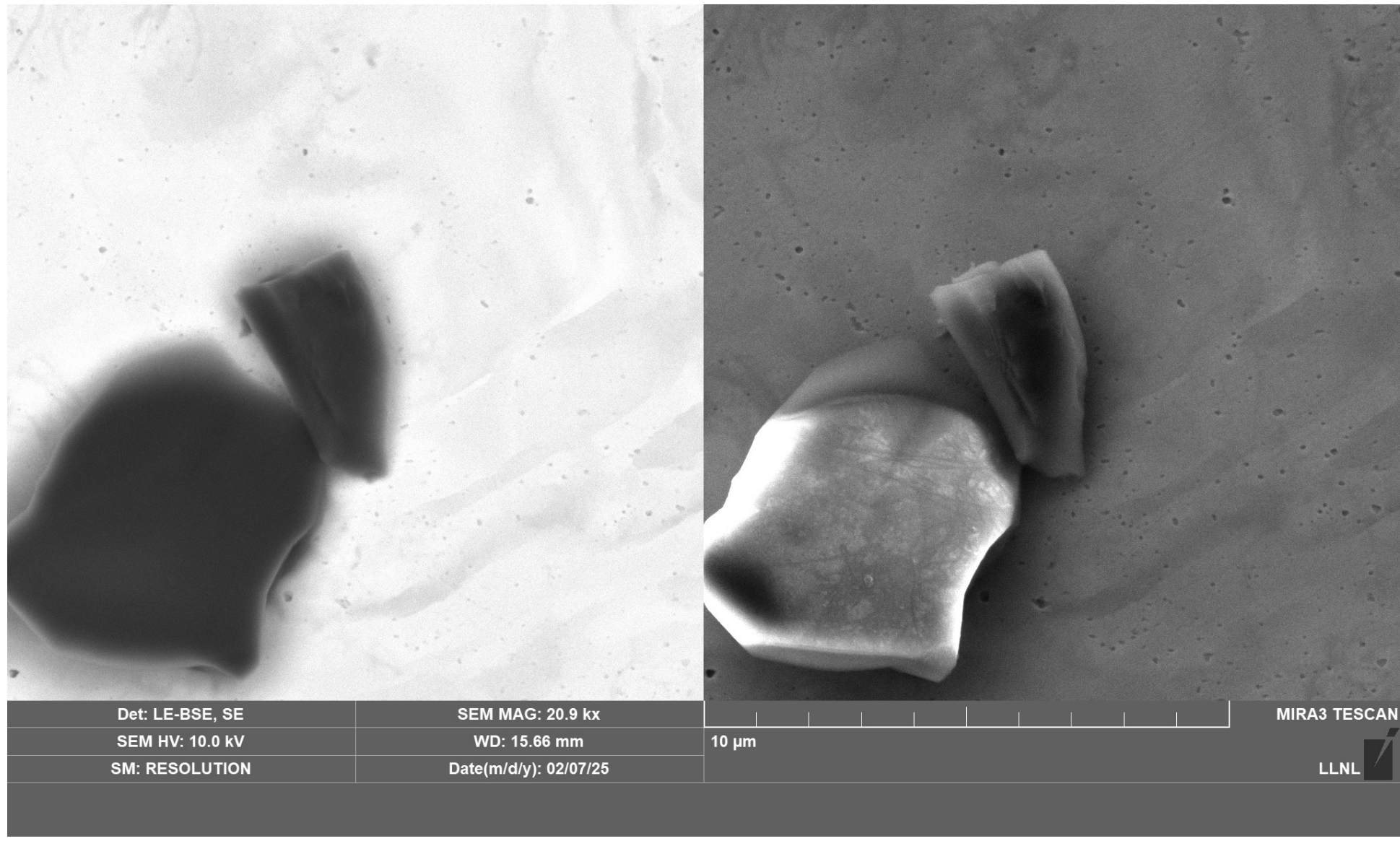

CO_ HE24_06

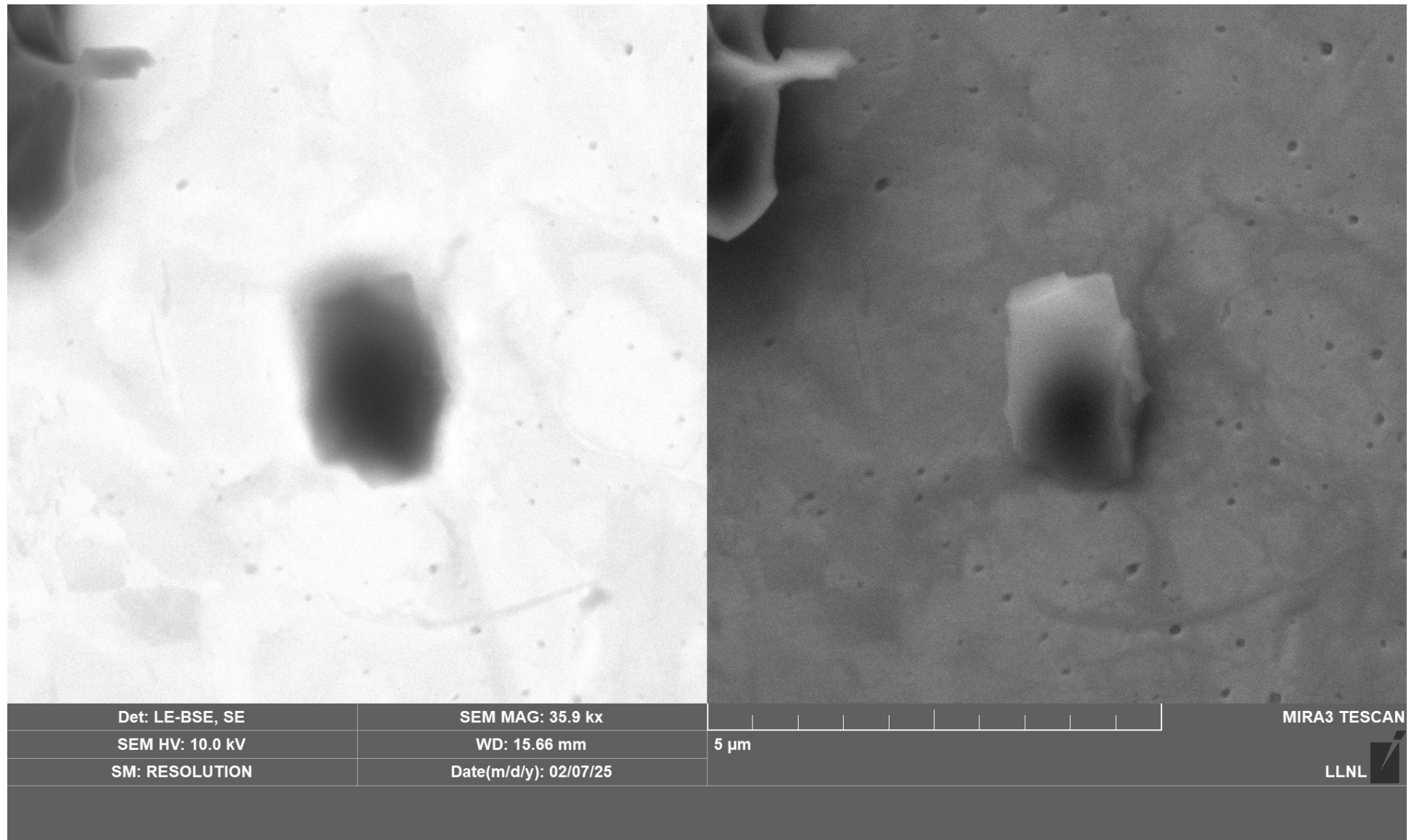

CO_ HE24_09

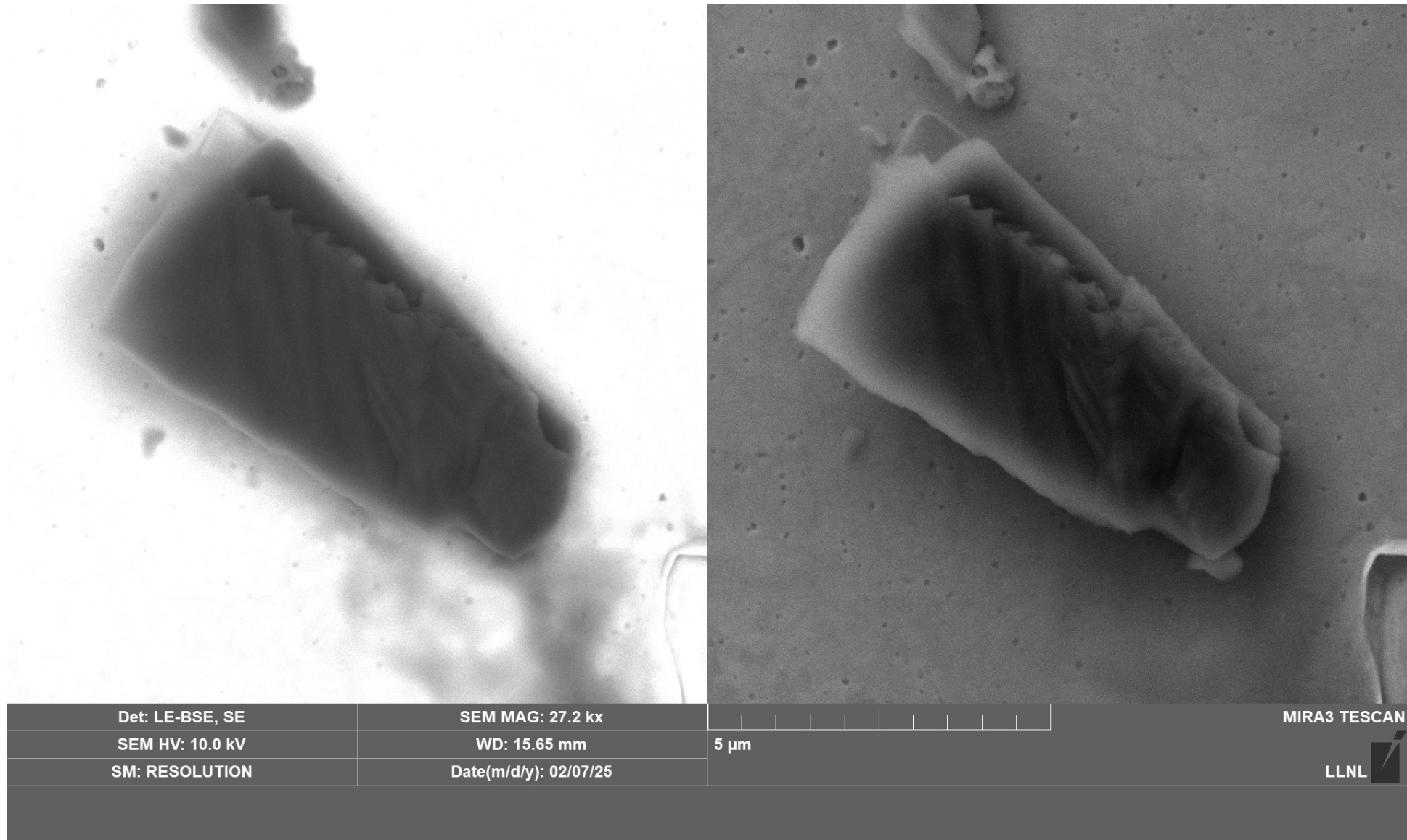

terrestrial

CO_ HE24_12

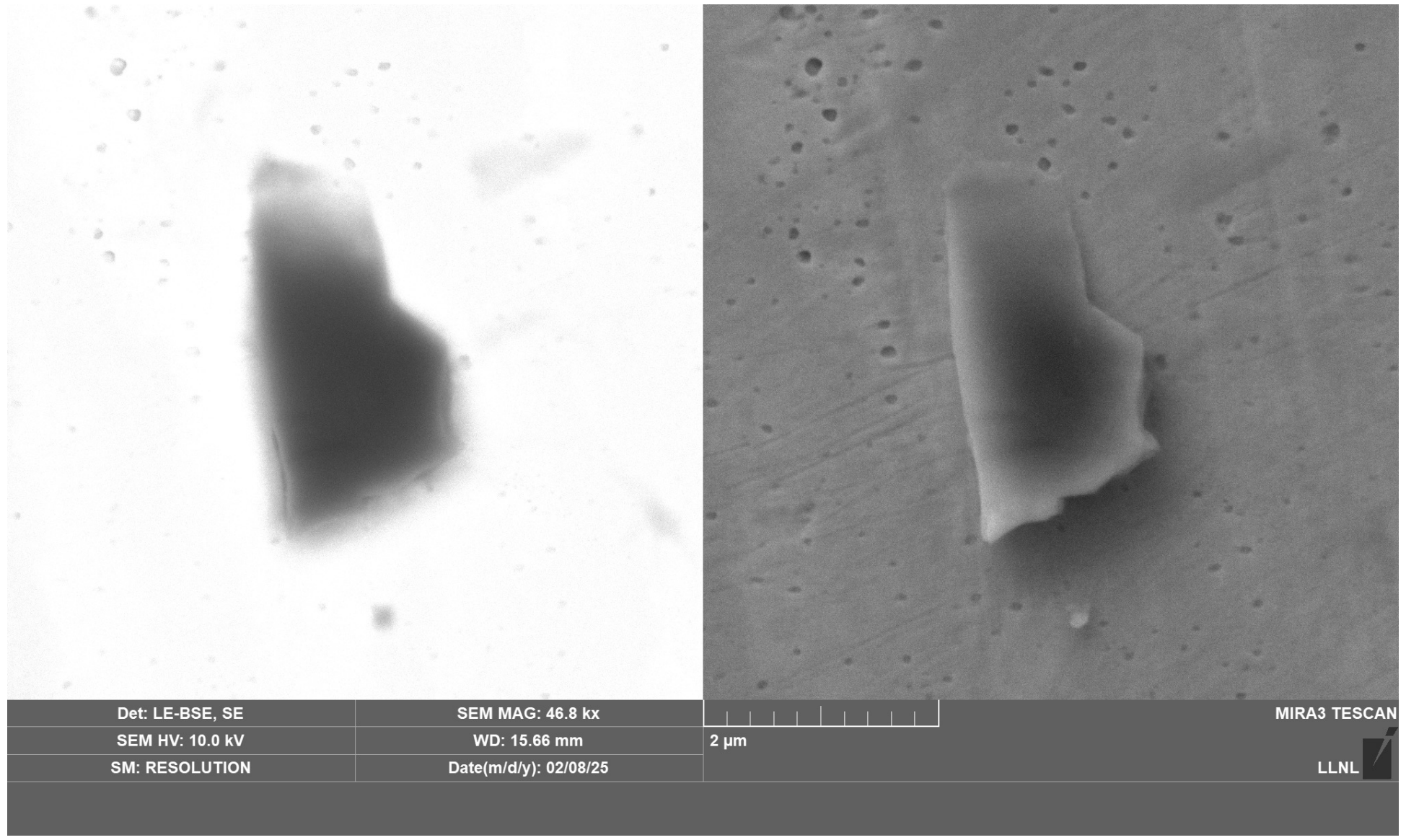

CO_ HE25_06

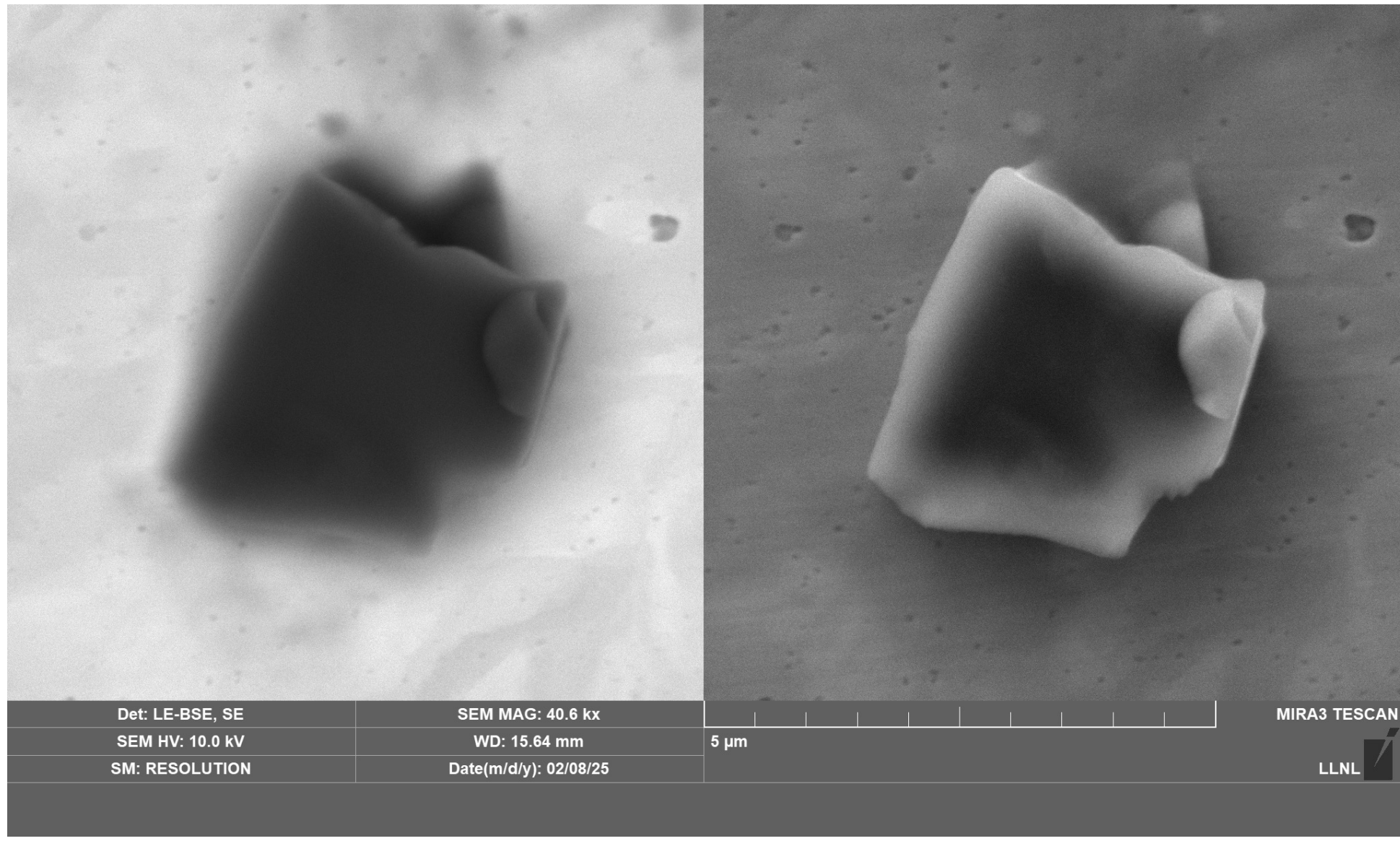

CO_ HE25_05

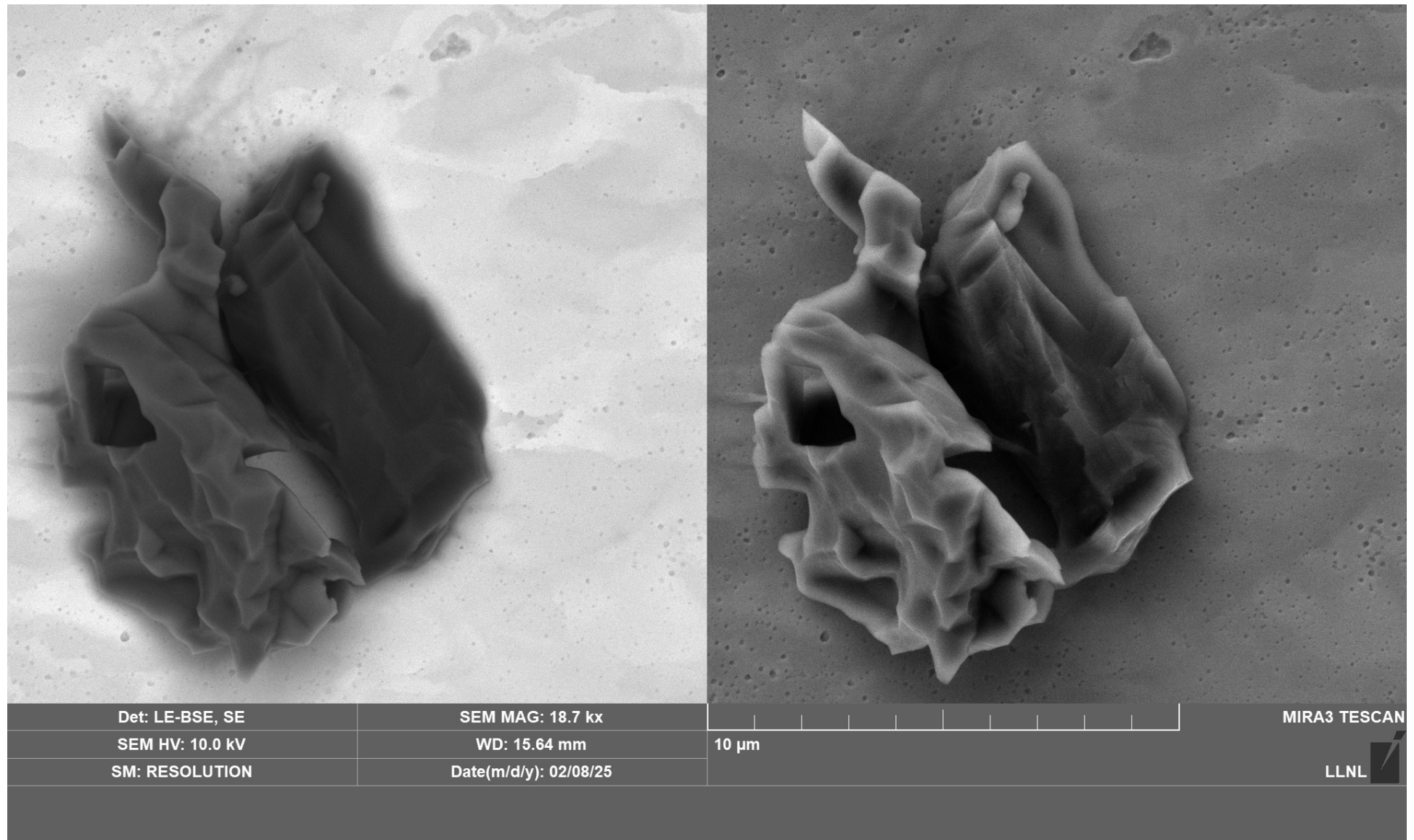

CO_ HE25_10

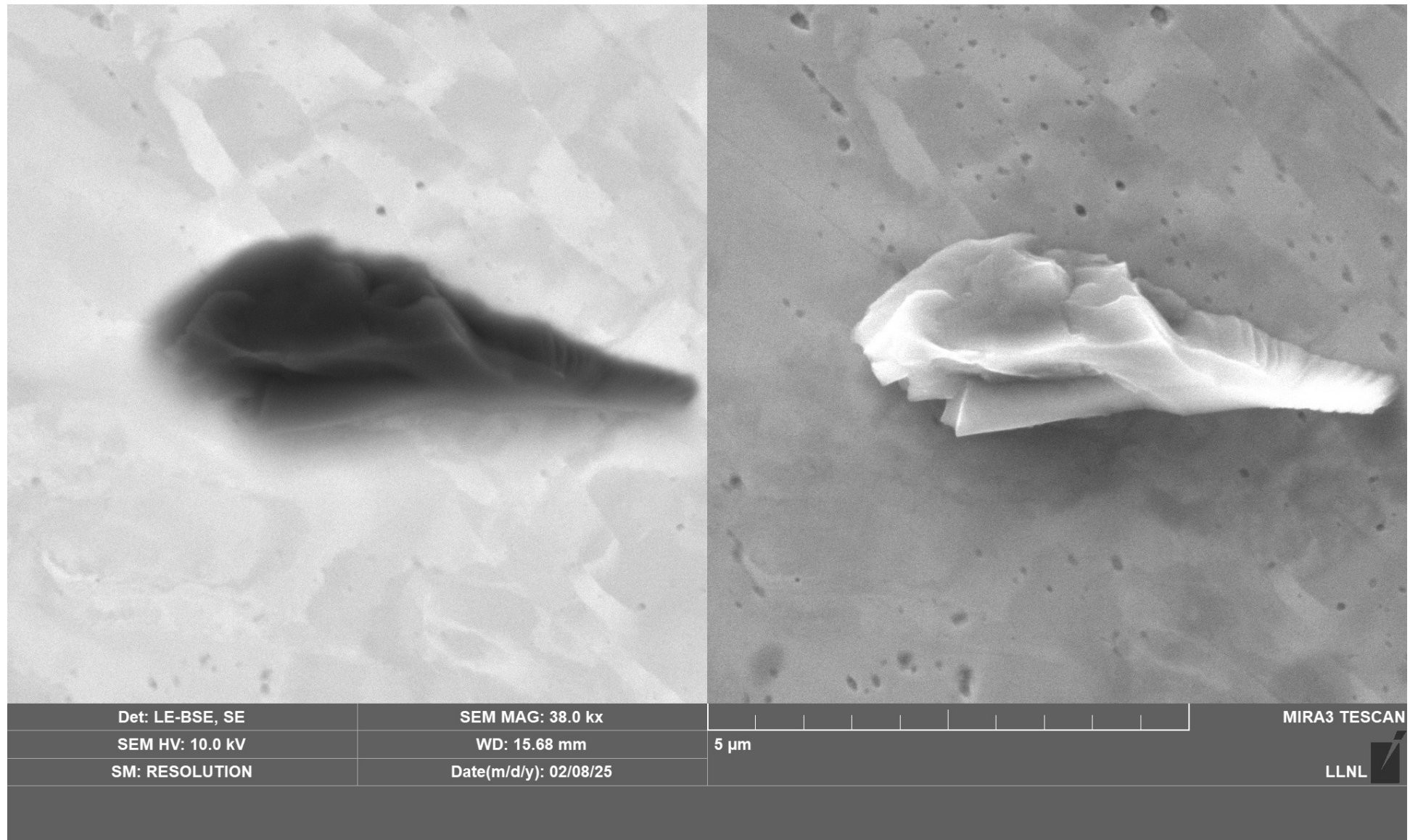

SEM image of NanoSIMS XR prototype individual fixed beam ~25 nm craters
Next to HE29_05

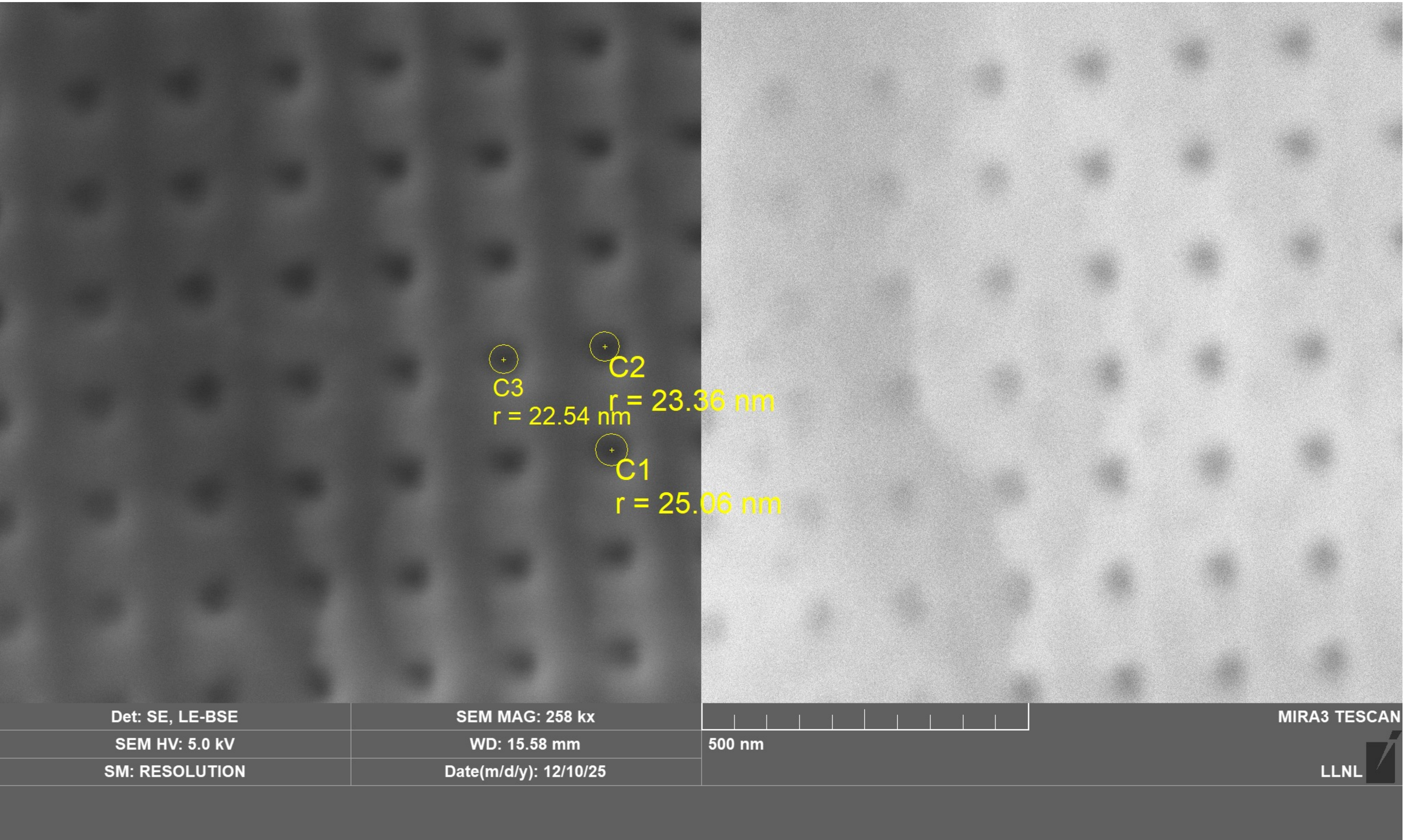

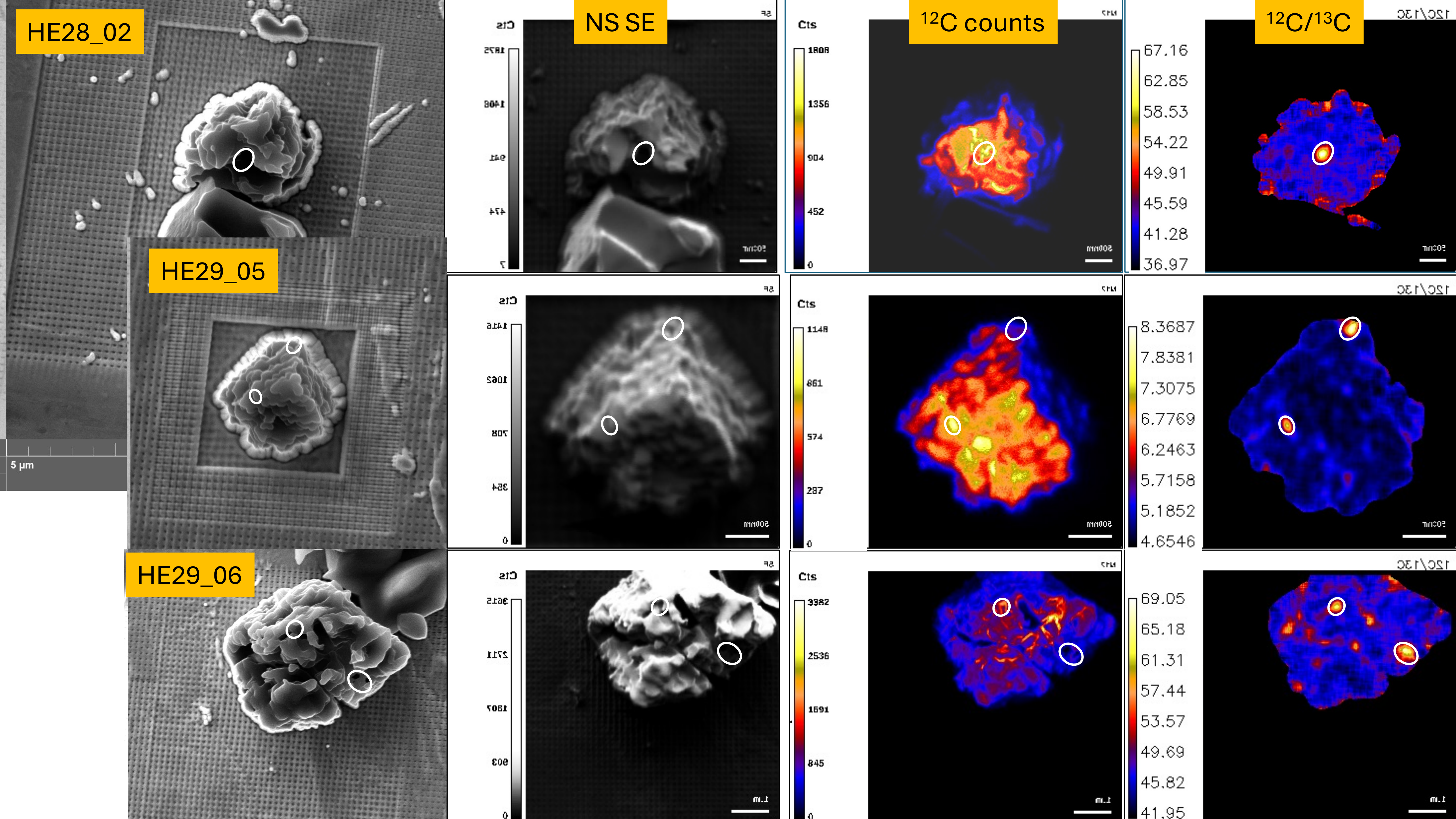
HE28_02
HE29_05
HE29_06
5 µm
NS SE
^{12}C counts
$^{12}C/^{13}C$
Cts
1808
1356
904
452
0
67.16
62.85
58.53
54.22
49.91
45.59
41.28
36.97
Cts
1148
861
574
287
0
8.3687
7.8381
7.3075
6.7769
6.2463
5.7158
5.1852
4.6546
Cts
3382
2536
1691
845
0
69.05
65.18
61.31
57.44
53.57
49.69
45.82
41.95

HE28_02

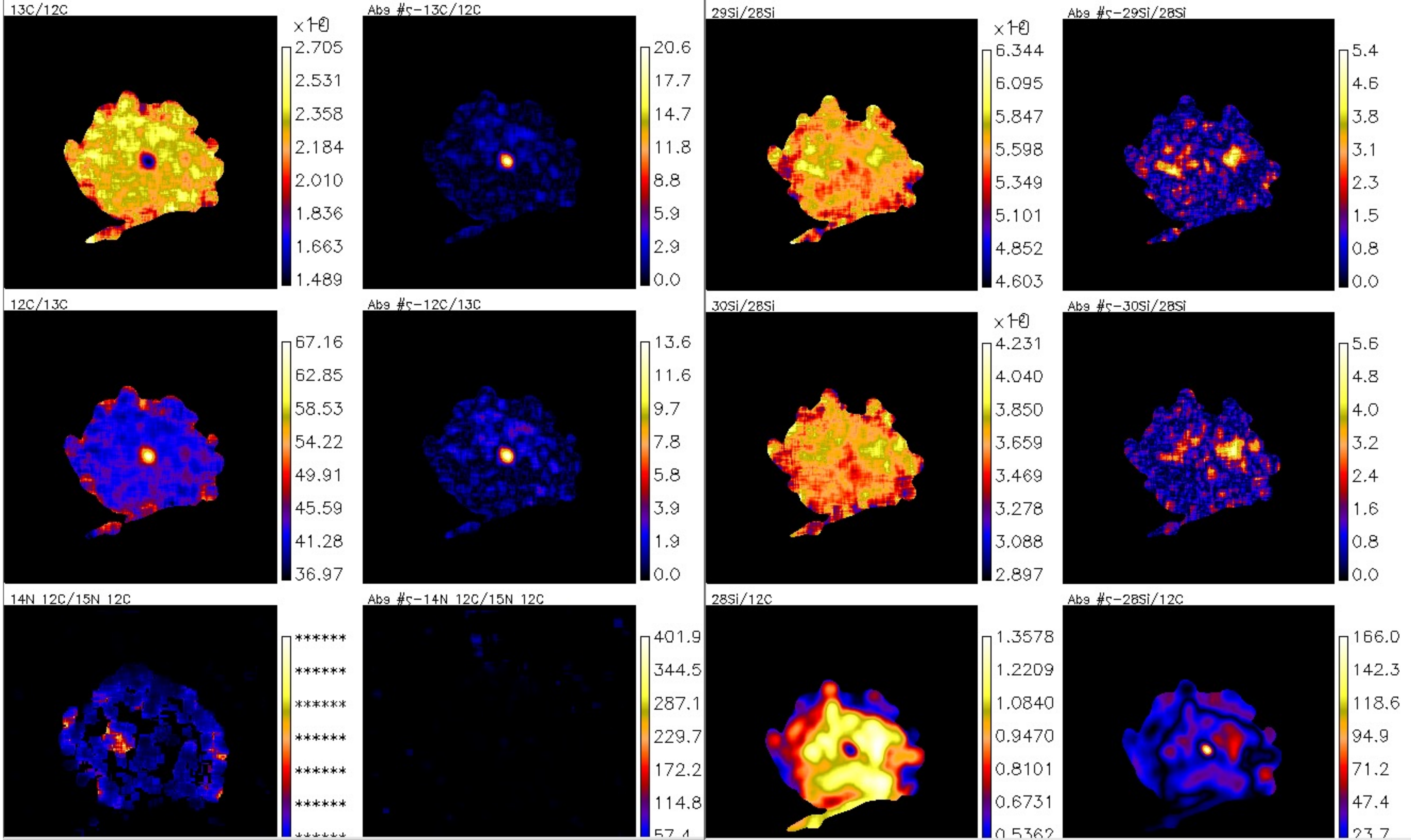
13C/12C
x1E0
2.705
2.531
2.358
2.184
2.010
1.836
1.663
1.489
Abs #ς-13C/12C
20.6
17.7
14.7
11.8
8.8
5.9
2.9
0.0
29Si/28Si
x1E0
6.344
6.095
5.847
5.598
5.349
5.101
4.852
4.603
Abs #ς-29Si/28Si
5.4
4.6
3.8
3.1
2.3
1.5
0.8
0.0
12C/13C
67.16
62.85
58.53
54.22
49.91
45.59
41.28
36.97
Abs #ς-12C/13C
13.6
11.6
9.7
7.8
5.8
3.9
1.9
0.0
30Si/28Si
x1E0
4.231
4.040
3.850
3.659
3.469
3.278
3.088
2.897
Abs #ς-30Si/28Si
5.6
4.8
4.0
3.2
2.4
1.6
0.8
0.0
14N 12C/15N 12C
******
******
******
******
******
******
Abs #ς-14N 12C/15N 12C
401.9
344.5
287.1
229.7
172.2
114.8
28Si/12C
1.3578
1.2209
1.0840
0.9470
0.8101
0.6731
Abs #ς-28Si/12C
166.0
142.3
118.6
94.9
71.2
47.4

HE29_05

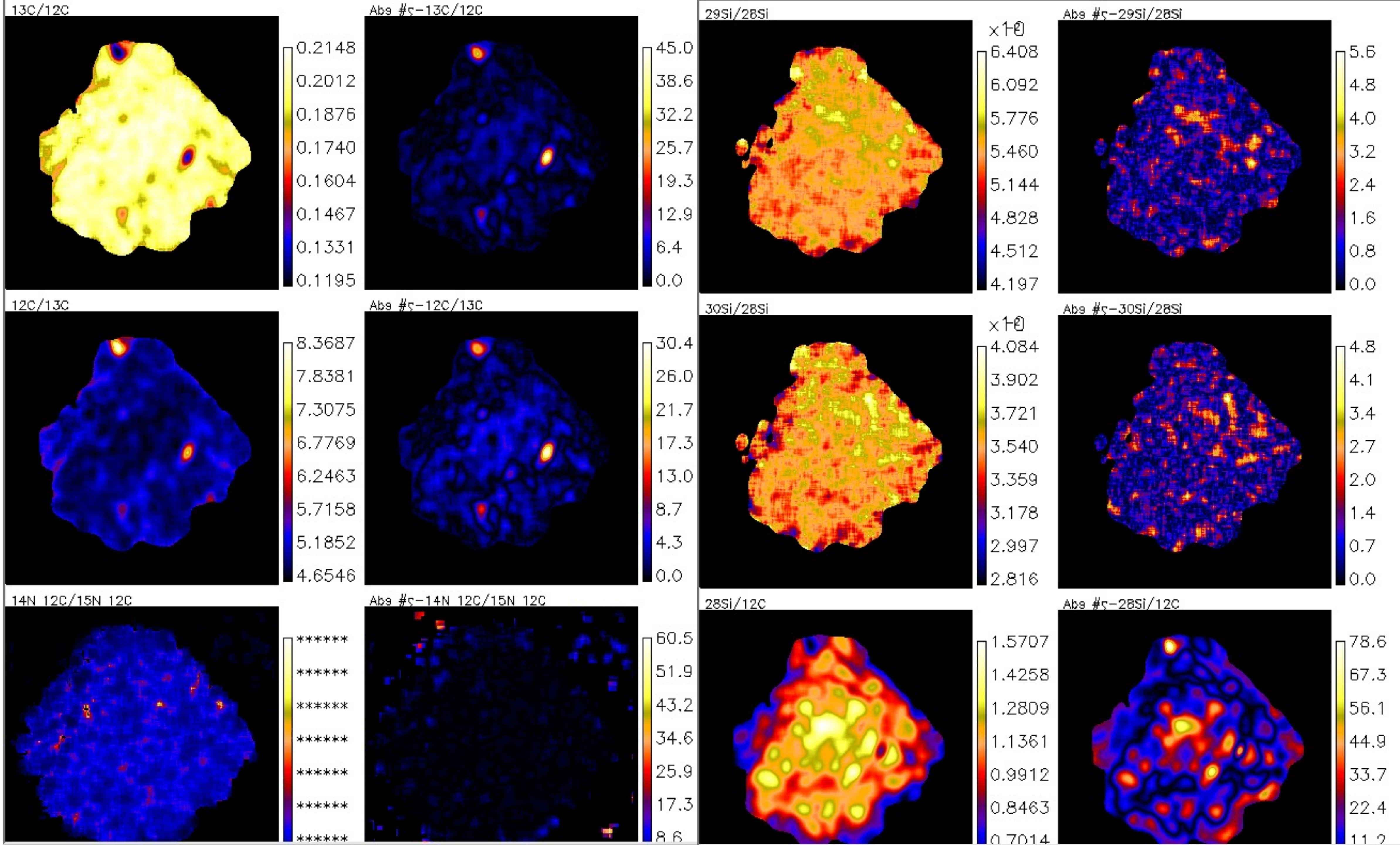

13C/12C
0.2148
0.2012
0.1876
0.1740
0.1604
0.1467
0.1331
0.1195
Abs #ς-13C/12C
45.0
38.6
32.2
25.7
19.3
12.9
6.4
0.0
29Si/28Si
6.408
6.092
5.776
5.460
5.144
4.828
4.512
4.197
Abs #ς-29Si/28Si
5.6
4.8
4.0
3.2
2.4
1.6
0.8
0.0
12C/13C
8.3687
7.8381
7.3075
6.7769
6.2463
5.7158
5.1852
4.6546
Abs #ς-12C/13C
30.4
26.0
21.7
17.3
13.0
8.7
4.3
0.0
30Si/28Si
4.084
3.902
3.721
3.540
3.359
3.178
2.997
2.816
Abs #ς-30Si/28Si
4.8
4.1
3.4
2.7
2.0
1.4
0.7
0.0
14N 12C/15N 12C
******
******
******
******
******
******
******
Abs #ς-14N 12C/15N 12C
60.5
51.9
43.2
34.6
25.9
17.3
8.6
28Si/12C
1.5707
1.4258
1.2809
1.1361
0.9912
0.8463
0.7014
Abs #ς-28Si/12C
78.6
67.3
56.1
44.9
33.7
22.4
11.2

HE29_06

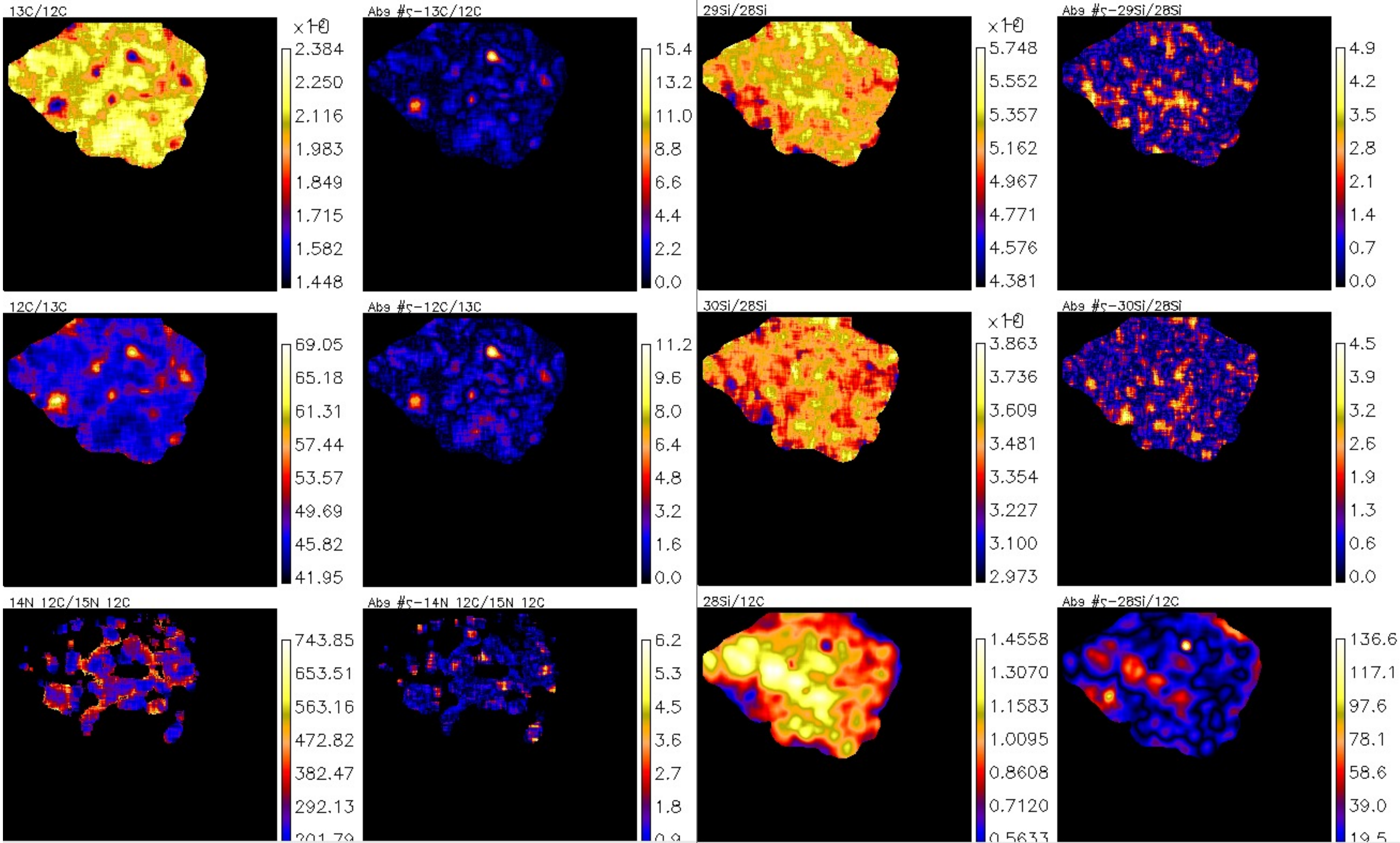
13C/12C
x10
2.384
2.250
2.116
1.983
1.849
1.715
1.582
1.448
Abs #ς-13C/12C
15.4
13.2
11.0
8.8
6.6
4.4
2.2
0.0
29Si/28Si
x10
5.748
5.552
5.357
5.162
4.967
4.771
4.576
4.381
Abs #ς-29Si/28Si
4.9
4.2
3.5
2.8
2.1
1.4
0.7
0.0
12C/13C
69.05
65.18
61.31
57.44
53.57
49.69
45.82
41.95
Abs #ς-12C/13C
11.2
9.6
8.0
6.4
4.8
3.2
1.6
0.0
30Si/28Si
x10
3.863
3.736
3.609
3.481
3.354
3.227
3.100
2.973
Abs #ς-30Si/28Si
4.5
3.9
3.2
2.6
1.9
1.3
0.6
0.0
14N 12C/15N 12C
743.85
653.51
563.16
472.82
382.47
292.13
201.79
Abs #ς-14N 12C/15N 12C
6.2
5.3
4.5
3.6
2.7
1.8
0.9
28Si/12C
1.4558
1.3070
1.1583
1.0095
0.8608
0.7120
0.5633
Abs #ς-28Si/12C
136.6
117.1
97.6
78.1
58.6
39.0
19.5
